# Structure-activity relationships of 1,5-dihydro-2*H*-benzo[*b*][1,4]diazepine-2,4(3*H*)-diones as inhibitors of *Trypanosoma cruzi*

**DOI:** 10.1039/d5md00185d

**Published:** 2025

**Authors:** Michael G. Thomas, Joanne Dunne, Peter G. Dodd, Emiliana D’Oria, Laura Frame, Adolfo Garcia-Perez, Kate McGonagle, Pilar Manzano, Lorna MacLean, Christy Paterson, Jennifer Riley, John Thomas, Leah S. Torrie, Karolina Wrobel, Kevin D. Read, Maria Marco, Manu De Rycker

## Abstract

Chagas disease, caused by infection with the protozoan parasite *Trypanosoma cruzi (T. cruzi)*, is responsible for a large health burden with around 6 – 8 million people infected globally. The current drug development pipeline is sparsely populated and there is an urgent need for new treatments. In this paper we describe the identification of a series of benzodiazepinediones with antiparasitic activity, and the platform we utilised to demonstrate that they act via a novel mechanism of action. Two distinct sub-series were identified, and the medicinal chemistry program identified compounds from both with pIC_50_’s > 6.4 which were progressed to a *T. cruzi* washout assay. This assay showed that neither sub-series was suitable for further development. This work demonstrated the value of a robust Chagas disease discovery platform for focusing the limited resources available for neglected disease drug discovery onto the most promising series.

## Graphical abstract

**Figure:**
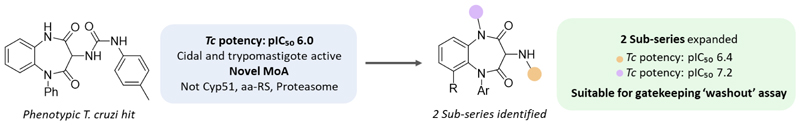

## Introduction

The development of tractable chemical series leading to new treatments for Chagas disease is a challenging endeavour, due to the complexity of the disease, a lack of validated biochemical targets and a challenging drug development pathway.

Chagas disease is caused by infection with the protozoan parasite *Trypanosoma cruzi (T. cruzi)*. ^1–3^ There are an estimated 6-8 million people infected globally and it is responsible for approximately 10,000 deaths per annum. The disease is endemic in many countries in Latin America, but migration from those countries means it is also present in many other countries globally. The infection is transmitted *via* several different routes, notably from the faeces of triatomine (or kissing) bugs, as well as through contaminated foods, blood transfusions and from mother to foetus. ^1–3^

Chagas disease progresses through three phases where an initial acute phase, characterised by non-specific flu-like symptoms, is followed by a chronic, asymptomatic phase. ^1,3^ Anywhere from 10 – 30 years later, for 10 – 30% of patients, the chronic symptomatic phase occurs, where heart and gastrointestinal tract problems can eventually be fatal.

There are currently only two treatments for Chagas disease, benznidazole and nifurtimox, and both have associated problems such as long (60 – 90 day) treatments and side-effects which frequently lead to premature discontinuation of treatment. ^4,5^ A recent clinical trial (BENDITA trial, NCT03378661) ^6^ did demonstrate the potential of using improved dosing regimens for benznidazole but alternative medicines remain unavailable.

The development pipeline for new drugs is sparse, with the most advanced programmes targeting the parasite proteasome, ^7,8^ topoisomerase II ^9^ and CLK1 ^10^ as well as a combination treatment of a low dose of benznidazole with a cytochrome b inhibitor. ^11^

**Fig. 1:**
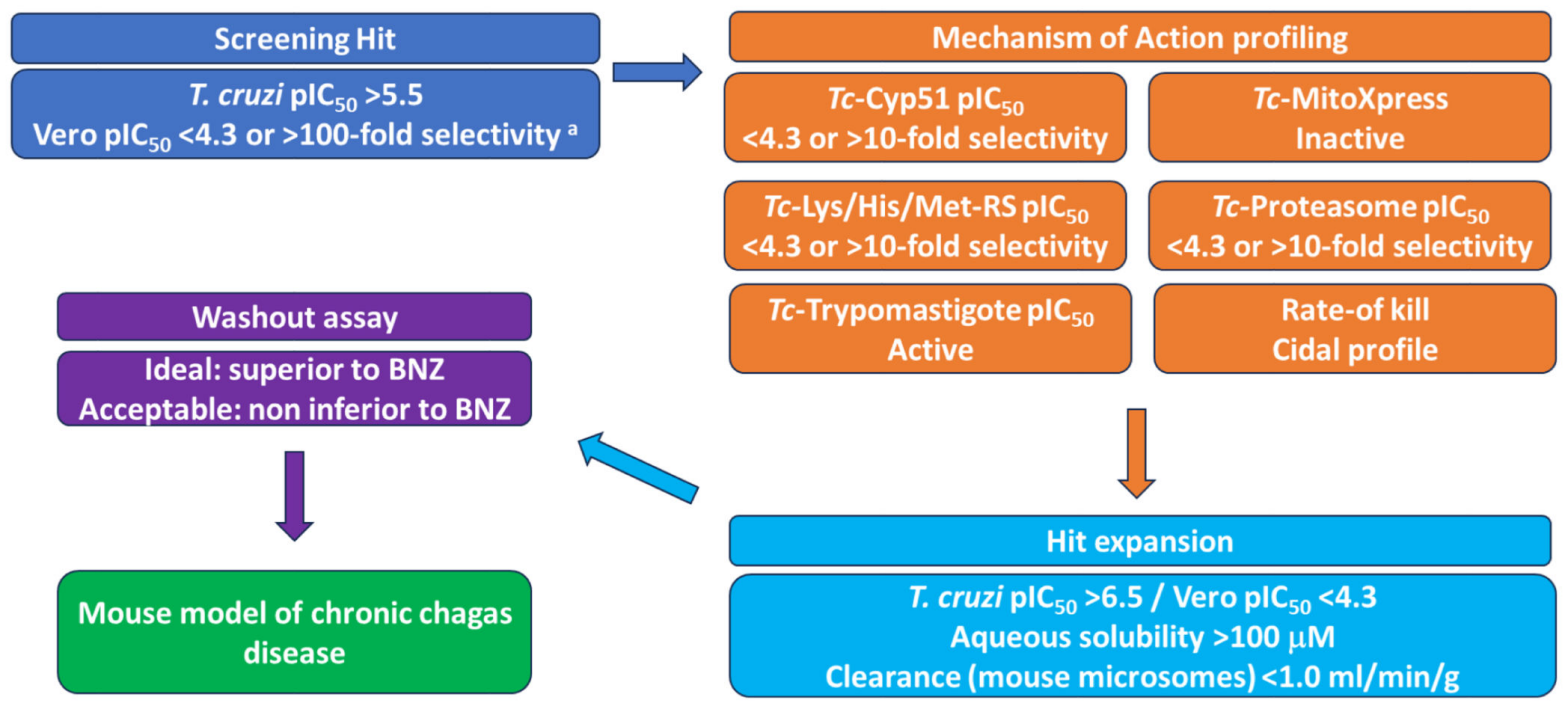
Screening cascade for the identification of compound series for progression against *T. cruzi*. ^a^ Selectivity refers to *T. cruzi* vs Vero activity in the cell-based *T. cruzi* assay.

To identify new starting points for Chagas disease drug discovery we developed a screening cascade for identifying antiparasitic compounds with novel mechanisms of action, based on cell-based high-throughput screening followed by known mode of action profiling (Fig. 1). This platform aims to discover new starting points for drug discovery differentiated from known, undesirable mechanisms or those currently in our development pipeline, potentially diversifying the treatments available.

In our cascade, hits are identified using a high-content imaging assay for intracellular *T. cruzi* amastigotes. ^12^ Active compounds are then screened against known undesirable mechanisms of action. Firstly, compounds are screened in a *T. cruzi* C14α demethylase (*Tc*-CYP51) assay. ^13^ This is a promiscuous target and two *Tc*-CYP51 inhibitors, posaconazole and fosravuconazole, progressed to clinical trials where they led to treatment failures in 70 – 90% of patients. ^14,15^ Alongside this, compounds are screened in a *T. cruzi* oxygen consumption assay to identify and deprioritise compounds acting by disruption of the parasite electron transport chain (ETC). ^16,17^ While a preclinical candidate has been identified for use in combination treatments, compounds inhibiting the parasite ETC, particularly complex III, have previously been shown to be non-efficacious as monotherapy treatments. ^11^ To assess if hits act through targets that we have previously explored, they are profiled biochemically against the *T. cruzi* proteasome ^18^ and methionyl-, histidyl-and lysyl-tRNA synthetase ^19^. For any compounds with a distinct mechanism of action, activity against *T. cruzi* trypomastigotes, the non-replicative transmission stage of the parasite, is also determined. ^12^ While activity against trypomastigotes may not be a prerequisite for a new Chagas disease treatment, we seek to prioritise such compounds to increase the probability of success, as posaconazole is only very weakly active against trypomastigotes, whereas benznidazole and nifurtimox retain good activity. ^12^ Finally, we determine the rate-of-kill against intracellular amastigotes to remove any growth-arresting compounds and prioritise fast-acting cytocidal compounds. ^20^

For any hits identified and successfully triaged, the initial focus of hit-expansion is to identify compounds with a suitable profile to run in a washout assay. ^12,21^ In this assay, intracellular *T. cruzi* are treated with high doses of compounds for an extended duration (25x IC_50_ for 8 – 16 days), followed by compound washout and assessment of parasite relapse over 60 days to assess ability to clear all parasites, including any persisters. Because of these high doses, compounds ideally have *T. cruzi* pIC_50_ >6.0, host Vero cell pIC_50_ <4.3, aqueous solubility >100 μM and mouse microsomal clearance <1.0 ml/min/g (as the Vero host cells exhibit some level of metabolism). Compound series with a profile equal to or better than benznidazole can then be progressed to an *in vivo* efficacy model of chronic Chagas disease ^22^ with a high probability of success.

## Results and Discussion

From a screen of a selection from the GSK diversity collection, compound **1** (Table 1) was identified as a potentially interesting hit compound with activity against intracellular *T. cruzi*. Compound **1** was therefore triaged through our hit assessment platform and the full profile is shown in Table 1. The pIC_50_ of 6.0 was close to the targeted value of 6.5 and it was reassuring to see no evidence of activity against the host Vero cells. Compound **1** was inactive in the mitochondrial oxygen consumption assay and against the tested *T. cruzi* tRNA synthetases. Compound **1** did show weak potency against the *T. cruzi* proteasome, but based on the biochemical vs. cellular potency it was unlikely to be the main driver of the antiparasitic effect. It also showed weak activity against *Tc*-CYP51, but the high activity against *Tc*-trypomastigotes suggested that this was not driving antiparasitic activity.

One issue highlighted was the low aqueous solubility (35 μM compared to the targeted 100 μM), potentially driven in part by the high lipophilicity (ChromLogD_pH7.4_ 4.6) ^23^, which coupled with its three aromatic rings led to a high PFI value of 7.6. PFI (property forecast index, ChromLogD_pH7.4_ + No. Aromatics) has been shown to give a reasonable guide to the potential for aqueous solubility, as well as general developability. ^23^ Alongside PFI, LLE (ligand lipophilic efficiency, *T. cruzi* pIC_50_ - ChromLogD_pH7.4_) was also monitored to identify changes where the impact on potency was not driven by increasing lipophilicity.

The initial aim of the chemistry program was to identify a suitable compound to run in the washout assay, as a precursor to working towards *in vivo* proof of concept studies. As the mechanism of action was unknown, interpretation of SAR (structure-activity relationship) was driven using potency data from the phenotypic high-content imaging assay, with a focus on increasing potency and solubility for progression to the *T. cruzi* washout assay.

We initially focused on exploring the SAR of the different aromatic rings of **1** (Table 2) and at this stage we synthesised all compounds as racemates, with the intent of separating the single enantiomers when more interesting compounds were identified. Modifications to the urea substituent included replacing the 4-methyl group with a 4-methoxy group to give **2**; this led to a 0.5 log unit drop in ChromLogD_pH7.4_, but with no impact on potency, although with a small improvement in solubility. Replacement of 4-methyl with 4-fluoro to give **3** led to a 10-fold drop of potency against the parasites and no improvement in other properties. All other changes made to the urea portion, including alternate substituents and differing substitution patterns led to a loss of activity (data not shown).

Moving on to the 1-phenyl ring (Table 2), various changes were explored. Introducing a 4-methoxy group to give **4** had no impact on potency or metabolic stability and a small increase in solubility, whereas the 3-methoxy group of **5** led to a 0.5 log unit drop in potency, and the 2-methoxy group of **6** led to a further log unit drop (albeit with an improvement in solubility). In an attempt to improve the overall properties of the series, we investigated the replacement of the 1-phenyl ring with alkyl groups to see whether this would improve solubility whilst also retaining potency. This led to the *iso*propyl substituted analogue **7** and the 4-tetrahydropyranyl substituted **8**. Unfortunately, both compounds were a log unit less potent than **1**, although **8** did show a marked improvement in aqueous solubility (380 μM compared to 35 μM). Finally, introduction of a 9-fluoro substituent on the phenyl ring of the bi-cycle (R^3^ in table 2) combined with the previously identified 4-methoxyphenyl urea led to **9**, which gave a small increase in potency, alongside a small drop in ChromLogD_pH7.4_. Despite an improvement in LLE, this led to a decrease in solubility.

**Fig. 2:**
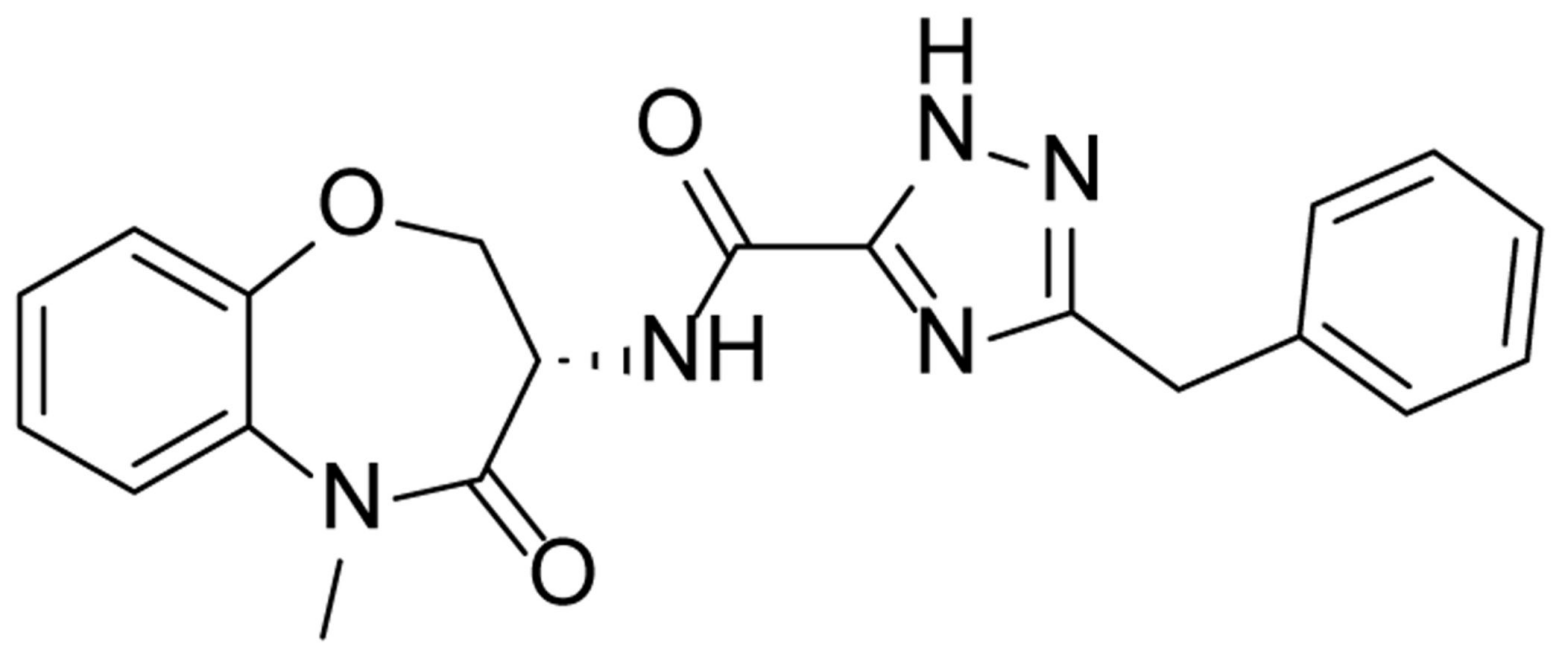
GSK2982772 (12)

At this point, we looked more closely at the 7-membered ring, to understand which elements were required for antiparasitic activity (Table 3). Using compound **1** as a start-point, each of the carbonyls was removed to give **10** and **11** respectively; both changes led to a loss of potency, alongside a small increase in lipophilicity. Replacing the unsubstituted amide with an ether was particularly interesting as it led to a compound with the same core as GSK2982772 (**12**, Fig. 2), a RIPK1 inhibitor which progressed into clinical trials. ^26^ Making this change to **1** gave **13**, which disappointingly had only weak antiparasitic activity.

We were still focused on identifying suitable compounds to progress into the *T. cruzi* washout assay and identified **14** as a potentially interesting analogue (Table 4). Compound **14** was included in the ‘kinetobox’, which was a set of compounds published by GSK, identified from a screen of their compound collection against *T. cruzi* and the related kinetoplastid parasites *Leishmania* and *T. brucei*. ^27^ Compound **14** was of interest due to having the same benzodiazepinedione core as **1**, but with substituents on both the core amides, and without the urea substituent. Pleasingly, compound **14** retained antiparasitic activity, with a half log-unit improvement compared to **1** (pIC_50_ 6.7 compared to 6.0) and much improved aqueous solubility, albeit with an increased Chrom LogD_pH7.4_ of 5.0. To address the chiral centre which was present in all compounds in the series, **14** was separated into its single enantiomers using chiral preparative HPLC and both enantiomers showed similar activity to the parent compound (pIC_50_’s of 6.5 and 6.6, n=2, see supplementary file 1). As such, all other compounds were tested as racemates. It was possible that the ureas could racemise in the assay conditions used, so for any compounds suitable for progression to *in vivo* studies, the single enantiomers would be separated, fully profiled to select the most suitable one, and the potential for racemisation investigated at that point.

To further explore this sub-series, the simplified analogue **15** was screened. This analogue was an intermediate on the synthetic pathway to other analogues in the series and was thus available for screening. Here, the amide N-substituent of **14** was replaced by a 4-methoxybenzyl substituent, and although it was less potent than **14**, the lower Chrom LogD_pH7.4_ of **15** meant that it had an equivalent LLE. We therefore considered it a suitable start-point for further optimisation. Whilst we did explore changes to the 4-methoxybenzyl group (data not shown), these generally led to significant losses in activity compared to **1** and **15**, so attention was focused on the unsubstituted phenyl ring of **15**. Introduction of a 4-methoxy substituent gave **16**, which improved potency and LLE compared to **15**, and also led to an improvement in mouse microsomal clearance (CL_*i*_ = 7.6 ml/min/g). This is still too high for progression into *in vivo* studies, where compounds with intrinsic clearance <1 ml/min/g are targeted as this should translate to low *in vivo* CL_b_ (e.g. <1/3 liver blood flow) and the potential to give good exposure with oral dosing. **16** Also maintained good aqueous solubility, and this proved to be true for all compounds in this sub-series, when compared to the earlier analogues. Replacement of the 4-methoxy with a 4-fluoro substituent to give **17** had limited impact on potency or solubility, whilst introduction of a heteroatom to **16**, to give methoxypyridyl **18**, was also tolerated. Again, we were interested in replacing the aromatic ring completely to improve the overall properties of the series. This led to cyclopropyl analogue **19** and the substituted cyclobutyl **20**, although both compounds were less potent than **16**. The introduction of a fluorine substituent onto the phenyl ring of the bi-cycle was re-examined, leading to **21** and **22**, with a phenyl and 4-methoxyphenyl substitution respectively. Whilst **21** did not show any improvements compared to **15, 22** showed an increase in antiparasitic activity compared to previous compounds, with a pIC_50_ of 7.2. Compound **22** also had an LLE of 3.0, aqueous solubility of 168 μM and mouse microsomal clearance of 2.4 ml/min/g, making it the most promising compound in the series. Finally, to fully explore the core bicycle, **23** was profiled, where the core amino group was removed but this led to a complete loss of parasitic activity.

At this stage, we were keen to examine our options for progression into the *T. cruzi* washout assay, ^12,21^ and decided to move forward with **2, 14** and **16** to give coverage across both chemotypes. In the assay, the infected Vero cells were treated with the relevant compound at 25 x IC_50_ for a total of 16 days (with compound being replaced every four days). After this dosing period, the compound was washed off the infected cells, and the host cell monolayer was monitored for egressed parasites. The first day egressed parasites were detected post washout was noted down as the relapse day. In this assay, posaconazole, which is known to be ineffective in the clinic, relapsed after 10 days and benznidazole, used as the bench-mark clinical compound, relapsed after 31 / 35 days (2 replicates). We were therefore looking for compounds that relapsed at an equivalent time to benznidazole, or later, to be considered worth pursuing further.

In the case of the three compounds tested, **2** relapsed after 10 days, and the two compounds from the sub-series, **14** and **16**, both relapsed after just 6 days. This unfortunately demonstrated that this series is unlikely to give compounds which could deliver cures in *in vivo* efficacy models of chronic Chagas disease, which would be a critical step towards developing a compound which could be progressed to clinical studies. Aside from the washout data, compound **22** looked to have a reasonable profile for further work (good potency and solubility and reasonable microsomal stability), so we looked to further understand the mechanism of action of the non-urea sub-series.

**Fig. 3:**
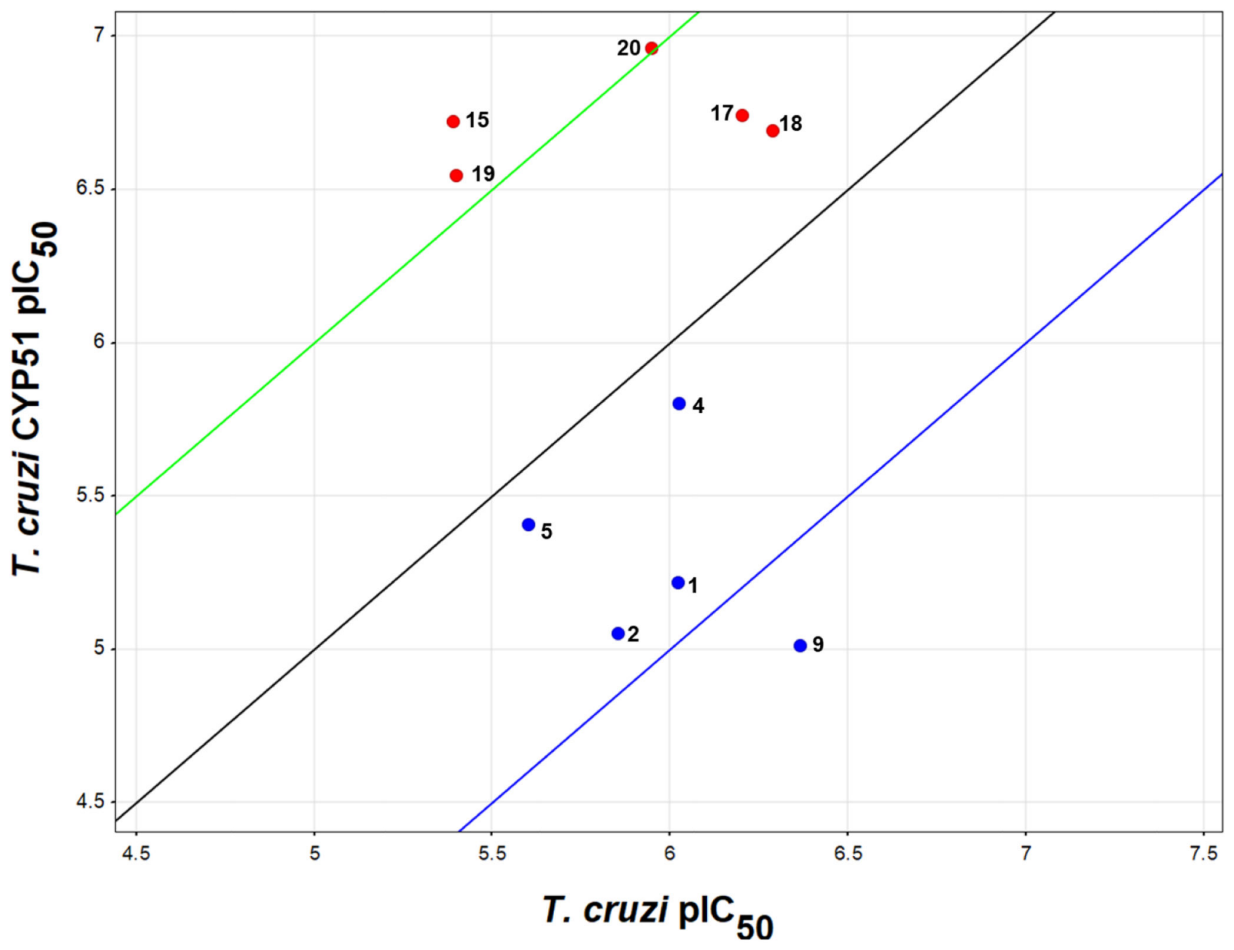
Plot of *T. cruzi* antiparasitic activity vs *Tc*-CYP51 activity: Blue compounds represent the original urea series; red compounds represent the primary amino sub-series. Black line is equipotency line, green line = 10-fold higher potency against CYP51, blue line = 10-fold higher potency against intracellular *T. cruzi* parasites. Dose response curves can be found in supplementary file 1.

A small selection of compounds was run in the *Tc*-CYP51 assay (Fig. 3). This showed that the original urea-based series was 10-fold more potent against the parasites than against *Tc*-CYP51, confirming that this was not the primary mechanism of action (compounds **1, 2, 4, 5** and **9**). However, for the non-urea subseries the *Tc*-CYP51 potency was approximately 10-fold higher than the antiparasitic potency, in line with the cellular activity being driven through *Tc*-CYP51 inhibition (compounds **15, 17 – 20**). This highlights the importance of continual monitoring of the mechanism of action of phenotypic series, especially when substantial structural changes are made.

## Synthesis

**Scheme 1:**
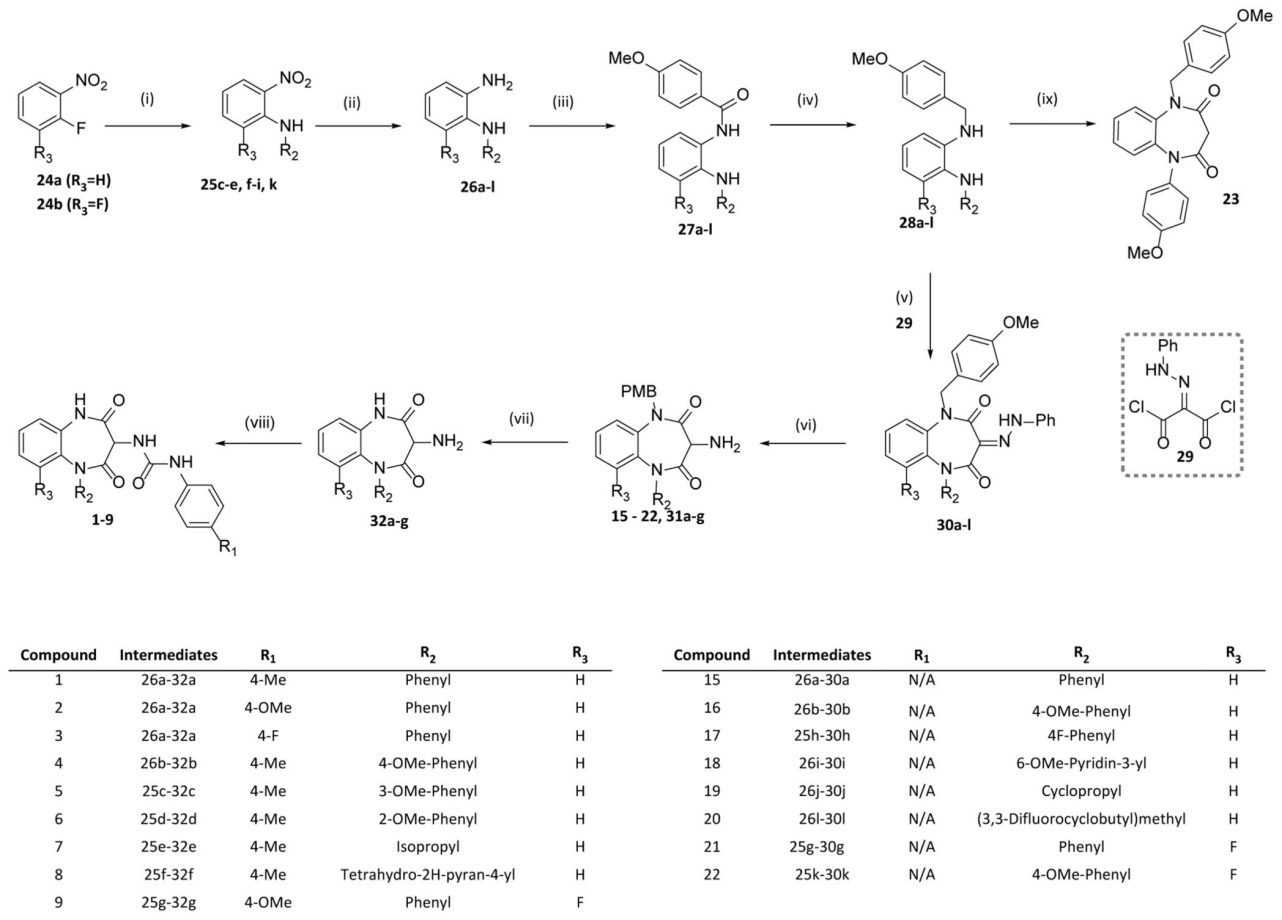
Reagents and Conditions: (i) NaH, RT, DMF (**25c, 25d, 25h**), K_2_CO_3,_ 130°C, DMF (**25f, 25g**) or NEt_3_, 90°C, DMF (**25i**), 16 h, 55-96%; (ii) Fe powder with NH_4_Cl or HCl, EtOH/Water, reflux (**26c, 26d, 26f, 26g, 26k**), or Zn, AcOH (**26h, 26i**), 1 h, 82-93 %; (iii) 4-OMe-benzoyl chloride, NEt_3_, DCM, RT, 2h, 13-96%; (iv) LiAlH_4_, THF, RT, 1.5 h, 19-89%; (v) **29**, THF, RT, 2 h, 8-99%; (vi) Zn, AcOH, 0 - 25°C (**31a-e, 31g, 18, 19, 22**) or 10% Pd-C, H_2_, MeOH (**31f, 17**), 12 – 20 h, 13-90%; (vii) CAN, MeCN, 0 - 25°C, 16 h, 26-79%; (viii) Relevant isocyanate, DCM, RT, 1 – 12 h, 11-87%; (ix) Malonyl Dichloride, THF, 14 h, 16%.

Access to these compounds was achieved via one of two main routes. Synthesis of the benzodiazepinedione scaffold required the diamines **26(a-l)**. Whilst **26(a, b, e, j, l)** were commercially available, the others required synthesis. According to Scheme 1, S_N_Ar displacement of commercially available 2-fluoronitrobenzenes **24(a-b)** with a corresponding aniline in the presence of a base (sodium hydride, potassium carbonate or triethylamine) led to **25(c, d, f-i, k)**. ^28^ The nitro group of the resulting anilines was subsequently reduced with iron and acid (NH_4_Cl or HCl), or via catalytic hydrogenation (10% Pd/C, H_2_, EtOH) to give the required *o*-phenylenediamines**26(c, d, f-i, k)**. With **26(a-l)** in hand, amide formation using 4-methoxybenzoyl chloride to give **27(a-m)** was followed by amide reduction with LiAlH_4_ in THF at room temperature to give diamines **28(a-l)**. ^29^ **28b** Was then reacted directly with malonyl dichloride to yield **23**. ^30^ Alternatively, cyclisation of **28(a-l)** with commercial hydrazone di-acid chloride **29** ^31^ gave intermediates **30(a-l)**. The hydrazone motif of **30(a-l)** was then reduced to the amine using either zinc powder in acetic acid or palladium catalysed hydrogenation / hydrogenolysis to give **31(a-g, 17-19, 22)**, with subsequent ceric ammonium nitrate mediated deprotection of the p-methoxybenzyl (PMB) giving **32(a-g)**. The resulting exo-cyclic amine was finally capped with a relevant isocyanate to yield the desired urea products **1-9**. ^29^

**Scheme 2:**
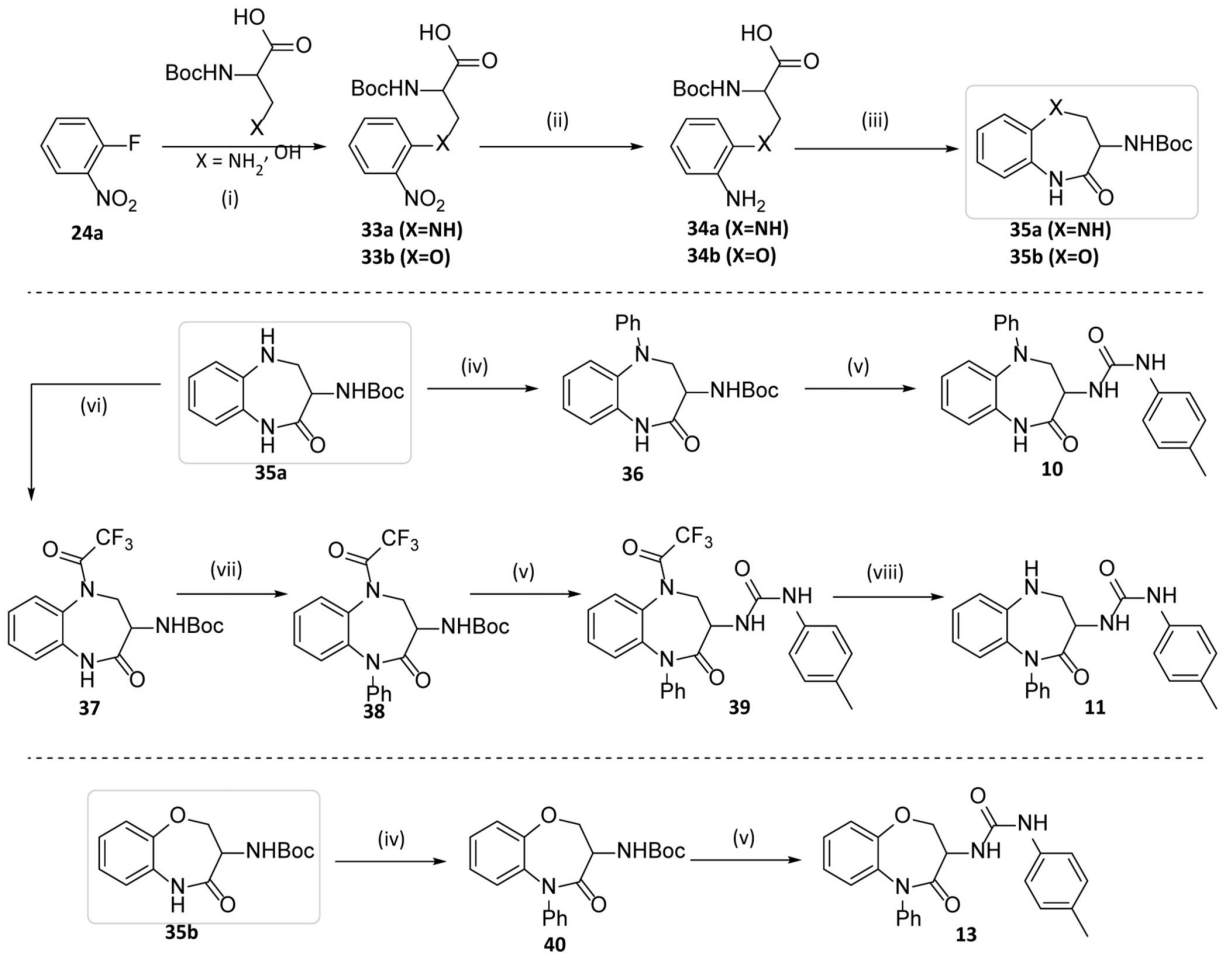
Reagents and Conditions: (i) X=N K_2_CO_3_, EtOH, 90°C, 16 h, 70% (**33a**); X=O NaH, THF, RT, 16 h, 45% (**33b**); (ii) Fe, NH_4_Cl, EtOH/THF/Water, reflux, 1 h, 90%; (iii) HBTU, NEt_3_, DMF, RT, 2 h, 15-26%; (iv) PhBr, Pd_2_dba_3_, Xantphos, 80°C, 16 h, 61-71%; (v) TFA or HCl, DCM, RT, 2 h, 66-68% then 4-methylbenzeneisocyanate, NEt_3_, DCM, RT, 2 h, 25-78%; (vi) TFAA, DIPEA, DCM, RT, 2 h, 72%; (vii) PhI, CuI, K_2_CO_3_, Dioxane, 100°C, 16 h, 66%; (viii) K_2_CO_3_, MeOH, RT, 2 h, 25%.

The second route employed the use of non-symmetrical bi-functional linkers which could be cyclised in multi-step processes to give **10, 11** and **13**, as shown in Scheme 2. Commencing with **24a**, S_N_Ar with the relevant Boc-protected amino acid led to the corresponding functionalised nitrobenzenes **33a** (X=NH) and **33b** (X=O) which were reduced to the anilines **34a-b** using iron / ammonium chloride. Intramolecular cyclisation of these anilino acids using HBTU led to the lactam products **35a** or **35b** (where X = NH or O respectively). Subsequent arylation onto these cores using Buchwald-Hartwig coupling gave the desired aryl analogues **36** and **40**. Finally, BOC deprotection followed by capping with 4-Me-phenylisocyanate gave the desired urea products, **10** and **13**.

Alternatively, protection of the aniline function of **35a** with trifluoroacetyl gave **37** which was then arylated using Ullman coupling conditions. The resulting phenyl analogue **38** was then converted to urea **39** and deprotected to give **11**.

## Conclusions

Through phenotypic screening, compound **1** was identified with antiparasitic activity in a *T. cruzi* intracellular assay. Profiling of **1** showed that its activity was not driven by inhibition of the undesirable target *Tc*-CYP51, nor by inhibition of the parasite electron transport chain, the parasite proteasome or a set of three tRNA-synthetases. Also, activity against trypomastigotes and in a rate-of-kill assay supported the initiation of a hit-to-lead program with an initial aim of identifying suitable compounds for a washout assay. The initial chemistry program identified three compounds with suitable properties (**2, 14** and **16**) and these were taken into the washout assay, where they all gave parasite relapses at similar times to posaconazole, failing to perform as well as benznidazole. This suggested that the mechanism of action of these compounds was not suitable for the development of new treatments for Chagas disease. Further investigation suggested that activity of the primary-amino sub-series was driven by *Tc*-CYP51, explaining its lack of success in the washout assay, although the mechanism-of-action of the initial urea series remained unidentified. This work highlights the importance of monitoring and understanding the mechanism of action of phenotypic compound series being developed for Chagas disease, so that series with a low likelihood of success can be closed early, and resources focused where there is a higher probability of success.

## Experimental

### General

Chemicals and solvents were purchased from Merck, Fluorochem, and Enamine and were used as received. Air- and moisture-sensitive reactions were carried out under an inert atmosphere of nitrogen in oven-dried glassware. Flash column chromatography was performed using pre-packed silica gel cartridges (230–400 mesh, 40–63 μm, from Redisep) using a Teledyne ISCO Combiflash Companion, or Combiflash Retrieve. ^1^H NMR and ^13^C NMR spectra were recorded on a Bruker Avance DPX 500 spectrometer (^1^H at 500.1 MHz, ^13^C at 125.8 MHz). Chemical shifts (δ) are expressed in ppm recorded using the residual solvent as the internal reference in all cases. Signal splitting patterns are described as singlet (s), doublet (d), triplet (t), quartet (q), multiplet (m), broad (br), or a combination thereof. Coupling constants (J) are quoted to the nearest 0.1 Hz. Some peaks in the aromatic region could not be unambiguously assigned as Ar-H or N-H so are left unassigned. High-resolution electrospray measurements were performed on a Bruker Daltonics MicrOTOF mass spectrometer. Low-resolution electrospray (ES) mass spectra were recorded on an Advion Compact mass spectrometer (CMS: model ExpressIon CMS) connected to Dionex Ultimate 3000 UPLC system with diode array detector, or an Acquity UPLC (MS: Waters SQD; ELSD: Waters 2424; Waters PDA; Waters Binary solvent manager; Waters sample manager). HPLC chromatographic separations were conducted using a Waters XBridge C18 column (2.1 mm × 50 mm, 3.5 μm particle size) or Waters XSelect column (2.1 mm × 30 mm, 2.5 μm particle size), eluting with a gradient of 5–95% acetonitrile/water +0.1% ammonia or +0.1% formic acid, or a Waters Acquity BEH C18 column (3 mm × 50 mm, 1.7 μm particle size) eluting with a gradient of 5–95% acetonitrile/water +0.1% formic acid. All intermediates had a measured purity ≥90% and all assay compounds had a measured purity of ≥95% as determined using analytical LC-MS (TIC and UV), unless otherwise stated.

### Synthesis of tested compounds

#### 1-(2,4-dioxo-1-phenyl-2,3,4,5-tetrahydro-1*H*-benzo[*b*][1,4]diazepin-3-yl)-3-(*p-tolyl*)urea (1)

A solution of 3-amino-1-phenyl-1,5-dihydro-2*H*-benzo[*b*][1,4]diazepine-2,4(3*H*)-dione (**32a**) (60 mg, 0.224 mmol), and 1-isocyanato-4-methoxybenzene (0.035 mL, 0.269 mmol) in CH_2_Cl_2_ (3 mL) was stirred under nitrogen at RT for 12 h. The reaction mixture was diluted with water and extracted with EtOAc (3 × 25 mL), dried (Na_2_SO_4_) and concentrated under reduced pressure. The crude material was purified by reverse phase chromatography (Reveleris, biotage snap cartridge, 100 g, eluted with 50-60% formic acid in MeCN). Relevant fractions were combined and evaporated to give **1** (24 mg, 27%) as an off-white solid. ^1^H NMR (500 MHz, DMSO-*d*_*6*_) δ 10.98 (s, 1H, NH), 9.06 (s, 1H, NH), 7.46 (*app*. t, *J* = 7.6 Hz, 2H, ArH), 7.41 – 7.30 (m, 3H, ArH), 7.25 (d, *J* = 8.1 Hz, 2H, ArH), 7.22 – 7.18 (m, 3H, ArH), 7.08 – 7.03 (m, 2H, ArH), 6.94 (d, *J* = 8.2 Hz, 1H, ArH), 6.86 (d, *J* = 7.6 Hz, 1H, NH), 4.98 (d, *J* = 7.5 Hz, 1H, CH), 2.22 (s, 3H, CH_3_). ^13^C NMR (126 MHz, DMSO-*d*_*6*_) δ 166.1, 166.1, 164.8, 164.8, 154.6, 141.6, 138.0, 134.0, 130.6, 129.9, 129.6, 128.5, 128.1, 126.3, 123.3, 118.6, 118.0. HRMS (ES^+^): *m*/*z* [M + H]^+^ calcd for C_23_H_21_N_4_O_3_, 401.1614; found 401.1624. Purity (analytical LC-MS): 90%

#### 1-(2,4-dioxo-1-phenyl-2,3,4,5-tetrahydro-1*H*-benzo[*b*][1,4]diazepin-3-yl)-3-(4-methoxyphenyl)urea (2)

To a solution of 3-amino-1-phenyl-1,5-dihydro-2*H*-benzo[*b*][1,4]diazepine-2,4(3H)-dione (60 mg, 0.224 mmol), in CH_2_Cl_2_ (3 mL) stirred under nitrogen at RT, 1-isocyanato-4-methoxybenzene (0.035 mL, 0.269 mmol) was added. The reaction mixture was stirred at RT for 12 h then diluted with water, extracted with EtOAc (3 × 25 mL), dried (Na_2_SO_4_) and concentrated. The residue was purified using prep HPLC (0.1% Formic Acid:THF:MeCN, x-select C18 (19*250) mm 5 micron, 25 mL/min) to give **2** (15.0 mg, 0.036 mmol, 16 % yield) as an off-white solid. ^1^H NMR (500 MHz, DMSO-*d*_*6*_); δ 10.94 (s, 1H, NH), 8.97 (s, 1H, NH), 7.47 (*app*. t, *J* = 7.8 Hz, 2H, ArH), 7.38 (d, *J* = 7.4 Hz, 1H, ArH), 7.36 – 7.34 (m, 2H, ArH), 7.28 (d, *J* = 9.0 Hz, 2H, ArH), 7.24 – 7.18 (m, 3H, ArH), 6.95 (d, *J* = 8.1 Hz, 1H, ArH) 6.88 - 6.80 (m, 2H, ArH), 6.78 (d, *J* = 7.8 Hz, 1H, NH), 4.99 (d, *J* = 7.6 Hz, 1H, CH), 3.70 (s, 3H, OCH_3_). LRMS (ES ^+^):m/z [MH]^+^ 417. Purity (analytical LC-MS): 98%

#### 1-(2,4-dioxo-1-phenyl-2,3,4,5-tetrahydro-1*H*-benzo[*b*][1,4]diazepin-3-yl)-3-(4-fluorophenyl)urea (3)

To a solution of 3-amino-1-phenyl-1,5-dihydro-2H-benzo[*b*][1,4]diazepine-2,4(3*H*)-dione (60 mg, 0.224 mmol), in CH_2_Cl_2_ (3 mL) stirred under nitrogen at RT, 1-fluoro-4-isocyanatobenzene (0.031 mL, 0.269 mmol) was added. The reaction mixture was stirred at RT for 12 h then diluted with water and extracted with EtOAc (3 × 25 mL), dried (Na_2_SO_4_) and concentrated. The residue was purified using prep HPLC (0.1% Formic Acid:THF:MeCN, x-select C18 (19*250) mm 5 micron, 25.0 mL/min) to give **3** (10 mg, 0.024 mmol, 11 % yield) as an off-white solid. ^1^H NMR (500 MHz, DMSO-*d*_*6*_): δ 10.96 (s, 1H, NH), 9.22 (s, 1H, NH), 7.50 – 7.26 (m, 7H), 7.24 – 7.01 (m, 5H), 6.95 (d, *J* = 7.7 Hz, 1H,), 6.87 (d, *J* = 6.7 Hz, 1H, NH), 4.99 (d, *J* = 7.0 Hz, 1H, CH). ^13^C NMR (126 MHz, DMSO-*d*_*6*_) δ 166.0, 164.7, 154.7, 141.6, 134.0, 131.8, 129.8, 128.4, 128.1, 127.34, 126.3, 126.3, 123.4, 119.6, 119.5, 115.8, 115.6, 55.8, 54.0, 18.5, 17.2. LRMS (ES ^+^):m/z [MH]^+^ 405. Purity (analytical LC-MS): 98%

#### 1-(1-(4-methoxyphenyl)-2,4-dioxo-2,3,4,5-tetrahydro-1*H*-benzo[*b*][1,4]diazepin-3-yl)-3-(*p-tolyl*)urea (4)

To a solution of 3-amino-1-(4-methoxyphenyl)-1,5-dihydro-2*H*-benzo[*b*][1,4]diazepine-2,4(3*H*)-dione (45 mg, 0.151 mmol) in CH_2_Cl_2_ (2 mL) stirred under nitrogen at RT, 1-isocyanato-4-methylbenzene (0.019 mL, 0.151 mmol) was added. The reaction mixture was stirred at RT for 30 min then concentrated and purified using column chromatography (petroleum ether: EtOAc 0 - 100%) to give **4** (25 mg, 0.06 mmol, 37%) as an off-white solid. ^1^H NMR (500 MHz, DMSO-*d*_*6*_); δ 10.91 (s, 1H, NH), 9.04 (s, 1H, NH), 7.32 (d, *J* = 4.0 Hz, 2H, ArH), 7.25 (d, *J* = 8.4 Hz, 2H, ArH), 7.20 (m, 1H, ArH), 7.11 (d, *J* = 8.9 Hz, 2H, ArH), 7.04 (d, *J* = 8.3 Hz, 2H, ArH), 7.00 (d, *J* = 8.9 Hz, 2H, ArH), 6.97 (d, *J* = 8.2 Hz, 1H, ArH), 6.84 (d, *J* = 7.6 Hz, 1H, NH), 4.96 (d, *J* = 7.7 Hz, 1H, CH), 3.79 (s, 3H, OCH_3_), 2.22 (s, 3H, CH_3_). ^13^C NMR (126 MHz, DMSO-*d*_*6*_) δ 166.2, 164.9, 158.8, 154.6, 138.0, 134.3, 131.5, 130.6, 129.6, 129.6, 127.1, 126.2, 123.3, 118.0, 115.0, 55.8, 20.8. HRMS (ES^+^): *m*/*z* [M + H]^+^ calcd for C_24_H_23_N_4_O_4_, 431.1719; found 431.1726. Purity (analytical LC-MS): 95%

#### 1-(1-(3-methoxyphenyl)-2,4-dioxo-2,3,4,5-tetrahydro-1*H*-benzo[*b*][1,4]diazepin-3-yl)-3-(*p-tolyl*)urea (5)

To a solution of 3-amino-1-(3-methoxyphenyl)-1,5-dihydro-2*H*-benzo[*b*][1,4]diazepine-2,4(3*H*)-dione (0.03 g, 0.101 mmol) in CH_2_Cl_2_ (3 mL) stirred under nitrogen at RT, 1-isocyanato-4-methylbenzene (0.013 mL, 0.101 mmol) was added. The reaction mixture was stirred at RT for 30 min then concentrated and purified using column chromatography (petroleum ether: EtOAc 0 - 100%) to give **5**(25 mg, 0.057 mmol, 57 %) as a yellow solid. ^1^H NMR (500 MHz, DMSO-*d*_*6*_): δ 10.91 (s, 1H, NH), 9.04 (s, 1H, NH), 7.39 – 7.32 (m, 3H), 7.27 – 7.19 (m, 3H), 7.07 – 6.99 (m, 3H), 6.97 (dd, *J* = 8.3, 2.3 Hz, 1H, ArH), 6.87 – 6.81 (m, 2H), 6.69 (dd, *J* = 7.8, 1.1 Hz, 1H, ArH), 4.98 (d, *J* = 7.6 Hz, 1H, CH), 3.76 (s, 3H, OCH_3_), 2.22 (s, 3H, CH_3_). ^13^C NMR (126 MHz, DMSO-*d*_*6*_): δ 166.1, 164.7, 160.3, 154.6, 142.6, 137.9, 133.9, 131.7, 130.7, 130.6, 129.6, 127.3, 126.2, 123.3, 120.5, 118.0, 114.5, 113.8. HRMS (ES^+^): *m*/*z* [M + H]^+^ calcd for C_24_H_23_N_4_O_4_, 431.1719; found 431.1702. Purity (analytical LC-MS): 98%

#### 1-(1-(2-methoxyphenyl)-2,4-dioxo-2,3,4,5-tetrahydro-1*H*-benzo[*b*][1,4]diazepin-3-yl)-3-(*p-tolyl*)urea (6)

To a solution of 3-amino-1-(2-methoxyphenyl)-1,5-dihydro-2*H*-benzo[*b*][1,4]diazepine-2,4(3*H*)-dione (230 mg, 0.774 mmol) in CH_2_Cl_2_ (5 mL) stirred under nitrogen at RT, 1-isocyanato-4-methylbenzene (103 mg, 0.774 mmol) was added. The reaction mixture was stirred at RT for 16 h. The reaction was poured into MeOH (5 mL) and CH_2_Cl_2_ (30 mL) and the organic layer washed with water (25 mL), dried (Na_2_SO_4_) and concentrated. The residue was purified using column chromatography (MeOH:CH_2_Cl_2_ 0 - 10%) to give **6** (70 mg, 0.161 mmol, 21 % yield) as a mixture of atropisomers, as white solid. ^1^H NMR (500 MHz, DMSO-*d*_*6*_): δ 10.90 (d, *J* = 15.9 Hz, 1H, NH), 9.04 (d, *J* = 5.6 Hz, 1H, NH), 7.85 – 6.63 (m, 13H), 4.98 (dd, *J* = 10.2, 8.0 Hz, 1H, CH), 3.88, 3.47 (2x s, 3 H, OCH_3_), 2.22 (s, 3H, CH_3_). ^13^C NMR (126 MHz, DMSO-*d*_*6*_): δ 166.2, 165.8, 165.0, 154.9, 154.6, 154.5, 138.0, 133.6, 131.8, 131.4, 131.0, 130.7, 130.6, 130.2 129.6, 129.5, 129.2, 127.0, 126.4, 125.8, 124.9, 123.2, 121.8, 121.0, 118.0, 113.8, 113.2, 56.5, 56.2, 55.4, 20.8. HRMS (ES^+^): *m*/*z* [M + H]^+^ calcd for C_24_H_23_N_4_O_4_, 431.1719; found 431.1726. Purity (analytical LC-MS): 99%

#### 1-(1-isopropyl-2,4-dioxo-2,3,4,5-tetrahydro-1*H*-benzo[*b*][1,4]diazepin-3-yl)-3-(*p-tolyl*)urea (7)

To a solution of 3-amino-1-isopropyl-1,5-dihydro-2*H*-benzo[*b*][1,4]diazepine-2,4(3*H*)-dione (0.080 g, 0.343 mmol) in CH_2_Cl_2_ (1 mL) stirred under nitrogen at RT, 1-isocyanato-4-methylbenzene (0.043 mL, 0.343 mmol) was added. The reaction mixture was stirred at RT for 30 min then concentrated and purified using column chromatography (petroleum ether: EtOAc 0 - 100% then CH_2_Cl_2_:MeOH 0 - 20%) to give **7** (70 mg, 0.190 mmol, 55 %) as an off white solid. ^1^H NMR (500 MHz, DMSO-*d*_*6*_): δ 10.69 (s, 1H, NH), 9.02 (s, 1H, NH), 7.54 (d, *J* = 8.0 Hz, 1H, ArH), 7.41 – 7.31 (m, 2H, ArH), 7.27 (d, *J* = 7.8 Hz, 1H, ArH), 7.22 (d, *J* = 8.0 Hz, 2H, ArH), 7.02 (d, *J* = 8.1 Hz, 2H, ArH), 6.75 (d, *J* = 7.7 Hz, 1H, NH), 4.71 (d, *J* = 7.7 Hz, 1H, CH), 4.45 (sept, *J* = 13.7, 6.8 Hz, 1H, CH(CH_3_)_2_), 2.21 (s, 3H, CH_3_), 1.42 (d, *J* = 6.7 Hz, 3H, CH_3_), 1.15 (d, *J* = 6.9 Hz, 3H, CH_3_). ^13^C NMR (126 MHz, DMSO-*d*_*6*_): δ 166.7, 165.1, 154.6, 138.0, 132.8, 132.6, 130.6, 129.6, 127.8, 126.0, 125.6, 123.2, 118.0, 55.8, 52.8, 22.0, 20.8, 20.3. HRMS (ES^+^): *m*/*z* [M + H]^+^ calcd for C_20_H_23_N_4_O_3_, 367.1770; found 367.1798. Purity (analytical LC-MS): 98%

#### 1-(2,4-dioxo-1-(tetrahydro-2*H*-pyran-4-yl)-2,3,4,5-tetrahydro-1*H*-benzo[*b*][1,4]diazepin-3-yl)-3-(*p-tolyl)*urea (8)

To a solution of 3-amino-1-(tetrahydro-2*H*-pyran-4-yl)-1,5-dihydro-2H-benzo[*b*][1,4]diazepine-2,4(3*H*)-dione (100 mg, 0.363 mmol) in CH_2_Cl_2_ (3 mL) stirred under nitrogen at RT, 1-isocyanato-4-methylbenzene (0.043 mL, 0.343 mmol) was added. The reaction mixture was stirred at RT for 30 min then concentrated and purified using column chromatography (CH_2_Cl_2_:MeOH 0 - 20%) to give **8** (130 mg, 0.317 mmol, 87 %) as an off white solid. ^1^H NMR (500 MHz, DMSO-*d*_*6*_): δ 10.68 (s, 1H, NH), 9.01 (s, 1H, NH), 7.60 (d, *J* = 8.0 Hz, 1H, ArH), 7.43 – 7.34 (m, 2H, ArH), 7.28 (dd, *J* = 7.9, 1.5 Hz, 1H, ArH), 7.22 (d, *J* = 8.4 Hz, 2H, ArH), 7.02 (d, *J* = 8.4 Hz, 2H, ArH), 6.74 (d, *J* = 7.8 Hz, 1H, NH), 4.75 (d, *J* = 7.8 Hz, 1H, CH), 4.25 (tt, *J* = 12.1, 3.8 Hz, 1H, CH), 3.88 (ddd, *J* = 15.7, 11.2, 4.1 Hz, 2H, CH_2_), 3.44 – 3.32 (m, 2H, CH_2_), 2.36 – 2.23 (m, 1H, CH_2_), 2.21 (s, 3H, CH_3_), 2.04 – 1.94 (m, 1H, CH_2_), 1.78 (d, *J* = 11.0 Hz, 1H, CH_2_), 1.48 (d, *J* = 10.9 Hz, 1H, CH_2_). ^13^C NMR (126 MHz, DMSO-*d*_*6*_): δ 166.6, 165.5, 154.6, 138.0, 133.0, 132.8, 130.6, 129.6, 128.0, 126.1, 126.1, 123.2, 118.1, 67.2, 67.1, 58.9, 55.9, 31.6, 30.4, 20.7. HRMS (ES^+^): *m*/*z* [M + H]^+^ calcd for C_22_H_25_N_4_O_4_, 409.1899; 409.1876 found. Purity (analytical LC-MS): 99%

#### 1-(9-fluoro-2,4-dioxo-1-phenyl-2,3,4,5-tetrahydro-1*H*-benzo[*b*][1,4]diazepin-3-yl)-3-(4-methoxyphenyl)urea (9)

To a solution of 3-amino-9-fluoro-1-phenyl-1,5-dihydro-2*H*-benzo[*b*][1,4]diazepine-2,4(3*H*)-dione (80 mg, 0.280 mmol) in CH_2_Cl_2_ (2 mL) stirred under nitrogen at RT, 1-isocyanato-4-methoxybenzene (0.044 mL, 0.337 mmol) was added dropwise. The reaction mixture was stirred at RT for 5 h. The crude reaction was diluted with CH_2_Cl_2_ and filtered. The filtrate was concentrated then purified by prep HPLC (0.1% Formic Acid:THF:MeCN, x-select C18 (19*250) mm 5 micron) to give **9** (60 mg, 0.137 mmol, 49 % yield) as an off white solid. ^1^H NMR (500 MHz, DMSO-*d*_*6*_): δ 11.19 (s, 1H, NH), 8.99 (s, 1H, NH), 7.49 – 7.44 (m, 1H), 7.43 – 7.38 (m, 2H), 7.33 – 7.21 (m, 4H), 7.18 – 7.09 (m, 3H), 6.86 – 6.79 (m, 3H), 5.11 (d, *J* = 7.5 Hz, 1H, CH), 3.70 (s, 3H, OCH_3_). ^13^C NMR (126 MHz, DMSO-*d*_*6*_): δ 166.2, 164.9, 157.0, 155.0, 154.7, 154.6, 140.9, 135.0, 133.6, 129.4, 129.3, 127.6, 126.5, 122.2, 119.6, 118.9, 114.5, 113.7, 113.5, 56.04, 55.6. HRMS (ES^+^): *m*/*z* [M + H]^+^ calcd for C_23_H_20_N_4_O_4_F, 435.1469; 435.1464 found. Purity (analytical LC-MS): 99%

#### 1-(4-oxo-1-phenyl-2,3,4,5-tetrahydro-1*H*-benzo[*b*][1,4]diazepin-3-yl)-3-(*p-tolyl*)urea (10)

To a solution of *tert*-butyl (4-oxo-1-phenyl-2,3,4,5-tetrahydro-1*H*-benzo[*b*][1,4]diazepin-3-yl)carbamate (400 mg, 1.13 mmol) in DCM (3 mL), TFA (0.87 mL, 11.32 mmol) was added at RT. The reaction mixture was stirred under N_2_ at RT for 2h then concentrated under reduced pressure, basified with ammonium hydroxide (10mL) and partitioned between DCM and water. The organic layer was collected, dried over sodium sulfate then concentrated under reduced pressure to give crude 3-amino-5-phenyl-1,3,4,5-tetrahydro-2*H*-benzo[*b*][1,4]diazepin-2-one (200mg), which was used without purification. To a solution of the crude intermediate (100 mg, 0.395 mmol) in CH_2_Cl_2_ (3 mL), 1-isocyanato-4-methylbenzene (0.060 mL, 0.474 mmol) was added and the resulting mixture stirred at RT for 2 h. The mixture was concentrated under reduced pressure and purified by column chromatography (petroleum ether: EtOAc 30 - 60%) to give **10** (120 mg, 0.307 mmol, 78 %) as an off-white solid. ^1^H NMR (500 MHz, DMSO-*d*_*6*_): δ 10.10 (s, 1H, NH), 8.78 (s, 1H, NH), 7.52 – 7.11 (m, 8H), 7.03 (d, *J* = 8.3 Hz, 2H, ArH), 6.82 (*app*. t, *J* = 7.3 Hz, 1H, ArH), 6.70 (d, *J* = 8.1 Hz, 2H, ArH), 6.58 (d, *J* = 7.7 Hz, 1H), 4.53 (dt, *J* = 12.0, 7.0 Hz, 1H, CH), 4.09 (dd, *J* = 10.0, 6.5 Hz, 1H, CH_2_), 3.63 (dd, *J* = 11.8, 10.2 Hz, 1H, CH_2_), 2.22 (s, 3H, CH_3_). ^13^C NMR (126 MHz, DMSO-*d*_*6*_): δ 171.9, 154.9, 148.5, 139.3, 138.0, 136.0, 130.7, 129.6, 129.6, 129.4, 128.4, 126.8, 126.7, 123.9, 119.8, 118.3, 116.6, 58.2, 49.4, 40.5, 40.4, 20.7. HRMS (ES^+^): *m*/*z* [M + H]^+^ calcd for C_23_H_23_N_4_O_2_, 387.1821; 387.1836 found. Purity (analyticalLC-MS): 99%

#### 1-(2-oxo-1-phenyl-2,3,4,5-tetrahydro-1*H*-benzo[*b*][1,4]diazepin-3-yl)-3-(*p-tolyl*)urea (11)

To a solution of *tert-*butyl (2-oxo-1-phenyl-5-(2,2,2-trifluoroacetyl)-2,3,4,5-tetrahydro-1*H*-benzo[*b*][1,4]diazepin-3-yl)carbamate (160 mg, 0.36 mmol) in DCM (3 mL) stirred under nitrogen at 0°C was added 4M HCl (in dioxane) (0.89 mL, 3.56 mmol) dropwise. The reaction mixture was stirred at RT for 2 h then concentrated under reduced pressure and the resulting solid washed with petroleum ether (20 mL) then dried under vacuum to give crude 3-amino-1-phenyl-5-(2,2,2-trifluoroacetyl)-1,3,4,5-tetrahydro-2*H*-benzo[*b*][1,4]diazepin-2-one hydrochloride (150 mg, 0.39 mmol, quant.) as an off-white solid. To a solution of this crude intermediate (150 mg, 0.39 mmol) in DCM (3 mL), 1-isocyanato-4-methylbenzene (0.07 mL, 0.55 mmol) and DIPEA (0.16 mL, 0.92 mmol) were added and the resulting mixture was stirred at RT for 2 hr. The reaction mixture was concentrated under reduced pressure, partitioned against DCM and water, washed with brine, dried over sodium sulphate, then dried under vacuum to obtain crude 1-(2-oxo-1-phenyl-5-(2,2,2-trifluoroacetyl)-2,3,4,5-tetrahydro-1H-benzo[*b*][1,4]diazepin-3-yl)-3-(p-tolyl)urea (120mg, 25%). To a solution of the crude intermediate (100 mg, 0.21 mmol) in MeOH (3 mL), K_2_CO_3_ (86 mg, 0.622 mmol) was added. The resulting mixture was stirred at RT for 2 h then concentrated under reduced pressure and purified by column chromatography (petroleum ether: EtOAc 30 - 60%) to give **11** (20 mg, 0.05 mmol, 25%) as an off-white solid. ^1^H NMR (500 MHz, DMSO-*d*_*6*_): δ 8.73 (s, 1H, NH), 7.40 (*app*. t, *J* = 7.8 Hz, 2H, ArH), 7.35 – 7.26 (m, 3H), 7.24 (d, *J* = 8.4 Hz, 4H, ArH), 7.16 – 7.06 (m, 2H), 7.03 (d, *J* = 8.5 Hz, 1H), 6.88 (ddd, *J* = 8.6, 6.2, 2.6 Hz, 1H, ArH), 6.83 – 6.70 (m, 1H), 6.54 (d, *J* = 7.8 Hz, 1H), 5.43 (d, *J* = 5.2 Hz, 1H, CH), 4.69 (ddd, *J* = 11.6, 7.8, 6.2 Hz, 1H, CH_2_), 3.75 (dt, *J* = 9.5, 5.8 Hz, 1H, CH_2_), 2.22 (s, 3H, CH_3_). ^13^C NMR (126 MHz, DMSO-*d*_*6*_): δ 171.5, 154.8, 142.8, 138.0, 134.2, 130.6, 129.6, 129.5, 128.1, 127.5, 127.3, 126.4, 122.1, 122.1, 118.2, 56.2, 49.7, 20.8. HRMS (ES^+^): *m*/*z* [M + H]^+^ calcd for C_26_H_27_N_4_O_3_, 387.1821; 387.1837 found. Purity (analytical LC-MS): 98%

#### 1-(4-oxo-5-phenyl-2,3,4,5-tetrahydrobenzo[b][1,4]oxazepin-3-yl)-3-(*p-tolyl*)urea (13)

To a solution of 3-amino-5-phenyl-2,3-dihydrobenzo[*b*][1,4]oxazepin-4(5*H*)-one hydrochloride (300 mg, 1.03 mmol) in CH_2_Cl_2_ (3 mL), DIPEA (0.360 mL, 2.06 mmol) and 1-isocyanato-4-methylbenzene (0.16 mL, 1.24 mmol) were added. The resulting mixture was stirred at RT for 2 h then concentrated under reduced pressure and purified by column chromatography (petroleum ether: EtOAc 30 - 60%) to give **13** (100 mg, 0.256 mmol, 25%) as an off-white solid. ^1^H NMR (500 MHz, DMSO-*d*_*6*_): δ 8.79 (s, 1H, NH), 7.45 (*app*. t, *J* = 7.8 Hz, 2H, ArH), 7.37 – 7.28 (m, 3H, ArH), 7.26 – 7.19 (m, 5H, ArH), 7.03 (d, *J* = 8.3 Hz, 2H, ArH), 6.95 – 6.92 (m, 1H, ArH), 6.59 (d, *J* = 7.9 Hz, 1H, NH), 4.86 (dt, *J* = 11.4, 7.7 Hz, 1H, CH), 4.52 (dd, *J* = 9.8, 7.5 Hz, 1H, CH_2_), 4.31 (dd, *J* = 11.3, 10.0 Hz, 1H, CH_2_), 2.22 (s, 3H, CH_3_). ^13^C NMR (126 MHz, DMSO-*d*_*6*_): δ 170.5, 154.8, 150.8, 141.6, 137.8, 136.6, 130.8, 129.7, 129.6, 128.2, 128.2, 127.7, 126.2, 126.2, 123.2, 118.3, 77.9, 77.9. HRMS (ES^+^): *m*/*z* [M + H]^+^ calcd for C_23_H_22_N_3_O_3_, 388.1661; 388.1671 found. Purity (analytical LC-MS): 99%

#### 2-(3-amino-2,4-dioxo-5-phenyl-2,3,4,5-tetrahydro-1*H*-benzo[*b*][1,4]diazepin-1-yl)-*N*-isopropyl-*N*-phenylacetamide (14)

Racemic **14** was synthesised according to the published route. ^32^ The single enantiomers were then separated by chiral preparative HPLC (Chiralpak IA 30 x 150, isocratic method using 50:50 Heptane: Isopropanol) to yield:

Enantiomer 1: ^1^H NMR (500 MHz, DMSO-*d*_*6*_): δ 7.25 – 7.58 (m, 12H, ArH), 7.21 (ddd, *J* = 8.3, 7.11, 1.4 Hz, 1H, ArH), 6.89 (dd, *J* = 8.3, 1.5 Hz, 1H, ArH), 4.82 (sept, *J* = 6.6 Hz, 1H, CH), 4.31 - 4.44 (m, 1 H, CH), 4.11 - 4.23 (m, 2H, CH_2_), 1.97 (br s, 2 H, NH_2_), 1.01 (t, *J* = 7.3 Hz, 6 H, 2x.CH_3_). Purity (analytical LC-MS): 95% Enantiomer 2: ^1^H NMR (500 MHz, DMSO-*d*_*6*_): δ 7.30 - 7.58 (m, 12 H, ArH), 7.21 (*app*. t, *J* = 7.4 Hz, 1 H, ArH), 6.89 (dd, *J* = 8.3, 1.5 Hz, 1H, ArH), 4.82 (sept, *J* = 6.7 Hz, 1 H, CH), 4.28 - 4.48 (m, 1 H, CH), 4.13 - 4.21 (m, 2 H, CH_2_), 1.94 - 2.03 (br s, 2 H, NH_2_), 1.01 (t, *J* = 7.3 Hz, 6 H, 2x.CH_3_). Purity (analytical LC-MS): 95%

#### 3-amino-1-(4-methoxybenzyl)-5-phenyl-1,5-dihydro-2*H*-benzo[*b*][1,4]diazepine-2,4(3*H*)-dione (15)

At 0 °C, under nitrogen, to a solution of (*E*)-1-(4-methoxybenzyl)-5-phenyl-3-(2-phenylhydrazineylidene)-1,5-dihydro-2*H*-benzo[*b*][1,4]diazepine-2,4(3*H*)-dione (150 mg, 0.315 mmol) in acetic acid (1 mL), zinc (169 mg, 2.58 mmol) was added. The resulting mixture was stirred at RT for 12 h then filtered through a Celite bed and washed with EtOAc. The organics were concentrated under reduced pressure and purified by column chromatography (petroleum ether: EtOAc 80 - 100%). Further purification using prep HPLC (THF:MeCN, x-select C18 (19*250) mm 5 micron) yielded **15** (11 mg, 0.028 mmol, 9 %) as an off-white solid. ^1^H NMR (500 MHz, DMSO-*d*_*6*_): δ 7.83 (dd, *J* = 8.3, 1.5 Hz, 1H, ArH), 7.37 (ddd, *J* = 8.4, 7.3, 1.5 Hz, 1H, ArH), 7.27 (dd, *J* = 5.1, 1.9 Hz, 3H, ArH), 7.22 – 7.14 (m, 1H, ArH), 7.04 (d, *J* = 8.7 Hz, 2H, ArH), 6.88 – 6.73 (m, 3H, ArH), 6.64 – 6.48 (m, 2H, ArH), 5.65 (d, *J* = 14.7 Hz, 1H, CH_2_), 4.83 (d, *J* = 14.7 Hz, 1H, CH_2_), 4.23 (s, 1H, CH), 3.73 (s, 3H, OCH_3_), 2.21 (br s, 2H, NH_2_). ^13^C NMR (126 MHz, DMSO-*d*_*6*_): δ 167.6, 167.5, 159.4, 141.3, 136.6, 134.2, 130.0, 129.6, 129.3, 128.1, 127.7, 127.5, 127.3, 126.2, 125.1, 114.5, 56.6, 55.6, 49.6. HRMS (ES^+^): *m*/*z* [M + H]^+^ calcd for C_23_H_22_N_3_O_3_, 388.1661; 388.1666 found. Purity (analytical LC-MS): 97%

#### 3-amino-1-(4-methoxybenzyl)-5-(4-methoxyphenyl)-1,5-dihydro-2*H*-benzo[*b*][1,4]diazepine-2,4(3*H*)-dione (16)

At 0 °C under nitrogen, to a solution of (*E*)-1-(4-methoxybenzyl)-5-(4-methoxyphenyl)-3-(2-phenylhydrazineylidene)-1,5-dihydro-2*H*-benzo[*b*][1,4]diazepine-2,4(3*H*)-dione (1.00 g, 1.97 mmol) in acetic acid (1 mL), zinc (1.03 g, 15.8 mmol) was added. The resulting mixture was stirred at RT for 16 h then filtered through a Celite bed and washed with CH_2_Cl_2_/MeOH 80:20. The organics were concentrated under reduced pressure and redissolved in EtOAc (50 mL) then washed with sat. aq. NaHCO_3_ (4 × 20 mL), water (2 × 20 mL) then brine (2 × 20 mL). The organic layer was separated, dried (Na_2_SO_4_) and concentrated then purified by column chromatography (CH_2_Cl_2_: MeOH 0 - 20%) to give **16** (80 mg, 0.18 mmol, 9 %) as a brown solid. ^1^H NMR (500 MHz, DMSO-*d*_*6*_): δ 7.80 (dd, *J* = 8.4, 1.2 Hz, 1H, ArH), 7.34 (dt, *J* = 7.2, 1.2 Hz, 1H, ArH), 7.19 (dd, *J* = 8.4, 1.2 Hz, 1H, ArH), 7.16 (d, *J* = 1.2 Hz, 2H, ArH), 6.84 – 6.79 (m, 5H, ArH), 6.51 (dd, *J* = 6.8, 2.4 Hz, 2H, ArH), 5.63 (d, *J* = 14.8 Hz, 1H, CH_2_), 4.82 (d, *J* = 14.8 Hz, 1H, CH_2_), 4.18 (s, 1H, CH), 3.75 (s, 3H, OCH_3_), 3.72 (s, 3H, OCH_3_), 2.10 (br s, 2H, NH_2_). LRMS (ES +):m/z [M + H]+ 418.0. Purity (analytical LC-MS): 94%

#### 3-amino-1-(4-fluorophenyl)-5-(4-methoxybenzyl)-1,5-dihydro-2*H*-benzo[*b*][1,4]diazepine-2,4(3*H*)-dione (17)

To a suspension of (*E*)-1-(4-fluorophenyl)-5-(4-methoxybenzyl)-3-(2-phenylhydrazineylidene)-1,5-dihydro-2*H*-benzo[*b*][1,4]diazepine-2,4(3*H*)-dione (3.00 g, 6.07 mmol) in EtOH (30 mL) and EtOAc (30 mL), 10%Pd-C (1.94 g, 1.82 mmol) was added in portions and the resulting mixture stirred under at RT, under 4 atm pressure of hydrogen for 10 h. Further 10% Pd-C (0.646 g, 0.607 mmol) was added to the reaction and stirring continued under a hydrogen atmosphere for a further 16 h. The reaction mixture was filtered through a Celite bed and washed with CH_2_Cl_2_/MeOH 80:20 and the organics concentrated under reduced pressure then purified by column chromatography (CH_2_Cl_2_: MeOH 0 - 20%) to give **17** (700 mg, 1.71 mmol, 28 %) as a brown solid. ^1^H NMR (500 MHz, DMSO-*d*_*6*_): δ 7.81 (d, *J* = 8.3, 1.3 Hz, 1H, ArH), 7.39 – 7.35 (m, 1H, ArH), 7.23 – 7.19 (m, 1H, ArH), 7.12 – 7.07 (m, 2H, ArH), 7.05 – 7.02 (m, 2H, ArH), 6.85 – 6.81 (m, 3H, ArH), 6.65 – 6.61 (m, 2H, ArH), 5.63 (d, *J* = 14.7 Hz, 1H, CH_2_), 4.83 (d, *J* = 14.7 Hz, 1H, CH_2_), 4.23 (s, 1H, CH), 3.58 (s, 3H, OCH_3_), 2.17 (s, 2H, NH_2_). ^13^C NMR (126 MHz, DMSO-*d*_*6*_): δ 167.5, 167.5, 159.4, 141.2, 136.6, 134.2, 130.0, 129.5, 129.3, 128.1, 127.7, 127.5, 127.4, 126.2, 125.1, 114.6, 114.5, 56.6, 55.6, 49.6. HRMS (ES^+^): *m*/*z* [M + H]^+^ calcd for C_23_H_21_N_3_O_3_F, 406.1567; 406.1588 found. Purity (analytical LC-MS): 99%

#### 3-amino-1-(4-methoxybenzyl)-5-(6-methoxypyridin-3-yl)-1,5-dihydro-2*H*-benzo[*b*][1,4]diazepine-2,4(3*H*)-dione (18)

At 0 °C, under nitrogen, to a solution of (*E*)-1-(4-methoxybenzyl)-5-(6-methoxypyridin-3-yl)-3-(2-phenylhydrazineylidene)-1,5-dihydro-2*H*-benzo[*b*][1,4]diazepine-2,4(3*H*)-dione (500 mg, 0.985 mmol) in glacial acetic acid (5 mL) was added zinc dust (515 mg, 7.88 mmol) in portions. The resulting mixture was stirred at RT for 16 h then filtered through a Celite bed and the bed was washed with EtOAc. The filtrate was concentrated under reduced pressure and the residue was dissolved in EtOAc (5 mL), washed with water (20 mL) and brine (20 mL), dried (Na_2_SO_4_), filtered and concentrated. Purification using prep HPLC (THF:MeCN, x-select C18 (20*150) mm 5 micron) gave **18** (55 mg, 0.129 mmol, 13 %) as an off-white solid. ^1^H NMR (500 MHz, DMSO-*d*_*6*_): δ 7.83-7.76 (m, 1H, ArH), 7.48 – 7.31 (m, 2H, ArH), 7.21 (dd, *J* = 11.3, 4.1 Hz, 1H, ArH), 7.09 – 6.95 (m, 2H, ArH), 6.92 – 6.85 (m, 2H, ArH), 6.84 – 6.80 (m, 2H, ArH), 6.72 (d, *J* = 8.8 Hz, 1H, ArH), 5.64 (d, *J* = 14.7 Hz, 1H, CH_2_), 4.83 (t, *J* = 18.3 Hz, 1H, CH_2_), 4.23 (s, 1H, CH), 3.85 (d, *J* = 7.9 Hz, 3H, OCH_3_), 3.73 (s, 3H, OCH_3_), 2.25 (bs, 2H, NH_2_). ^13^C NMR (126 MHz, DMSO-*d*_*6*_): δ 168.1, 167.5, 162.4, 159.5, 145.9, 138.8, 136.4, 134.2, 132.0, 129.9, 129.5, 127.6, 127.5, 126.0, 125.2, 114.6, 110.9, 56.6, 55.6, 53.9, 49.7. HRMS (ES^+^): *m*/*z* [M + H]^+^ calcd for C_23_H_23_N_4_O_4_, 419.1719; 419.1744 found. Purity (analytical LC-MS): 98%

#### 3-amino-1-cyclopropyl-5-(4-methoxybenzyl)-1,5-dihydro-2*H*-benzo[*b*][1,4]diazepine-2,4(3*H*)-dione (19)

At 0 °C under nitrogen, to a solution of 1-cyclopropyl-5-(4-methoxybenzyl)-3-(2-phenylhydrazineylidene)-1,5-dihydro-2*H*-benzo[*b*][1,4]diazepine-2,4(3*H*)-dione (780 mg, 1.77 mmol) in glacial acetic acid (10 mL) was added zinc (926 mg, 14.2 mmol) in portions. The resulting mixture was stirred at RT for 14 h then filtered through a Celite bed and the bed was washed with MeOH (100 mL). The filtrate was concentrated under reduced pressure, adsorbed onto SiO_2_ (3.00 g) and purified by column chromatography (petroleum ether: EtOAc 0 - 100%, then CH_2_Cl_2_: MeOH 0 – 20%) to give **19** (50.0 mg, 0.14 mmol, 7 % yield) as a brown solid. ^1^H NMR (500 MHz, DMSO-*d*_*6*_): δ 7.65 – 7.49 (m, 1H, ArH), 7.33 – 7.23 (m, 1H, ArH), 7.17 – 7.13 (m, 2H, ArH), 6.70 – 6.66 (m, 2H, ArH), 6.58 – 6.55 (m, 2H, ArH), 5.38 (d, *J* = 14.8 Hz, 1H, CH_2_), 4.47 (d, *J* = 14.8 Hz, 1H, CH_2_), 3.79 (s, 1H, CH), 3.50 (s, 3H, OCH_3_), 2.92 (sept., *J* = 3.6 Hz, 1H, CH(CH_2_)_2_), 1.89 (br. s, 2H, NH_2_), 0.82 – 0.65 (m, 1H, CH_2_), 0.38 – 0.20 (m, 1H, CH_2_), 0.00 (q, *J* = 10.1 Hz, 1H, CH_2_), -0.80 – -1.08 (m, 1H, CH_2_). ^13^C NMR (126 MHz, DMSO-*d*_*6*_): δ 169.9, 167.6, 159.1, 137.1, 133.3, 129.4, 127.2, 126.6, 124.6, 124.3, 114.2, 56.6, 55.6, 49.2, 30.1, 11.1, 6.7, 6.6. HRMS (ES^+^): *m*/*z* [M + H]^+^ calcd for C_20_H_22_N_3_O_3_, 352.1661; 352.1687 found. Purity (analytical LC-MS): 95%

#### 3-amino-1-((3,3-difluorocyclobutyl)methyl)-5-(4-methoxybenzyl)-1,5-dihydro-2*H*-benzo[*b*][1,4]diazepine-2,4(3*H*)-dione (20)

At 0 °C, under nitrogen, to a solution of (*Z*)-1-((3,3-difluorocyclobutyl)methyl)-5-(4-methoxybenzyl)-3-(2-phenylhydrazineylidene)-1,5-dihydro-2*H*-benzo[*b*][1,4]diazepine-2,4(3*H*)-dione (0.90 g, 1.78 mmol) in glacial acetic acid (10.0 mL) was added zinc (956 mg, 14.3 mmol) in portions. The resulting mixture was stirred at RT for 12 h then filtered through a Celite bed and the bed washed with EtOAc. The filtrate was concentrated under reduced pressure and purified by prep HPLC (0 - 30% ammonium bicarbonate in water, 30.0 mL/min) to give **20** (450 mg, 1.05 mmol, 59 % yield) as a pale yellow gum. ^1^H NMR (500 MHz, DMSO-*d*_*6*_): δ 7.70 (dd, *J* = 8.0, 1.4 Hz, 1H, ArH), 7.57 (dd, *J* = 7.9, 1.5 Hz, 1H, ArH), 7.42 – 7.33 (m, 2H, ArH), 7.08 (d, *J* = 8.6 Hz, 2H, ArH), 6.82 (d, *J* = 8.6 Hz, 2H, ArH), 5.34 (d, *J* = 15.0 Hz, 1H, CH_2_), 4.90 (d, *J* = 15.0 Hz, 1H, CH_2_), 4.41 – 4.25 (m, 1H, CH_2_), 3.98 (s, 1H, CH), 3.78 – 3.70 (m, 1H, CH_2_), 3.68 (s, 3H, OCH_3_), 2.32 – 1.82 (m, 6H, CH_2,_ NH_2_), 1.65 (m, 1H, CH). ^13^C NMR (126 MHz, DMSO-*d*_*6*_): δ 168.7, 168.4, 168.3, 159.3, 135.0, 134.9, 134.8, 134.6, 129.8, 129.5, 127.6, 127.5, 127.3, 124.6, 124.4, 124.3, 114.3, 56.2, 55.4, 50.9, 49.6, 38.7, 38.7, 38.5, 38.5, 22.8, 22.7, 22.6, 22.5, 22.4, 22.4. HRMS (ES^+^): *m*/*z* [M + H]^+^ calcd for C_22_H_24_N_3_O_3_F_2_, 416.1786; 416.183 found. Purity (analytical LC-MS): 96%

#### 3-amino-6-fluoro-1-(4-methoxybenzyl)-5-phenyl-1,5-dihydro-2*H*-benzo[*b*][1,4]diazepine-2,4(3*H*)-dione (21)

At 0 °C under nitrogen, to a solution of (*E*)-6-fluoro-1-(4-methoxybenzyl)-5-phenyl-3-(2-phenylhydrazineylidene)-1,5-dihydro-2*H*-benzo[*b*][1,4]diazepine-2,4(3*H*)-dione (200 mg, 0.404 mmol) in acetic acid (15 mL), zinc (212 mg, 3.24 mmol) was added. The resulting mixture was stirred at RT for 20 h then filtered through a Celite bed and washed with EtOAc. The organics were concentrated and purified by prep HLC (THF:MeCN, Sunfire C18 (19*250) mm 5 micron) to give **21** (20 mg, 0.046 mmol, 11 %) as a white solid. ^1^H NMR (500 MHz, DMSO-*d*_*6*_): δ 7.75 (d, *J* = 8.5 Hz, 1H, ArH), 7.57 – 7.41 (m, 1H, ArH), 7.22 – 7.10 (m, 4H, ArH), 7.06 (d, *J* = 8.6 Hz, 2H, ArH), 6.81 (d, *J* = 8.6 Hz, 2H, ArH), 6.44 (d, *J* = 7.5 Hz, 2H, ArH), 5.66 (d, *J* = 14.7 Hz, 1H, CH_2_), 4.86 (d, *J* = 14.7 Hz, 1H, CH_2_), 4.37 (s, 1H, CH), 3.72 (s, 3H, OCH_3_), 2.50 (bs, 2H, NH_2_). ^13^C NMR (126 MHz, DMSO-*d*_*6*_): δ 167.7, 167.6, 159.5, 140.7, 137.1, 130.6, 130.6, 130.3, 129.5, 129.0, 129.0, 128.9, 128.6, 127.0, 126.2, 120.8, 120.8, 114.8, 114.6, 56.9, 55.6, 49.4. HRMS (ES^+^): *m*/*z* [M + H]^+^ calcd for C_23_H_21_N_3_O_3_F, 406.1567; 406.1584 found. Purity (analytical LC-MS): 97%

#### 3-amino-6-fluoro-1-(4-methoxybenzyl)-5-(4-methoxyphenyl)-1,5-dihydro-2*H*-benzo[*b*][1,4]diazepine-2,4(3*H*)-dione (22)

To a suspension of (*E*)-6-fluoro-1-(4-methoxybenzyl)-5-(4-methoxyphenyl)-3-(2-phenylhydrazineylidene)-1,5-dihydro-2*H*-benzo[*b*][1,4]diazepine-2,4(3*H*)-dione (100 mg, 0.19 mmol) in acetic acid (2 mL), zinc dust (100 mg, 1.53 mmol) was added. The resulting mixture was stirred at RT for 12 h then filtered through a Celite bed and washed with EtOAc (20 mL). The organics were concentrated and purified by reverse phase chromatography (Isolera, 30 g C18 column, flow rate = 10.0 mL/min, 10-55% MeCN in water (0.1 % ammonium bicarbonate)) to give **22** (30 mg, 0.065 mmol, 34 %) as an off-white solid. ^1^H NMR (500 MHz, DMSO-*d*_*6*_): δ 7.73 (d, *J* = 8.5 Hz, 1H, ArH), 7.50 – 7.44 (m, 1H, ArH), 7.13 (ddd, *J* = 10.6, 8.3, 1.0 Hz, 1H, ArH), 7.06 (d, *J* = 8.6 Hz, 2H, ArH), 6.83 (d, *J* = 8.7 Hz, 2H, ArH), 6.67 (d, *J* = 9.1 Hz, 2H, ArH), 6.33 (d, *J* = 8.9 Hz, 2H, ArH), 5.66 (d, *J* = 14.7 Hz, 1H, CH_2_), 4.85 (d, *J* = 14.6 Hz, 1H, CH_2_), 4.33 (s, 1H, CH), 3.73 (s, 3H, OCH_3_), 3.72 (s, 3H, OCH_3_), 2.05 (s, 2H, NH_2_). ^13^C NMR (126 MHz, DMSO-*d*_*6*_) δ 167.8, 167.7, 159.5, 158.0, 154.9, 136.9, 133.5, 130.4, 130.2, 129.5, 128.8, 128.8, 127.5, 120.7, 114.7, 113.7, 56.8, 55.7, 55.6, 49.4. HRMS (ES^+^): *m*/*z* [M + H]^+^ calcd for C_24_H_23_N_3_O_4_F, 436.1673; 436.1696 found. Purity (analytical LC-MS): 95%

#### 1-(4-methoxybenzyl)-5-(4-methoxyphenyl)-1,5-dihydro-2H-benzo[*b*][1,4]diazepine-2,4(3*H*)-dione (23)

To a stirred solution of *N*1-(4-methoxybenzyl)-*N*2-(4-methoxyphenyl)benzene-1,2-diamine (500 mg, 1.50 mmol) in THF (8 mL) at 0 °C under nitrogen, malonyl dichloride (0.160 mL, 1.65 mmol) in THF (2 mL) was added dropwise. The resulting reaction mixture was stirred at RT for 14 h then concentrated under reduced pressure and the residue purified by column chromatography (pet. ether: EtOAc 0 - 100%) to give a brown gum. Further purification by reverse phase chromatography (Revelris 100 g C18, flow rate = 30 mL/min, 0-100 % MeCN in water (10 mM Ammonium bicarbonate)) yielded **23** (100 mg, 0.246 mmol, 16 % yield) as a white solid. ^1^H NMR (500 MHz, DMSO-*d*_*6*_): δ 7.75 (d, *J* = 8.2 Hz, 1H, ArH), 7.29 (*app*. t, *J* = 7.7 Hz, 1H, ArH), 7.14 (*app*. t, *J* = 7.7 Hz, 1H, ArH), 7.09 – 7.06 (m, 2H, ArH), 6.88 – 6.79 (m, 4H, ArH), 6.79 (d, *J* = 8.1 Hz, 1H, ArH), 6.65 – 6.60 (m, 2H, ArH), 5.60 (d, *J* = 14.9 Hz, 1H, CH_2_), 4.80 (d, *J* = 14.9 Hz, 1H, CH_2_), 3.75 (s, 3H, OCH_3_), 3.73 (s, 3H, OCH_3_), 3.64 (d, *J* = 12.0 Hz, 1H, CH), 3.18 (d, *J* = 12.0 Hz, 1H, CH). ^13^C NMR (126 MHz, DMSO-*d*_*6*_): δ 165.2, 165.0, 159.2, 158.4, 137.7, 134.7, 133.9, 129.7, 129.6, 129.4, 127.0, 126.7, 126.0, 124.9, 114.5, 55.8, 55.6, 48.9, 45.3. HRMS (ES^+^): *m*/*z* [M + H]^+^ calcd for C_24_H_23_N_4_O_4_, 403.1658; 403.1679 found. Purity (analytical LC-MS): 98%

## Supplementary Material

Supplementary File 1

Supplementary Information

## Figures and Tables

**Table 1:** *in vitro* profile of compound **1**. ^a^ *T. cruzi* pIC_50_: potency against intracellular *T. cruzi* amastigotes, data from three independent replicates. ^b^ Vero pIC_50_: potency against host Vero cell. ^c^ Kinetic aqueous solubility measured by CAD (Charged Aerosol Detector) ^24^. ^d^ MLM and HLM Cl_*i*_ refer to mouse and human liver microsomal intrinsic clearance respectively. Scaling factor used is 48.0 mg of microsomal protein/g liver (mouse) and 39.7 mg of microsomal protein/g liver (human) ^25^.^e^ ChromLogD_pH7.4_=CHIpH7.4 ×0.0857–2 where CHI is chromatographic hydrophobicity index, PFI = property forecast index (ChromLogDpH7.4 + no. aromatic rings), LLE = ligand lipophilic efficiency (T. cruzi pIC_50_ - ChromLogD_pH7.4_). ^23^ ^f^ See supplementary Fig. 1. Dose response curves for intracellular *T. cruzi, T. cruzi* trypomastigotes, *T. cruzi* CYP51 and *T. cruzi* proteasome can be found in supplementary file 1.

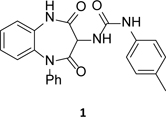	
*T. cruzi* pIC_50_^a^	6.0 ± 0.2
Vero pIC_50_^b^	<4.3
*T. cruzi* trypomastigote pIC_50_	6.6 ± 0.4
*Tc*-CYP51 pIC_50_	4.8 ±0.4
*T. cruzi* oxygen consumption pIC_50_	<4.3
*Tc*-MetRS / *Tc*-LysRS / *Tc*-HisRS pIC_50_	<4.0 / <4.0 / <4.0
Tc-proteasome pIC_50_	4.1
Rate-of-Kill	Cidal activity within 96 hours ^f^
Aqueous solubility μM ^c^	35
MLM CL*_i_* ml/min/g ^d^	0.8
HLM CL*_i_* ml/min/g ^d^	<0.5
Chrom LogD_pH7.4_ / PFI / LLE ^e^	4.6 / 7.6 / 1.4

**Table 2:** *in vitro* profiles of compounds **1** – **9**. See footnotes to Table 1. N ≥ 3 for intracellular potency determinations; dose response curves in supplementary file 1.

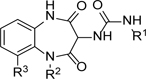									
	R^1^	R^2^	R^3^	*T. cruz/* pIC_50_	Vero pIC_50_	Aqueous solubility μ.M	MLM CL*i* ml/min/g	HLM CL*i* ml/min/g	Chrom Log_DpH7.4_ / PFI / LLE
**1**	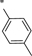	Ph	H	6.0 ± 0.2	<4.3	35	0.8	<0.4	4.6 / 7.6 / 1.4
**2**	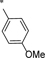	Ph	H	5.9 ± 0.2	<4.3	64	<0.5	<0.4	4.0 / 7.0 / 1.9
**3**	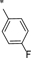	Ph	H	5.0 ± 0.1	<4.3	26	0.6	<0.4	4.4 / 7.4 / 0.6
**4**	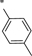	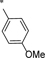	H	6.0 ± 0.3	<4.3	56	1.2	<0.4	4.8 / 7.8 / 1.2
**5**	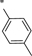	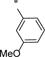	H	5.6 ± 0.1	<4.3	37	1.6	<0.4	4.8 / 7.8 / 0.8
**6**	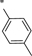	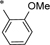	H	4.5 ± 0.1	<4.3	129	2.1	<0.4	4.7 / 7.7 / -0.2
**7**	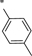	*i*Pr	H	4.9 ± 0.1	<4.3	25	1.8	<0.4	4.2 / 6.2 / 0.7
**8**	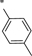	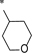	H	4.9 ± 0.1	<4.3	380	0.7	<0.4	3.5 / 5.5 / 1.4
**9**	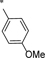	Ph	F	6.4 ± 0.1	<4.3	<1	<0.5	<0.4	4.2 / 7.2 / 2.2

**Table 3:** *in vitro* profiles of compounds **10** – **13**. See footnotes to Table 1. N ≥ 3 for intracellular potency determinations; dose response curves in supplementary file 1.

	Structure	*T. cruzi* pIC_50_	Vero pIC_50_	Aqueous solubility μM	MLM CL_*i*_ ml/min/g	HLM CL_*i*_ ml/min/g	Chrom LogD_pH7.4_ / PFI
**10**	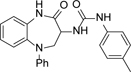	5.1 ± 0.2	4.9 ± 0.3	23	3.5	<0.4	5.7 / 8.7
**11**	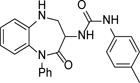	4.6 ± 0.0	<4.3	<1	4.0	<0.4	5.4 / 8.4
**13**	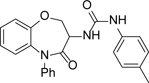	4.6 ± 0.2	4.3	27	4.9	<0.4	6.1 / 9.1

**Table 4:** *in vitro* profiles of compounds **14** – **23**. See footnotes to Table 1. N ≥ 3 for intracellular potency determinations; dose response curves in supplementary file 1.

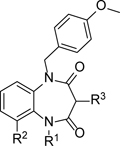									
	R^1^	R^2^	R^3^	*T. cruzi* pIC_50_	Vero pIC_50_	Aqueous solubility μM	MLM CL_*i*_ ml/min/g	HLM CL_*i*_ ml/min/g	Chrom LogD_pH7.4_ / PFI / LLE
**14**	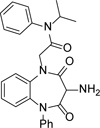			6.7 ± 0.1	<4.3	>500	3.4	<0.4	5.0 / 8.0 / 1.7
**15**	Ph	-H	-NH_2_	5.8 ± 0.1	<4.3	>232	16	1.3	4.1 / 7.1 / 1.7
**16**	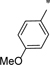	-H	-NH_2_	6.5 ± 0.1	<4.3	>270	7.6	<0.4	4.2 / 7.2 / 2.3
**17**	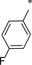	-H	-NH_2_	6.2 ± 0.2	<4.3	>410	10.6	<0.4	4.4 / 7.4 / 1.8
**18**	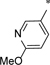	-H	-NH_2_	6.3 ± 0.0	<4.3	>332	5.8	<0.4	3.8 / 6.8 / 2.5
**19**	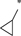	-H	-NH_2_	5.4 ± 0.1	<4.3	>442	11.8	<0.4	3.2 / 5.2 / 2.2
**20**	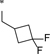	-H	-NH_2_	5.9 ± 0.1	<4.3	>402	12	0.5	4.2 / 6.2 / 1.7
**21**	Ph	-F	-NH_2_	5.9 ± 0.3	<4.3	>217	9.0	<0.4	4.3 / 7.3 / 1.6
**22**	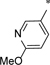	-F	-NH_2_	7.2 ± 0.0	<4.3	168	2.2	<0.4	4.2 / 7.2 / 3.0
**23**	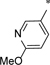	-H	-H	<4.3 ± 0.0	<4.3	175	80	>37	5.1 / 8.1 / -

## Data Availability

The data supporting this article have been included as part of the Supplementary Information. These supplementary files contain synthetic procedures for intermediates, selected ^1^H and ^13^C NMR and HPLC traces of tested compounds, details of reported *in vitro* and DMPK assays, supplementary figure 1, plus replicate data (including dose-response curves) for all reported assay results
