# Supplementary material for: Structure-activity relationships of 1,5-dihydro-2H-benzo[b][1,4]diazepine-2,4(3H)-diones as inhibitors of Trypanosoma cruzi
Source: RSC Med Chem. Author manuscript; Available in PMC 2025 Jun 30. (PMC12208134; doi:10.1039/d5md00185d)
Supplement: Supplementary Information [file EMS206833-supplement-Supplementary_Information.docx]

**Supporting Information**

**Structure-activity relationships of 1,5-dihydro-2*H*-benzo[*b*][1,4]diazepine-2,4(3*H*)-diones as inhibitors of *Trypanosoma cruzi***

**Authors**

Michael G. Thomas, Joanne Dunne, Peter G. Dodd, Emiliana D’Oria, Laura Frame, Adolfo Garcia-Perez, Kate McGonagle, Pilar Manzano, Lorna MacLean, Christy Paterson, Jennifer Riley, John Thomas, Leah S. Torrie, Karolina Wrobel, Kevin D. Read, Maria Marco,* Manu De Rycker*

**Contents**

Synthesis of intermediates S2

^1^H, ^13^C NMR spectra and HPLC traces of final compounds S15

*In vitro* assays S46

Supplementary Figure S49

References S50

**Synthesis**

**General Experimental**

Chemicals and solvents were purchased from Merck, Fluorochem, and Enamine and were used as received. Air- and moisture-sensitive reactions were carried out under an inert atmosphere of nitrogen in oven-dried glassware. Flash column chromatography was performed using pre-packed silica gel cartridges (230–400 mesh, 40–63 μm, from Redisep) using a Teledyne ISCO Combiflash Companion, or Combiflash Retrieve. ^1^H NMR and ^13^C NMR spectra were recorded on a Bruker Avance DPX 500 spectrometer (^1^H at 500.1 MHz, ^13^C at 125.8 MHz). Chemical shifts (δ) are expressed in ppm recorded using the residual solvent as the internal reference in all cases. Signal splitting patterns are described as singlet (s), doublet (d), triplet (t), quartet (q), multiplet (m), broad (br), or a combination thereof. Coupling constants (J) are quoted to the nearest 0.1 Hz. Some peaks in the aromatic region could not be unambiguously assigned as Ar-H or N-H so are left unassigned. High-resolution electrospray measurements were performed on a Bruker Daltonics MicrOTOF mass spectrometer. Low-resolution electrospray (ES) mass spectra were recorded on an Advion Compact mass spectrometer (CMS: model ExpressIon CMS) connected to Dionex Ultimate 3000 UPLC system with diode array detector, or an Acquity UPLC (MS: Waters SQD; ELSD: Waters 2424; Waters PDA; Waters Binary solvent manager; Waters sample manager). HPLC chromatographic separations were conducted using a Waters XBridge C18 column (2.1 mm × 50 mm, 3.5 μm particle size) or Waters XSelect column (2.1 mm × 30 mm, 2.5 μm particle size), eluting with a gradient of 5–95% acetonitrile/water +0.1% ammonia or +0.1% formic acid, or a Waters Acquity BEH C18 column (3 mm × 50 mm, 1.7 μm particle size) eluting with a gradient of 5–95% acetonitrile/water +0.1% formic acid. All intermediates had a measured purity ≥90% and all assay compounds had a measured purity of ≥95% as determined using analytical LC-MS (TIC and UV)

**Abbreviations**

ABC Ammonium bicarbonate

CAN Ceric ammonium nitrate

CH_2_Cl_2_ Dichloromethane

DBU 1,8-Diazabicyclo[5.4.0]undec-7-ene

DIPEA Di*iso*propylethylamine

DMF N,N-Dimethylformamide

DMSO Dimethylsulfoxide

EtOAc Ethyl acetate

EtOH Ethanol

HRMS High resolution mass spectrometry

LRMS Low resolution mass spectrometry

LCMS Liquid chromatography-mass spectrometry

MeCN Acetonitrile

MeOH Methanol

MTBE Methyl tert-butyl ether

Quant. Quantitative

RT Room temperature

TFAA Trifluoroacetic anhydride

TFA Trifluoroacetic acid

THF Tetrahydrofuran

TLC Thin layer chromatography

UPLC Ultra-performance liquid chromatography

**Syntheses of Intermediates**

**3-methoxy-*N*-(2-nitrophenyl)aniline (25c)**

To a solution of 3-methoxyaniline (1.00 g, 8.12 mmol) in DMF (10 mL) stirred under nitrogen at 0 °C was added sodium hydride (60% oil dispersion) (0.487 g, 12.2 mmol) portion-wise. The reaction mixture was stirred at RT for 30 mins, then 1-fluoro-2-nitrobenzene (1.15 g, 8.12 mmol) was added at 0 °C. The reaction mixture was stirred at RT for 16 h. Reaction mixture was quenched with ice-water and extracted with EtOAc (100 mL × 2). Combined EtOAc layers were washed with water (2 × 50 mL), then dried over Na_2_SO_4_, and evaporated under vacuum. **25c** (1.30 g, 86%) obtained as a red gum, which was telescoped into the next step without further analysis or purification.

**LRMS (ES +)**:m/z [M + H]+ 245.1

***N*1-(3-methoxyphenyl)benzene-1,2-diamine (26c)**

To a stirred solution of *N*-(3-methoxyphenyl)-2-nitroaniline (1.10 g, 4.50 mmol) in EtOH (10 mL) and water (10 mL) was added iron (1.26 g, 22.5 mmol) followed by ammonium chloride (1.20 g, 22.5 mmol) under nitrogen at room temperature. The resulting reaction mixture was stirred at 80 °C for 1 hour. The reaction mixture was filtered through Celite, washing with methanol, then dried under vacuum. The residue was partitioned against EtOAc and water, dried over Na_2_SO_4_ and evaporated under vacuum, to give **26c** (900 mg, 63%) crude material as a brown gum, which was used without purification in the next step.

**LRMS (ES^+^)**: m/z [M + H]^+^ 215.0

**4-methoxy-*N*-(2-(phenylamino)phenyl)benzamide** (**27a**)

To a solution of *N*1-phenylbenzene-1,2-diamine (10.0 g, 54.3 mmol), and NEt_3_ (11.4 mL, 81mmol) in CH_2_Cl_2_ (150 mL) stirred under N_2_ at 0°C, was added 4-methoxybenzoyl chloride (8.08 mL, 59.7 mmol) and the resulting reaction mixture was stirred at RT for 2 h. TLC (50% EtOAc in petroleum ether) showed the reaction was complete. The reaction mixture was washed sequentially with water (100 mL), 1M HCl (50 mL and brine (50 mL). It was dried over Na_2_SO_4_ and concentrated under reduced pressure to give **27a** (19.0 g, quant.) as a dark brown solid.

**^1^H NMR (400 MHz, DMSO**-*d_6_***)** δ 9.59 (s, 1H, NH), 7.90 – 7.88 (m, 2H, ArH), 7.60 - 7.58 (m, 1H, ArH), 7.46 (s, 1H, NH), 7.32 - 7.30 (m, 1H, ArH), 7.21 – 7.13 (m, 3H, ArH), 7.04 - 7.00 (m, 3H, ArH), 6.92 – 6.90 (m, 2H, ArH), 6.80 – 6.77 (m, 1H, ArH), 3.83 (s, 3H, OCH_3_).

**LRMS (ES^+^)**: m/z [M + H]^+^ 319.2.

**N1-(4-methoxybenzyl)-N2-phenylbenzene-1,2-diamine** (**28a**)

To a solution of **4-methoxy-*N*-(2-(phenylamino)phenyl)benzamide** (**27a**) (15.0 g, 47.1 mmol) in THF (100 mL) stirred under nitrogen at 0°C was added a solution of LiAlH_4_ (2.0 M, 40.0 mL, 80 mmol) dropwise over 30 min. The reaction mixture was stirred at 60 °C for 90 min. The reaction mixture was cooled to 0 °C and quenched with MeOH (30 mL), filtered through Celite, then Na_2_SO_4_. The filtrate was washed with 10% MeOH and CH_2_Cl_2_ (500 mL) and the filtrate dried (Na_2_SO_4_) and evaporated under vacuum to give **28a** (10.0 g, 58%) as a brown solid.

**^1^H NMR (400 MHz, DMSO**-*d_6_***):** δ 7.27 – 7.25 (m, 2H), 7.15 – 7.11 (m, 2H), 7.03 – 7.01 (m, 1H), 6.91 - 6.86 (m, 3H), 6.72 – 6.86 (m, 3H), 6.72 – 6.66 (m, 2H), 4.24 (s, 2H, CH_2_), 3.74 (s, 3H, OCH_3_).

**LRMS (ES^+^)**: m/z [M + H]^+^ 305

**(*Z/E*)-1-(4-methoxybenzyl)-5-phenyl-3-(2-phenylhydrazineylidene)-1,5-dihydro-2H-benzo[b][1,4]diazepine-2,4(3H)-dione** (**30a**)

A solution of ***N*1-(4-methoxybenzyl)-*N*2-phenylbenzene-1,2-diamine** (**28a**) (2.50 g, 8.21 mmol), in THF (70 mL) and a solution of 2-(2-phenylhydrazineylidene)malonyl dichloride (4.03 g, 16.4 mmol) in THF (70 mL) were added to THF (25 mL) stirred under nitrogen at 0°C simultaneously. The reaction mixture was stirred at RT for 2 h. UPLC_MS showed the desired syn/anti isomeric product. The crude reaction mixture was concentrated, and the residue dissolved in EtOAc (100 mL), washed with water (30 mL), brine (20 mL), then dried over Na_2_SO_4_. Solvent removed under vacuum, and the crude residue chromatographed (Isolera Orochem EZYFLASH, 40 g, Silica 60A 40-63 um), eluted with 40-60%

EtOAc in petroleum ether. The relevant fractions were combined and reduced under vacuum to give **30a** (3.10 g) as a brown solid, as a mixture of geometric isomers.

**LRMS (ES^+^)**: m/z [M + H]^+^ 477.3

**3-amino-1-(4-methoxybenzyl)-5-phenyl-1,5-dihydro-2H-benzo[b][1,4]diazepine-2,4(3H)-dione** (**31a**)

To a solution of **(*E*)-1-(4-methoxybenzyl)-5-phenyl-3-(2-phenylhydrazineylidene)-1,5-dihydro-2H-benzo[b][1,4]diazepine-2,4(3H)-dione** (**30a**) (1.50 g, 3.15 mmol) in acetic acid (30 mL) stirred under nitrogen at 0°C was added zinc (1.69 g, 25.8 mmol). The reaction mixture was stirred at 25°C for 12 h. The reaction mixture was filtered through Celite, washing with EtOAc and concentrated under vacuo. The crude material was dissolved in CH_2_Cl_2_ (20 mL), adsorbed onto silica gel (3 g), and purified by flash column chromatography (Isolera Orochem Ezyflash, 100 g prepack snap cartridge) with 0-10% CH_2_Cl_2_ /MeOH to give **31a** (1.10 g, 2.77 mmol, 88%) as a pale yellow solid.

**^1^H NMR (400 MHz, DMSO**-*d_6_***):** δ 7.84 – 7.82 (m, 1H, ArH), 7.34 – 7.34 (m, 1H, ArH), 7.28 – 7.24 (m, 3H, ArH), 7.21 – 7.17 (m, 1H, ArH), 7.05 – 7.02 (m, 2H, ArH), 6.84 – 6.79 (m, 3H, ArH), 6.58 – 6.55 (m, 2H, ArH), 5.67 – 5.63 (m, 1H, CH_2_), 4.82 (m, 1H, CH_2_), 4.22 (s, 1H, CH), 3.72 (s, 3H, OCH_3_). NH_2_ not visible, maybe under solvent peak.

**LRMS (ES^+^)**: m/z [M + H]^+^ 388.2

**3-amino-1-phenyl-1,5-dihydro-2H-benzo[b][1,4]diazepine-2,4(3H)-dione (32a)**

To a solution of **3-amino-1-(4-methoxybenzyl)-5-phenyl-1,5-dihydro-2H-benzo[b][1,4]diazepine-2,4(3H)-dione** (**31a**) (1.10 g, 2.84 mmol) and CAN (6.23 g, 11.4 mmol) in MeCN (30 mL) and water (10 mL) stirred under nitrogen at 0 °C . The reaction mixture was stirred at 25 °C for 16 h, diluted with water (50 mL) and extracted with EtOAc (2 × 100 mL). The mixture was dried (Na_2_SO_4_) and concentrated under vacuum. The crude yellow liquid was purified by flash column chromatography, (Isolera, Orochem Ezyflash, 100 g prepack snap silica cartridge), eluted in 0-10% MeOH in CH_2_Cl_2_. The combined fractions were evaporated under reduced pressure to give impure product, which was further purified by Grace reverse phase (liquid packed, 120 g C18 column (Buchi Reveleris)) eluted with 50-60 % MeCN in water (10 mM formic acid), to give **32a** (420 mg, 1.33 mmol, 47%) as a yellow solid.

**^1^H NMR (400 MHz, DMSO**-*d_6_***):** δ 11.35 (s, 1H, NH), 8.74 (bs, 2H, NH_2_), 7.53 – 7.49 (m, 2H, ArH), 7.44 – 7.33 (m, 3H, ArH), 7.26 – 7.20 (m, 3H, ArH), 6.97 – 6.96 (m, 1H, CH), 5.12 (bs, 1H, CH).

**LRMS (ES^+^)**: m/z [M + H]^+^ 268

**2-((*tert*-butoxycarbonyl)amino)-3-((2-nitrophenyl)amino)propanoic acid (33a)**

To a solution of 1-fluoro-2-nitrobenzene (3.73 mL, 35.4 mmol) in EtOH (100 mL), 3-amino-2-((*tert*-butoxycarbonyl)amino)propanoic acid (7.96 g, 39.0 mmol) and K_2_CO_3_ (14.7 g, 106 mmol) were added. The reaction mixture was stirred at 90 °C for 16 h. After 16 h, the reaction was concentrated under reduced pressure and redissolved in MTBE (100 mL) then washed with water (2 × 100 mL). The aqueous layer was extracted with MTBE (100 mL) then EtOAc (2 × 100 mL). The combined organic layers were dried (Na_2_SO_4_) and concentrated under reduced pressure. No further purification was carried out and **33a (8.4g, 24.79 mmol, 70 % yield)** was used directly in the subsequent step.

**^1^H NMR (400 MHz, DMSO**-*d_6_***):** δ 12.90 (bs, 1H, CO_2_H), 8.21 (t, *J* = 5.8 Hz, 1H, NH), 8.08 (dd, *J* = 1.3, 8.6 Hz, 1H, NH), 7.57 (*app* t, *J* = 5.2 Hz, 1H, ArH), 7.32 (d, *J* = 7.9 Hz, 1H, ArH), 7.09 (d, *J* = 8.5 Hz, 1H, ArH), 6.74 (*app* t, *J* = 5.2 Hz, 1H, ArH), 4.26 – 4.21 (m, 1H, CH), 3.79 – 3.76 (m, 1H, CH_2_), 3.57 – 3.50 (m, 1H, CH_2_), 1.36 (s, 9H, CH_3_)

**LRMS (ES^+^)**: m/z [M + H]^+^ 325

**3-((2-aminophenyl)amino)-2-((*tert*-butoxycarbonyl)amino)propanoic acid (34a)**

Followed method analogous to **26c**

**LRMS (ES^+^)**: m/z [M + H]^+^ 296

***tert*-butyl (2-oxo-2,3,4,5-tetrahydro-1H-benzo[b][1,4]diazepin-3-yl)carbamate (35a)**

To a stirred solution of 3-((2-aminophenyl)amino)-2-((*tert*-butoxycarbonyl)amino)propanoic acid (9.50 g, 32.2 mmol) in DMF (300 mL), NEt_3_ (13.5 mL, 96.0 mmol) was added followed by 2-(1H-benzo[d][1,2,3]triazol-1-yl)-1,1,3,3-tetramethylisouronium hexafluorophosphate(V) (13.4 g, 35.4 mmol). The reaction was stirred at RT for 2 h. The reaction mixture was concentrated and then water (200 mL) added. The aqueous solution was extracted with EtOAc (2 × 200 mL), and the organics combined and washed with brine (200 mL). The organic layers were dried (Na_2_SO_4_) and concentrated. The crude product was adsorbed onto silica (20 g) and purified using column chromatography (100 g Orochem SNAP, petroleum ether: EtOAc 0 - 50%, 50.0 mL/min). Fractions containing product were combined and concentrated. The residue was further purified by column chromatography (10 g Orochem SNAP, petroleum ether: EtOAc 0 - 50%, 50.0 mL/min). **35a** (1.40 g, 4.95 mmol, 15.4 % yield) was isolated as a yellow solid.

**^1^H NMR (400 MHz, DMSO**-*d_6_***):** δ 9.69 (s, 1H, NHC(O)), 6.94 – 6.83 (m, 4H, ArH), 6.74 (t, *J* = 7.4 Hz, 1H, NH), 5.60 (d, *J* = 5.5 Hz, 1H, NHBoc), 4.17 – 4.12 (m, 1H, CH), 3.50 – 3.46 (m, 1H, CH_2_), 1.37 (s, 9H, CH_3_). CH_2_ signal partially obscured by solvent peak (water present in DMSO-*d6*).

**LRMS (ES^+^)**: m/z [M + H]^+^ 245

***tert*-butyl (4-oxo-1-phenyl-2,3,4,5-tetrahydro-1H-benzo[b][1,4]diazepin-3-yl)carbamate (36)**

A solution of *tert*-butyl(2-oxo-2,3,4,5-tetrahydro-1H-benzo[b][1,4]diazepin-3-yl)carbamate (500 mg, 1.80 mmol), bromobenzene (0.38 mL, 3.61 mmol) and K_2_CO_3_ (1.25 g, 9.01 mmol) in *tert*-butanol (6 mL) was degassed and stirred under nitrogen at RT for 15 min. Subsequently, XPhos (172 mg, 0.36 mmol) and Pd_2_(dba)_3_ (330 mg, 0.36 mmol) were added. The resulting reaction mixture was stirred at 80 °C for 16 h. The reaction mixture was concentrated under reduced pressure then water (20 mL) was added. The aqueous solution was extracted with EtOAc (2 × 20 mL), then the combined extracts washed with brine (20 mL) and dried (Na_2_SO_4_). The residue was adsorbed onto silica (3 g) and purified using column chromatography (12.0 g Orochem SNAP, petroleum ether: EtOAc 0 - 50%, 50.0 mL/min). **36** was isolated as white solid (400mg, 1.11 mmol, 62% yield).

**^1^H NMR (400 MHz, DMSO**-*d_6_***):** δ 9.91 (s, 1H, NH), 7.29 – 7.10 (m, 7H), 6.82 (t, *J* = 7.3 Hz, 1H, ArH), 6.64 (d, *J* = 7.8 Hz, 2H, ArH), 4.26 – 4.22 (m, 1H, CH), 3.88 – 3.80 (m, 2H, CH_2_), 1.37 (s, 9H, CH_3_)

**LRMS (ES^+^)**: m/z [M + H]^+^ [M ‒55] 298

***tert*-butyl-(4-oxo-1-(2,2,2-trifluoroacetyl)-2,3,4,5-tetrahydro-1H-benzo[b][1,4]diazepin-3-yl)carbamate (37)**

To a solution of *tert*-butyl(2-oxo-2,3,4,5-tetrahydro-1H-benzo[b][1,4]diazepin-3-yl)carbamate (580 mg, 2.09 mmol) in CH_2_Cl_2_ (2 mL) stirred under nitrogen at RT, TFAA (0.44 mL, 3.14 mmol) and DIPEA (0.73 mL, 4.18 mmol) were added . The reaction mixture was stirred at RT for 2 h then concentrated. The crude product was adsorbed onto silica (2.00 g) and purified using column chromatography (10.0 g Orochem SNAP, petroleum ether: EtOAc 0 - 50%, 18.0 mL/min) to give **37 (570 mg, 1.512 mmol, 72.3 % yield)** as a white solid.

**^1^H NMR (400 MHz, DMSO**-*d_6_***):** δ 10.19 (s, 1H, NH), 7.56 – 7.51 (m, 2H), 7.40 (d, *J* = 8.8 Hz, 1H), 7.33 (*app* t, *J* = 3.3 Hz, 1H), 7.23 (d, *J* = 4.0 Hz, 1H), 4.62 (m, 1H, CH), 4.26 – 4.19 (m, 1H, CH_2_), 3.82 (dd, *J* = 12.4, 7.5 Hz, 1H, CH_2_), 1.35 (s, 9H, CH_3_)

**LRMS (ES^+^)**: m/z [M + H]^+^ 372

***tert*-butyl-(2-oxo-1-phenyl-5-(2,2,2-trifluoroacetyl)-2,3,4,5-tetrahydro-1H-benzo[b][1,4]diazepin-3-yl)carbamate (38)**

To a solution of *tert*-butyl(4-oxo-1-(2,2,2-trifluoroacetyl)-2,3,4,5-tetrahydro-1H-benzo[b][1,4]diazepin-3-yl)carbamate (570 mg, 1.53 mmol) in 1,4-dioxane (2 mL), iodobenzene (0.51 mL, 4.58 mmol) and K_2_CO_3_ (633 mg, 4.58 mmol) were added and degassed under nitrogen at RT for 15 min. Cu(I)I (58.2 mg, 0.31 mmol) and (1*R*,2*R*)-cyclohexane-1,2-diamine (34.9 mg, 0.305 mmol) were added. The resulting reaction mixture was stirred at 100 °C for 16 h. The reaction mixture was concentrated under reduced pressure then water (50 mL) was added. The aqueous solution was extracted with EtOAc (2 × 50 mL), then the combined extracts washed with brine (20 mL) and dried (Na_2_SO_4_). The residue was adsorbed onto silica (2.00 g) and purified using column chromatography (12.0 g Orochem SNAP, petroleum ether: EtOAc 0 - 50%, 18.0 mL/min). **38** (470 mg, 1.01 mmol, 66.4 % yield) was isolated as a white solid.

**^1^H NMR (400 MHz, DMSO**-*d_6_***):** δ 7.65 (d, *J* = 7.8 Hz, 1H), 7.58 (d, *J* = 8.3 Hz, 1H), 7.55 – 7.42 (m, 4H), 7.36 (*app* t, *J* = 7.4 Hz, 1H), 7.13 (d, *J* = 7.4 Hz, 2H), 7.06 (dd, *J* = 8.0, 1.2 Hz, 1H), 4.61 (m, 1H, CH), 4.44 – 4.40 (m, 1H, CH_2_), 3.87 (dd, *J* = 12.2, 7.0 Hz, 1H, CH_2_), 1.38 (s, 9H, CH_3_)

**LRMS (ES^+^)**: m/z [M + H]^+^ [M ‒55]^+^ 394

***tert*-butyl-(4-oxo-5-phenyl-2,3,4,5-tetrahydrobenzo[b][1,4]oxazepin-3-yl)carbamate (40)**

To a solution of tert-butyl (4-oxo-2,3,4,5-tetrahydrobenzo[b][1,4]oxazepin-3-yl)carbamate (1g, 3.59 mmol) in 1,4-Dioxane (2 mL), Iodobenzene (1.20 mL, 10.78 mmol), K_2_CO_3_ (1.49 g, 10.78 mmol) were added. Mixture was stirred and degassed under nitrogen at room temp for 15mins. Then, copper(I) iodide (0.14 g, 0.72 mmol), (1R,2R)-cyclohexane-1,2-diamine (0.07 mL, 0.72 mmol) were added. The resulting reaction mixture was stirred at 100 °C for 16 hr. The reaction mixture was concentrated under reduced pressure to remove solvent and diluted with water (50 ml) extracted with EtOAc (2 x 50 ml), washed with brine (20 ml), dried over Na_2_SO_4_. After filtration, the organic layer was concentrated in vacuo. Residue was dissolved in DCM (4 mL), adsorbed on silica gel ( 10g) and purified by automated column chromatography (Isolera) (25g Orochem snap) eluting with 0-50% ethyl acetate in pet-ether. Collected fractions were combined, evaporated under vacuo to give **40** (1 g, 2.54 mmol, 71 % yield) as an off-white solid.

**^1^H NMR (400 MHz, DMSO**-*d_6_***):** δ 7.45 – 7.41 (m, 2H), 7.32 – 7.27 (m, 4H), 7.22 – 7.14 (m, 3H), 6.94 (d, *J* = 8.0 Hz, 1H), 4.58 – 4.56 (m, 1H, CH), 4.40 – 4.38 (m, 2H, CH_2_), 1.38 (s, 9H, CH_3_)

**LRMS (ES^+^)**: m/z [M + H]^+^ 355

**^1^H, ^13^C NMR spectra of final compounds**

**^1^H NMR spectrum (500 MHz, DMSO-*d_6_*) and ^13^C NMR spectrum (126 MHz, DMSO-*d_6_*)**  **of compound 1**


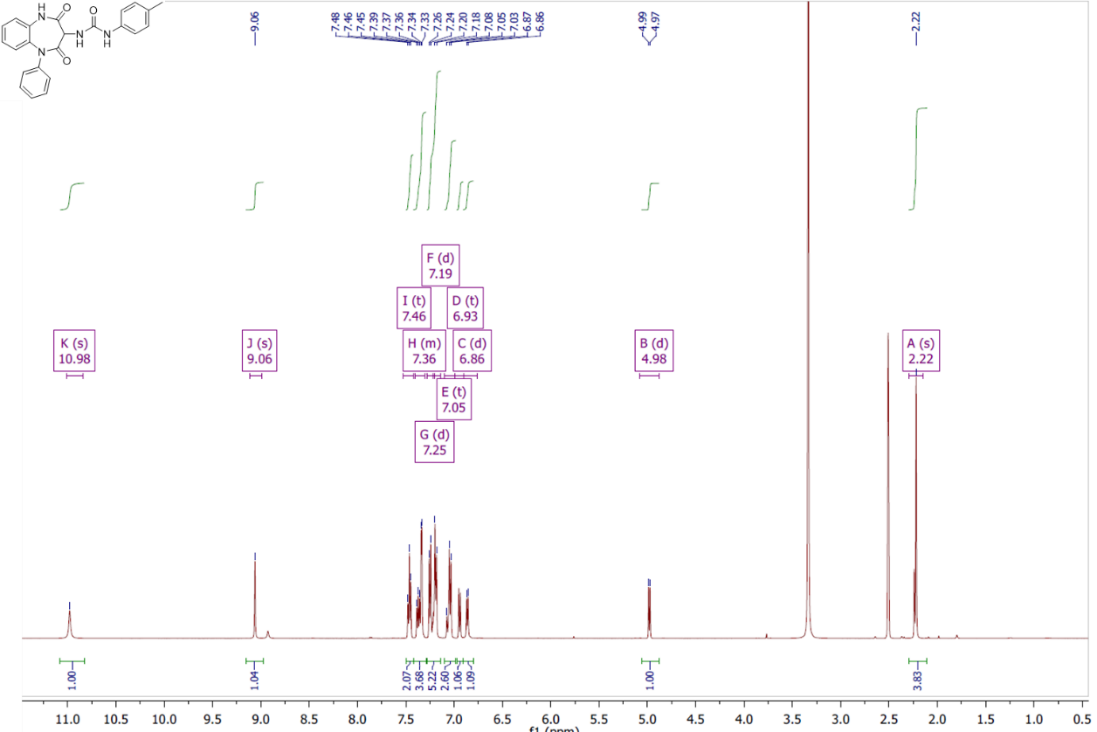


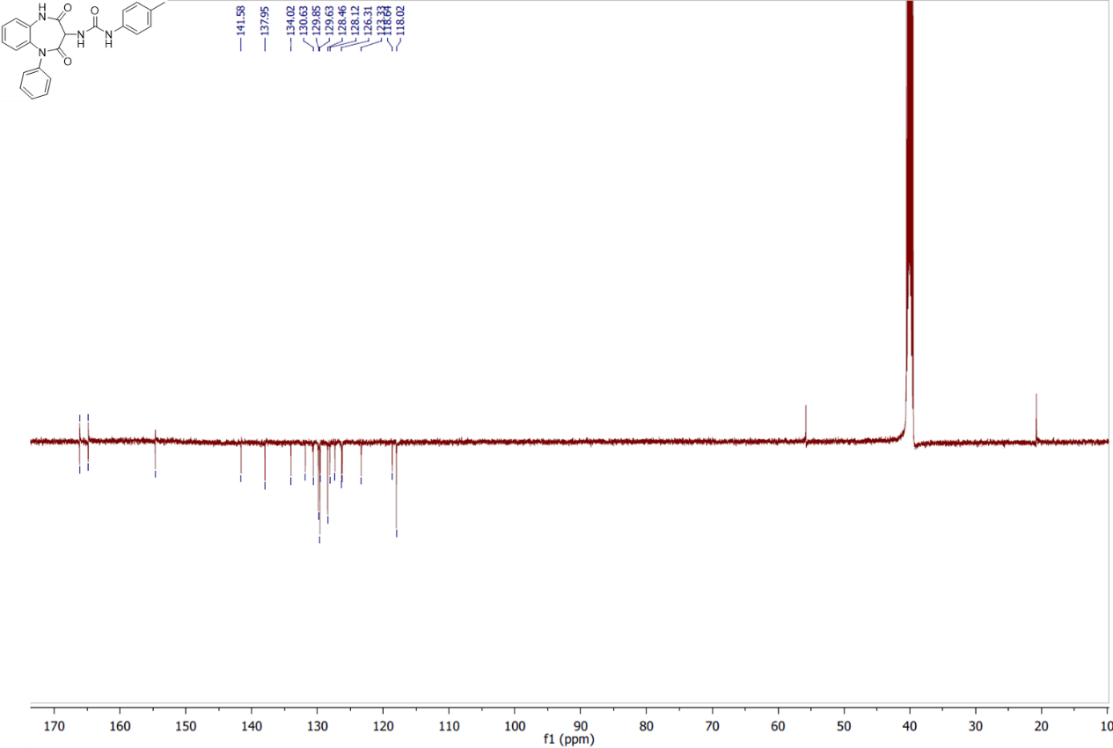
**^1^H NMR spectrum (500 MHz, DMSO-*d_6_*)of compound 2**

**
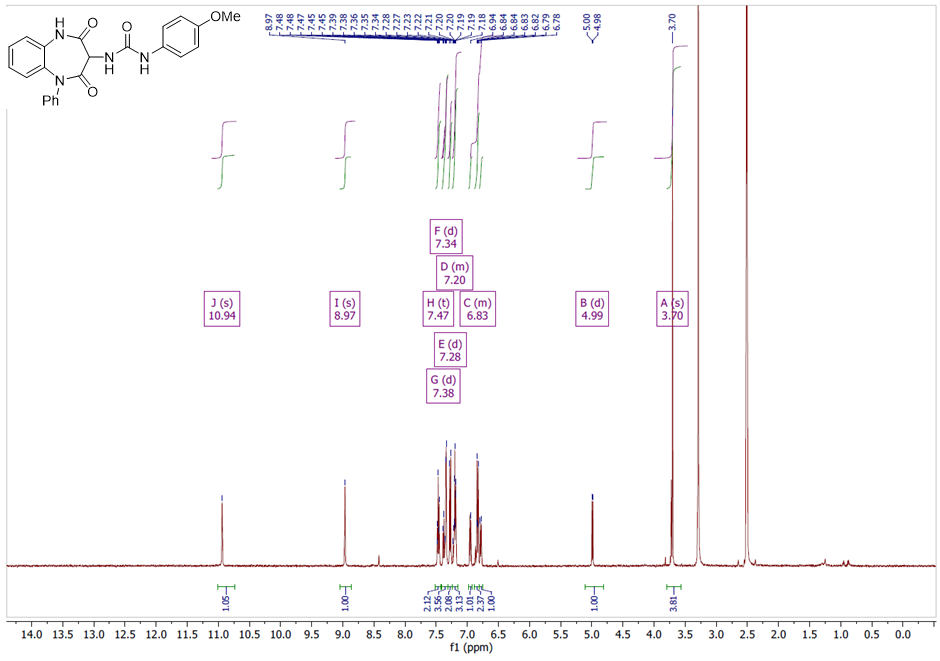
**

**^1^H NMR spectrum (500 MHz, DMSO-*d_6_*) and ^13^C NMR spectrum (126 MHz, DMSO-*d_6_*) of compound 3**

**
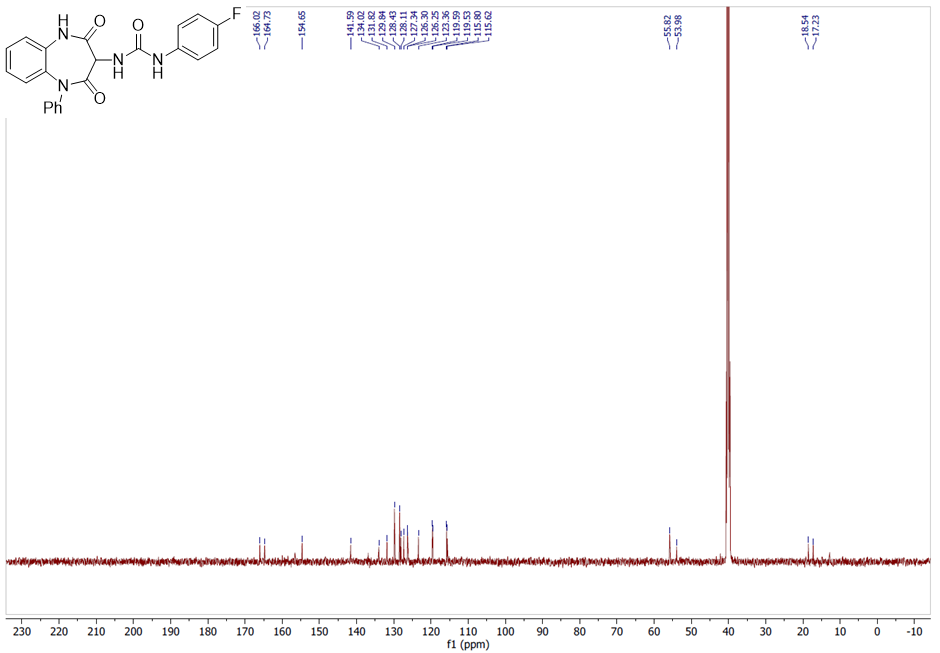

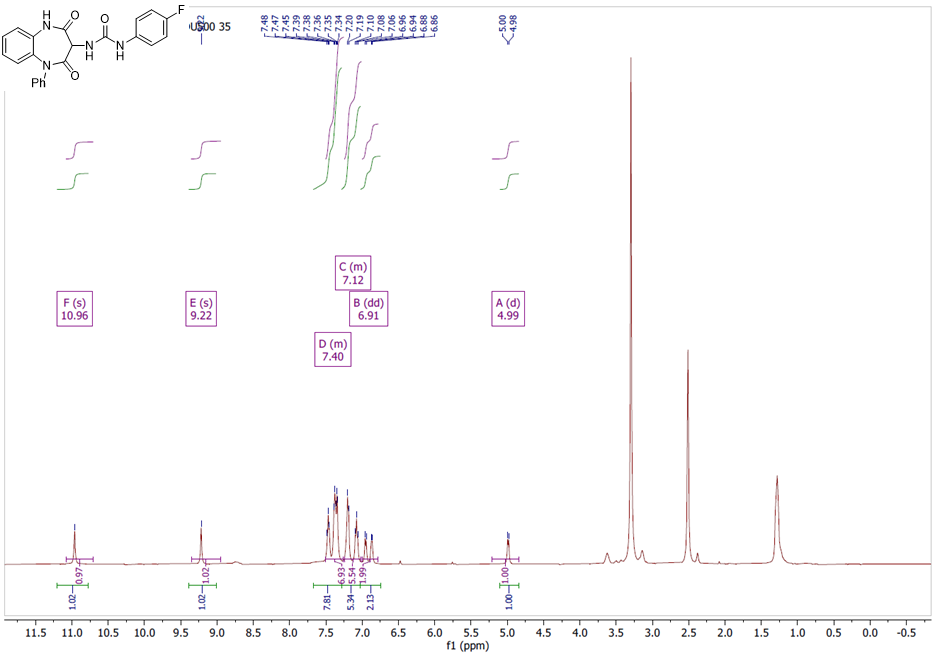
**

**^1^H NMR spectrum (500 MHz, DMSO-*d_6_*), ^13^C NMR spectrum (126 MHz, DMSO-*d_6_*) and HPLC trace of compound 4**

**
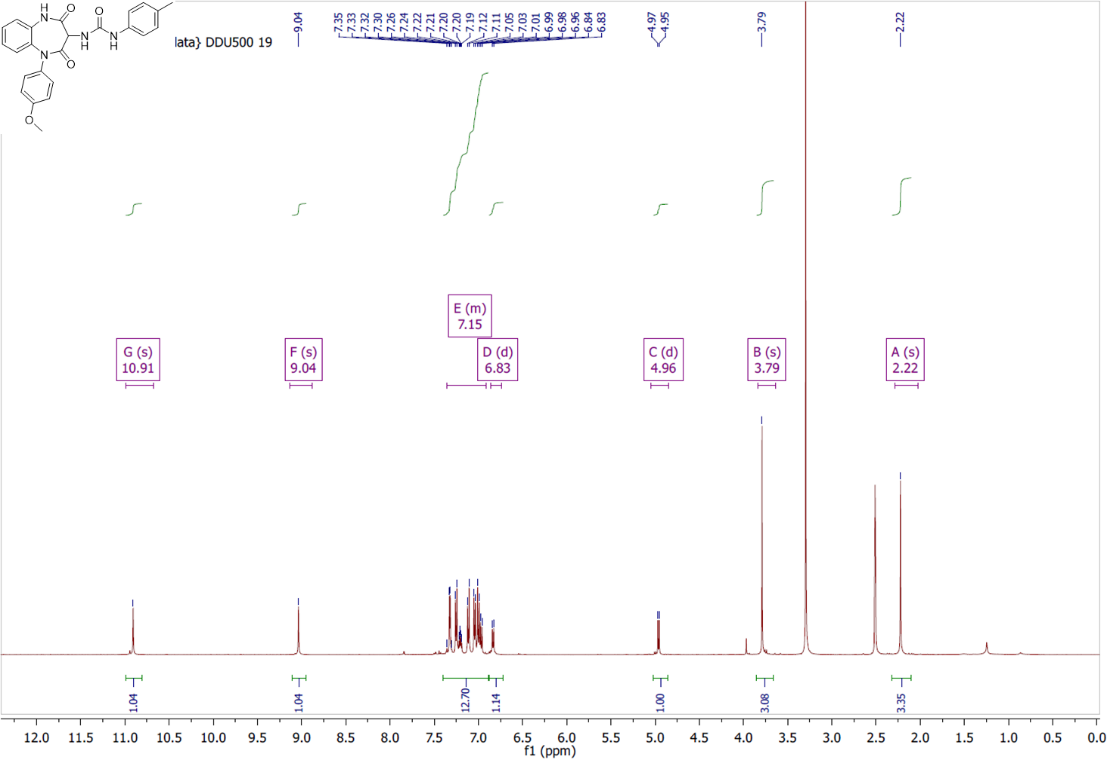

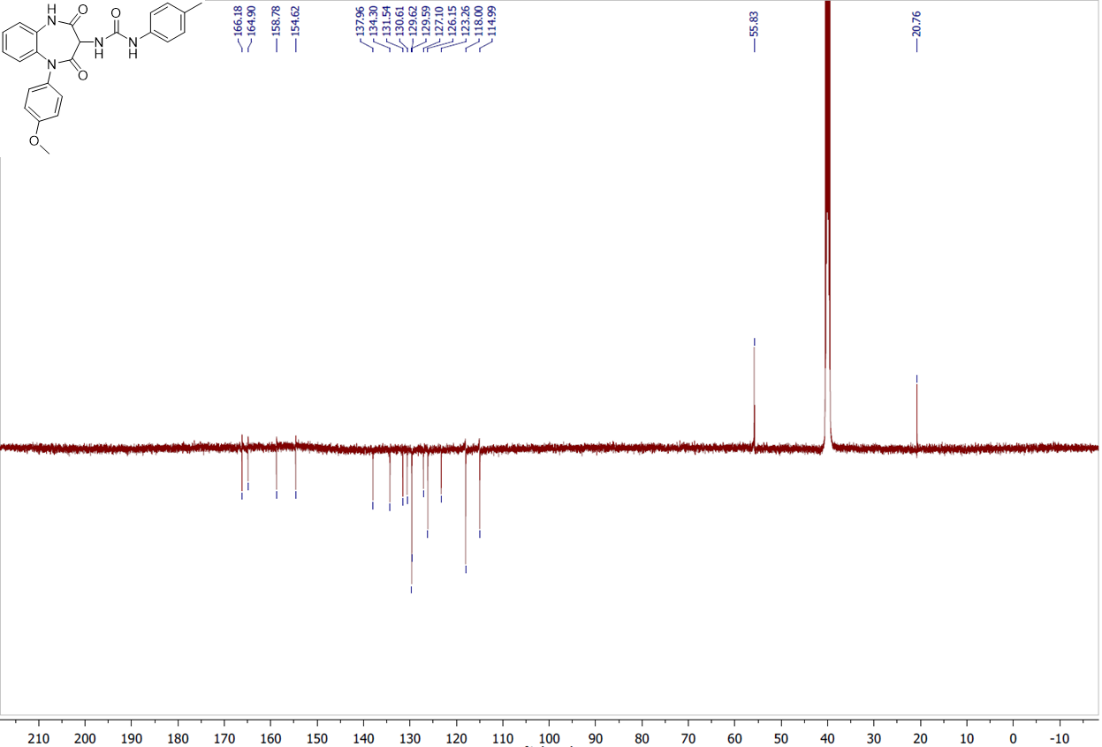
**

**^
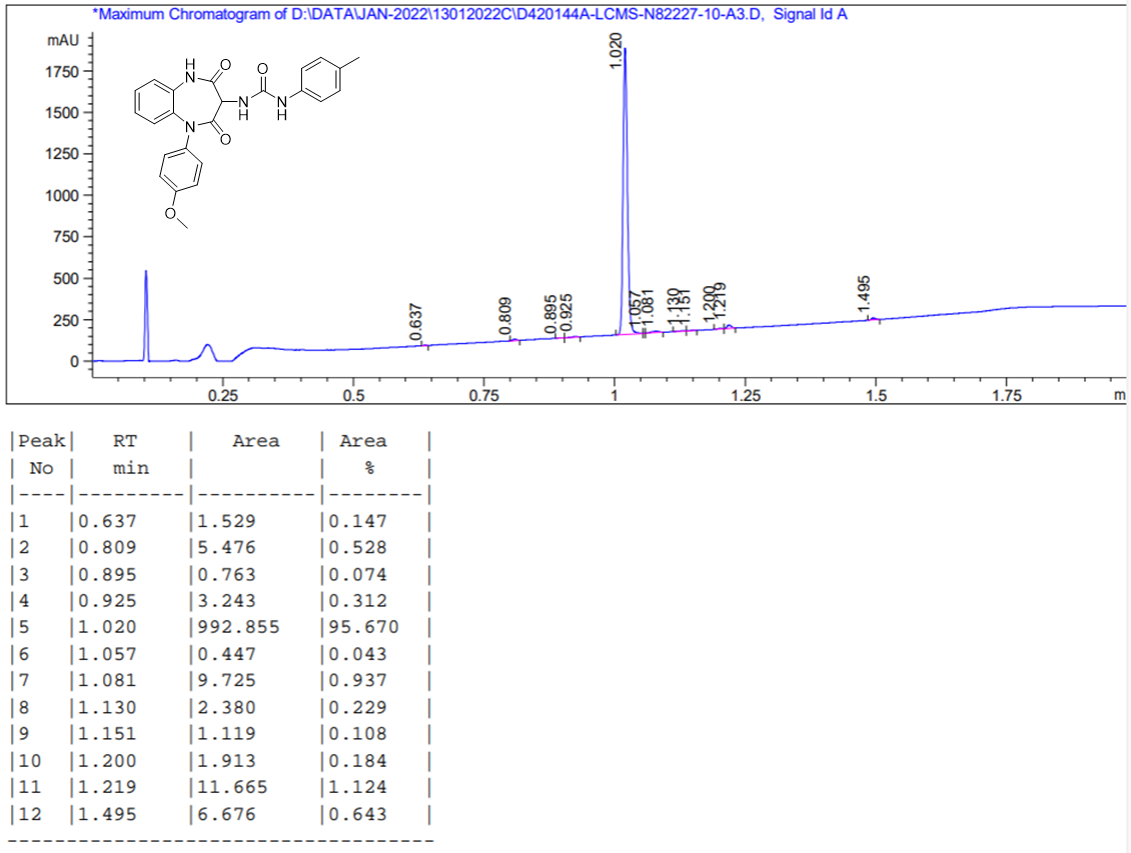
^**

**^1^H NMR spectrum (500 MHz, DMSO-*d_6_*), ^13^C NMR spectrum (126 MHz, DMSO-*d_6_*) and LCMS trace of compound 5**

**
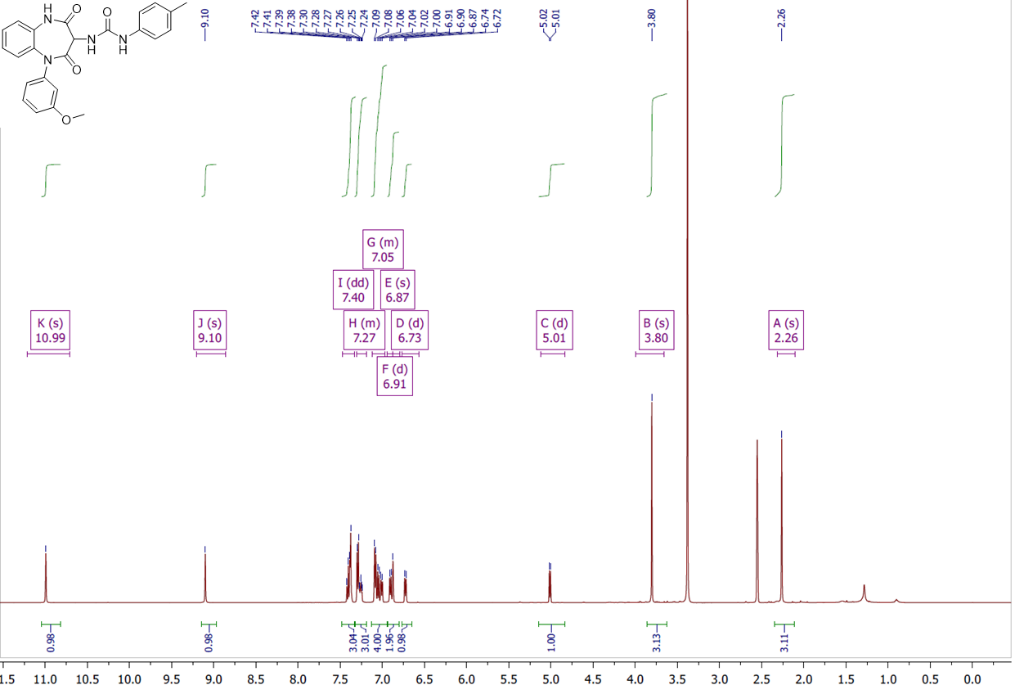
**


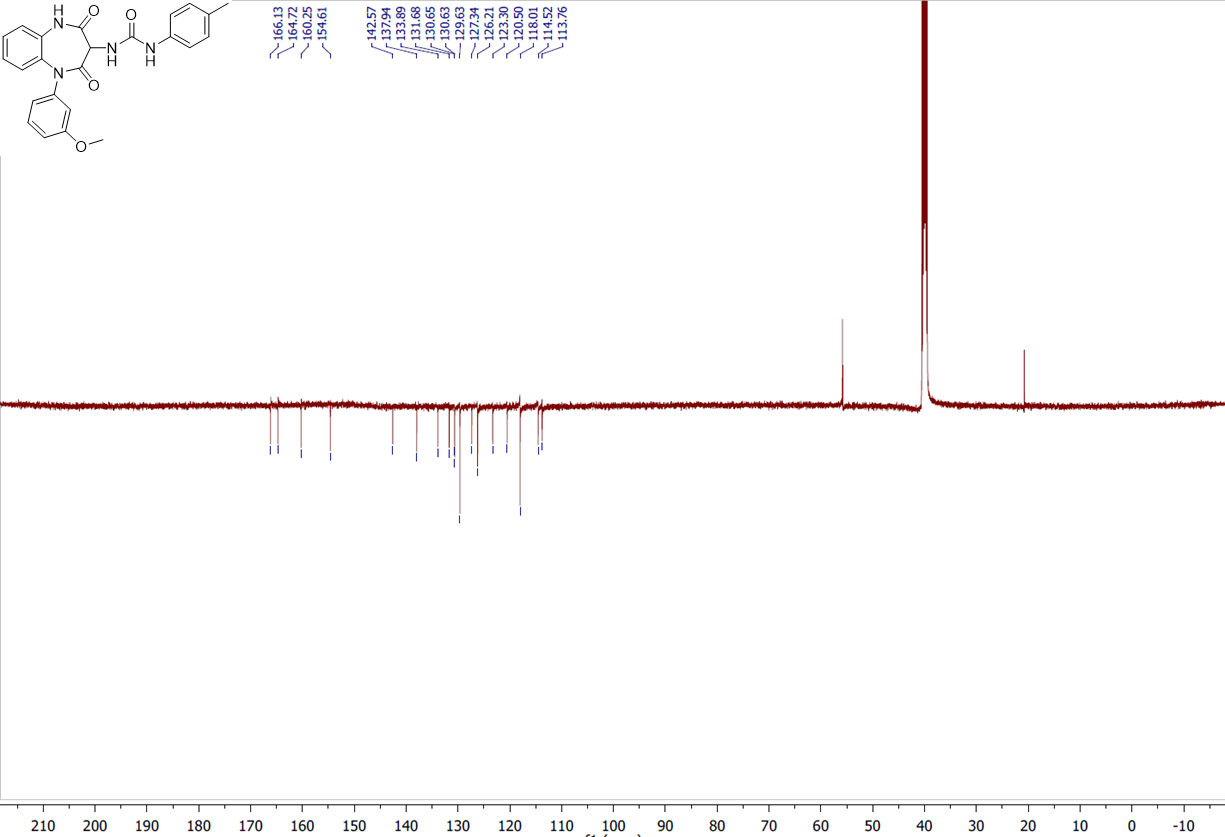


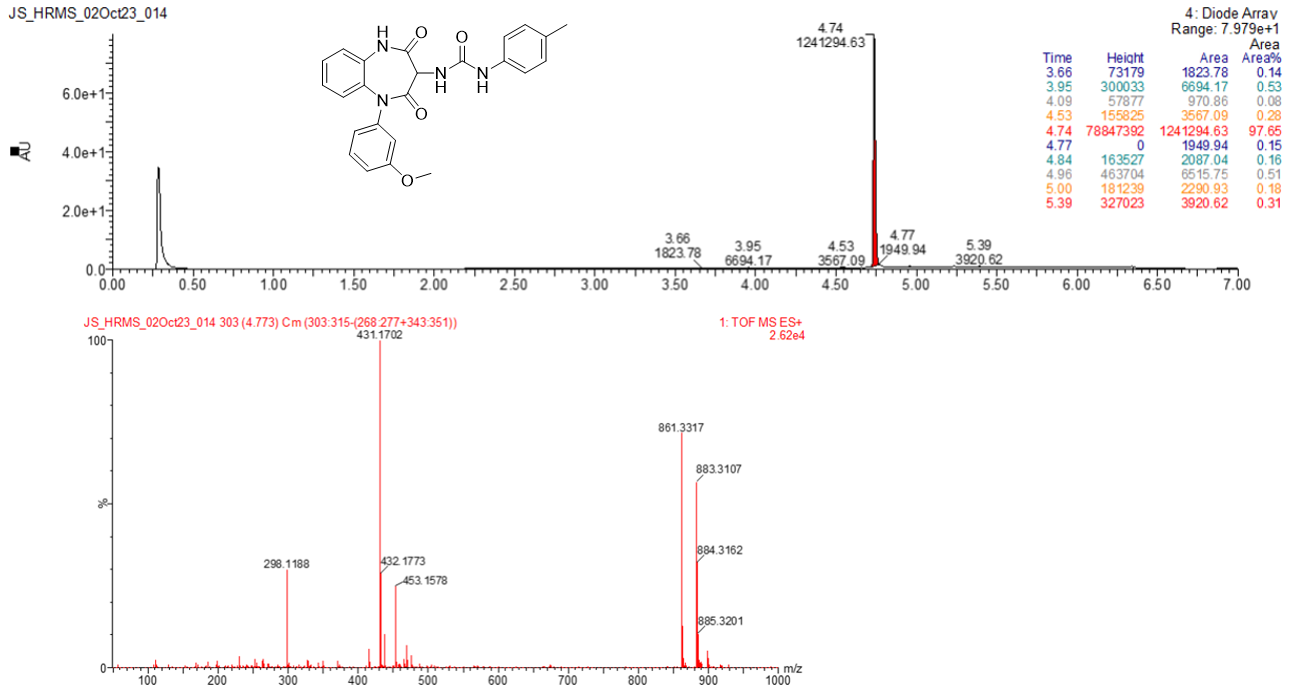


**^1^H NMR spectrum (500 MHz, DMSO-*d_6_*), ^13^C NMR spectrum (126 MHz, DMSO-*d_6_*) and LCMS trace of compound 6**

**
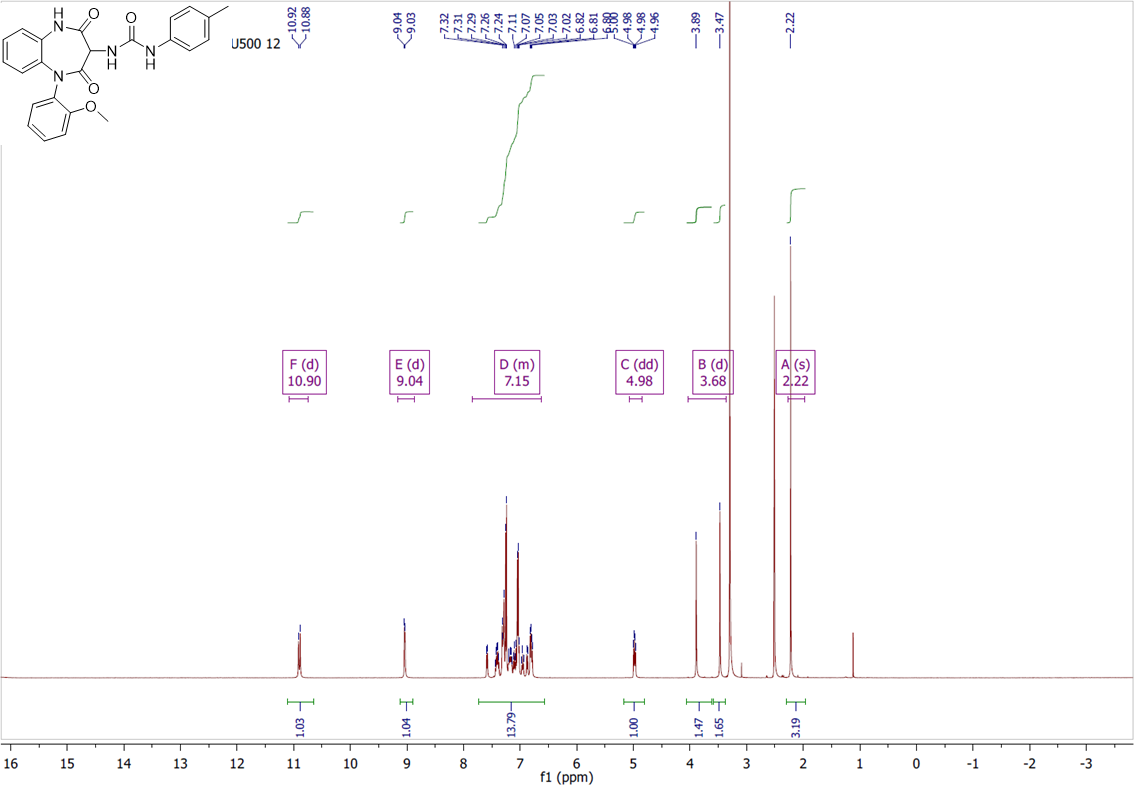
**


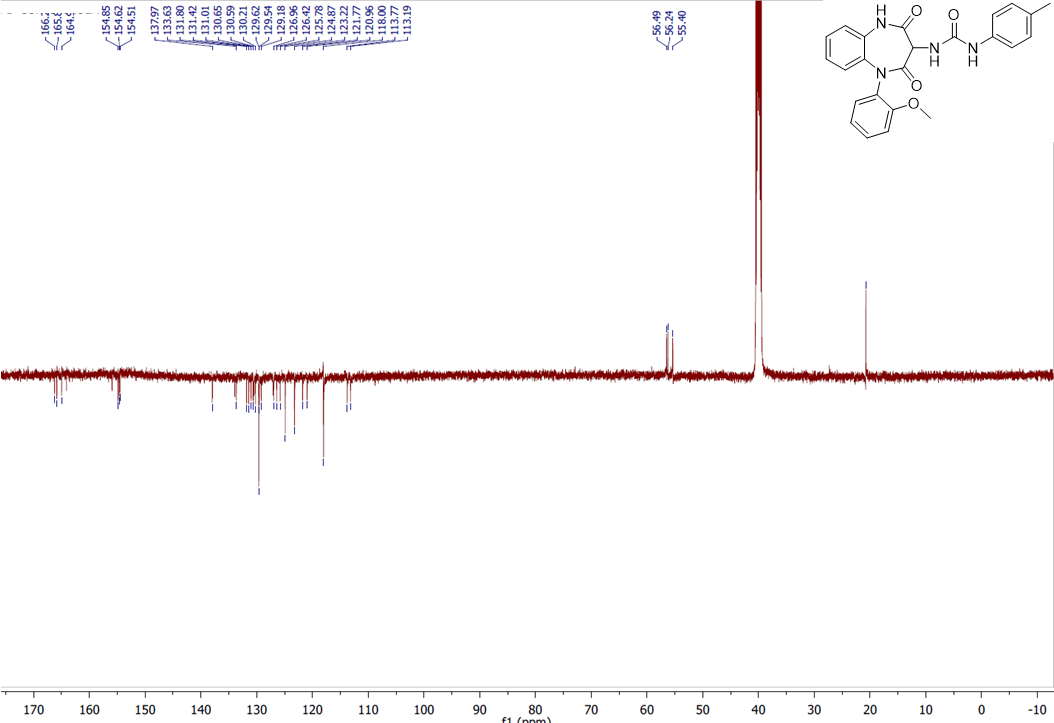


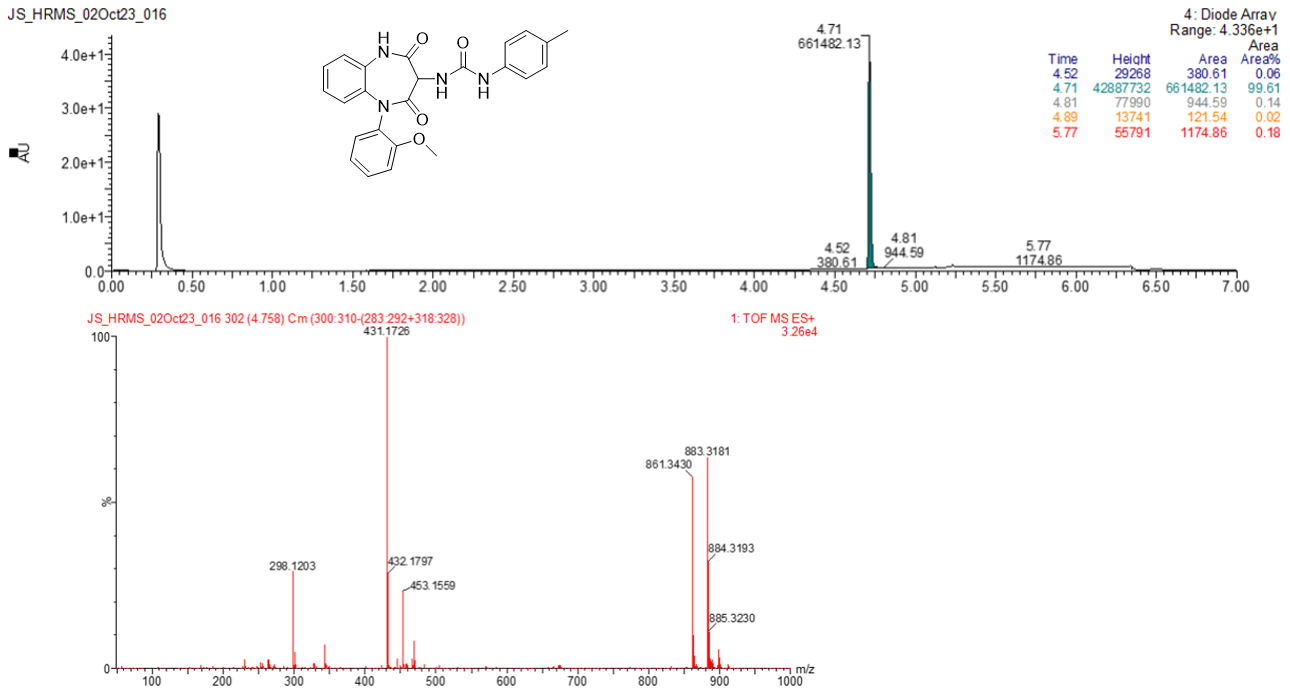


**^1^H NMR spectrum (500 MHz, DMSO-*d_6_*), ^13^C NMR spectrum (126 MHz, DMSO-*d_6_*) and LCMS trace of compound 7**


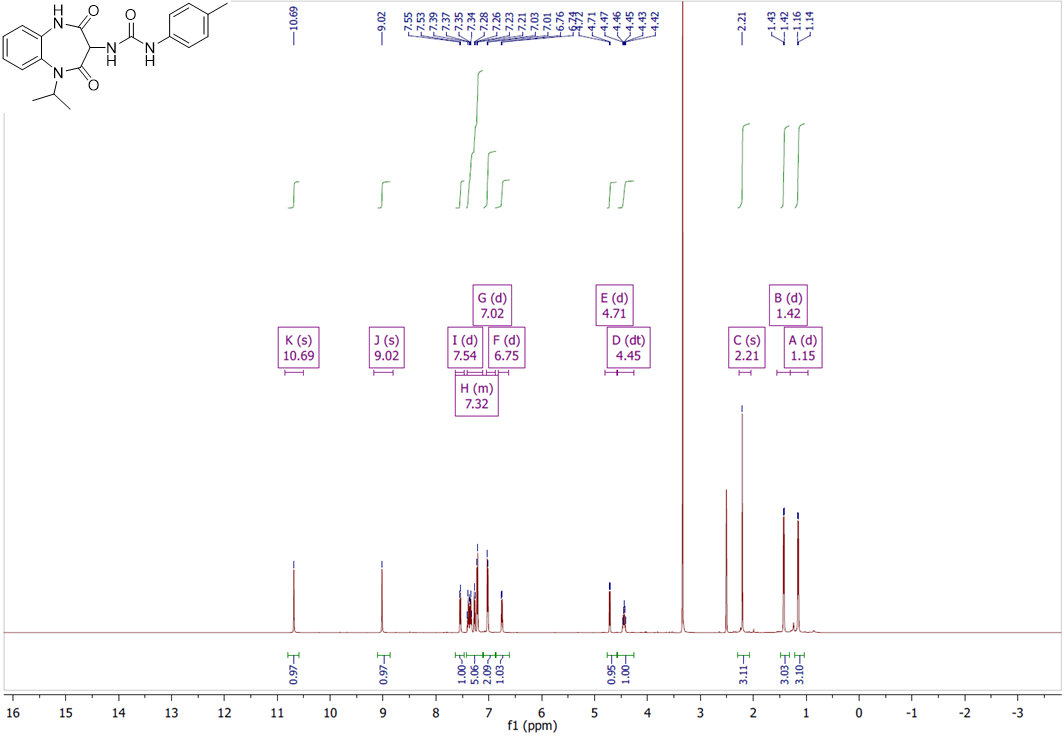


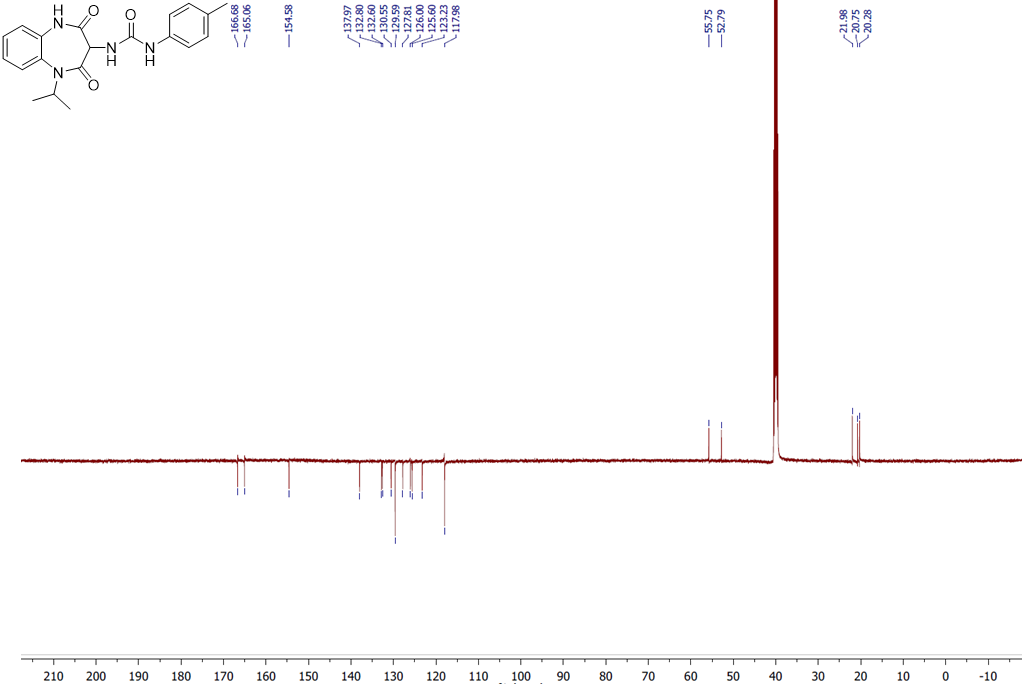


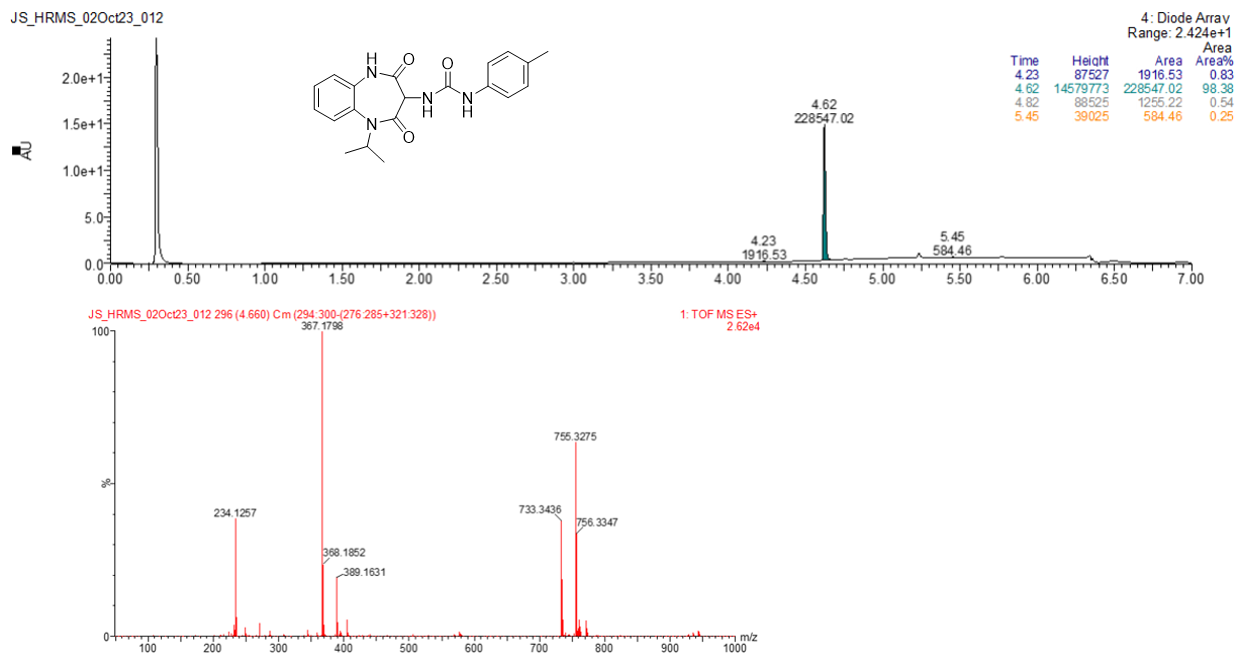


**^1^H NMR spectrum (500 MHz, DMSO-*d_6_*), ^13^C NMR spectrum (126 MHz, DMSO-*d_6_*) and LCMS trace of compound 8**

**
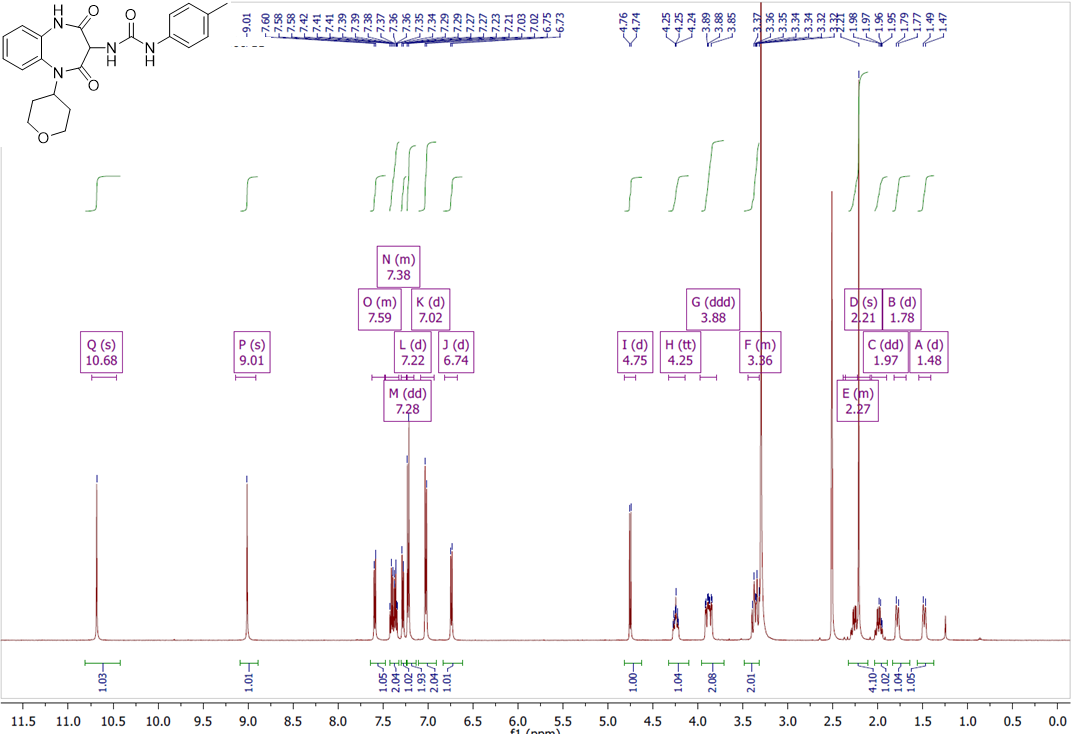
**

**
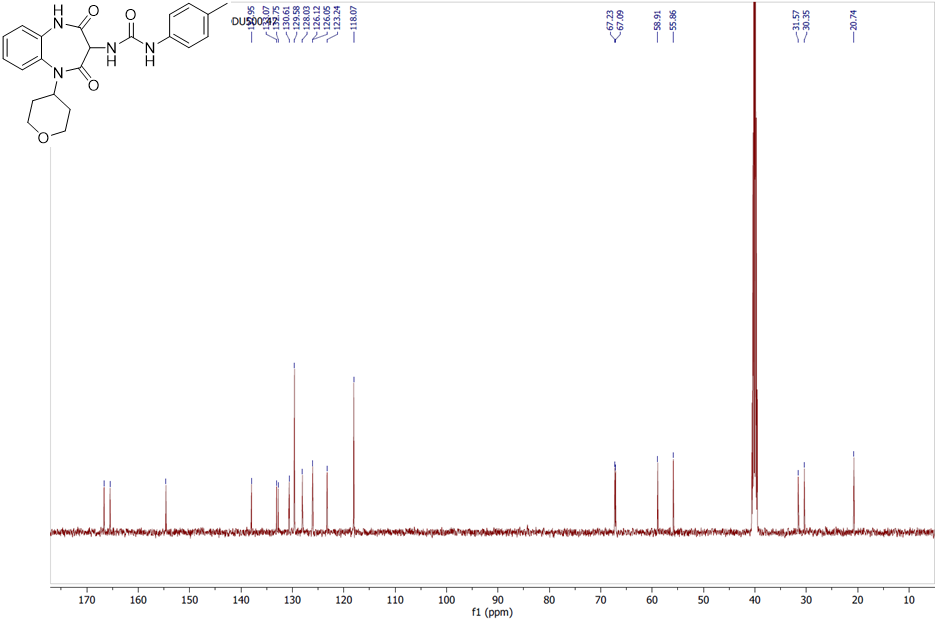
**

**
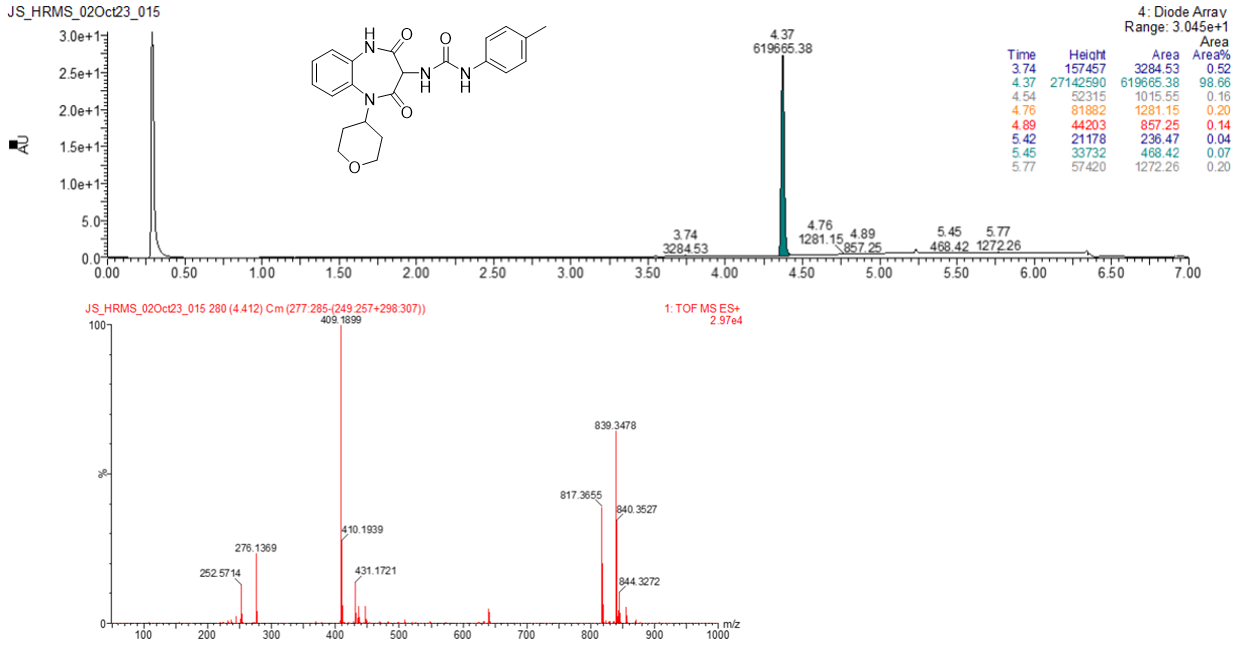
**

**^1^H NMR spectrum (500 MHz, DMSO-*d_6_*) and ^13^C NMR spectrum (126 MHz, DMSO-*d_6_*) and HPLC trace of compound 9**

**
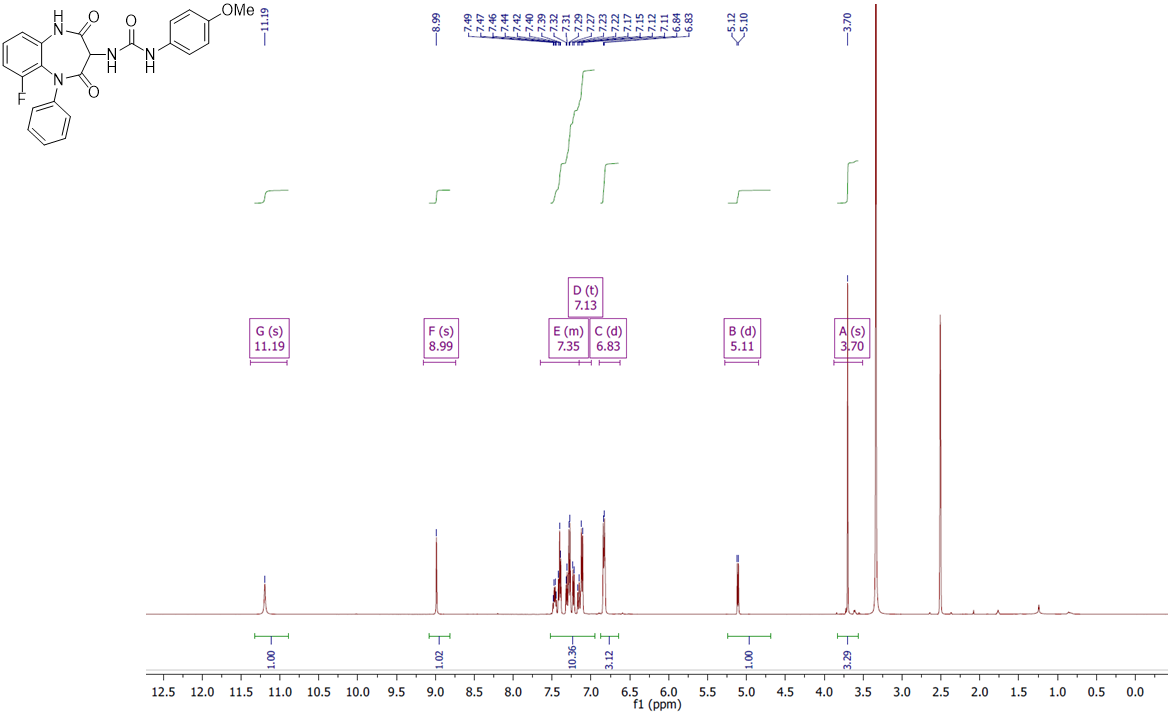
**

**
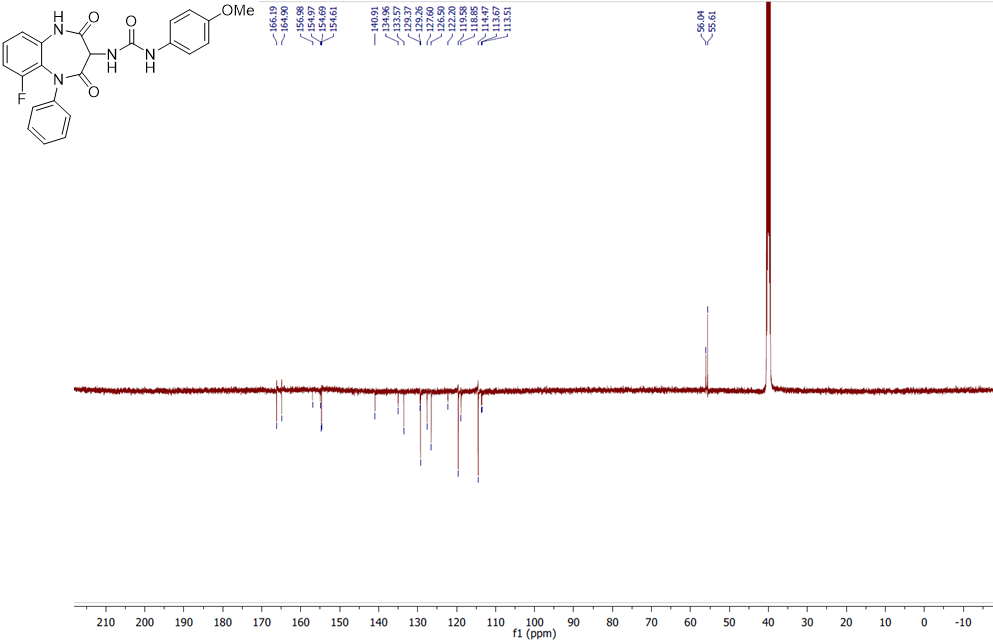
**

**
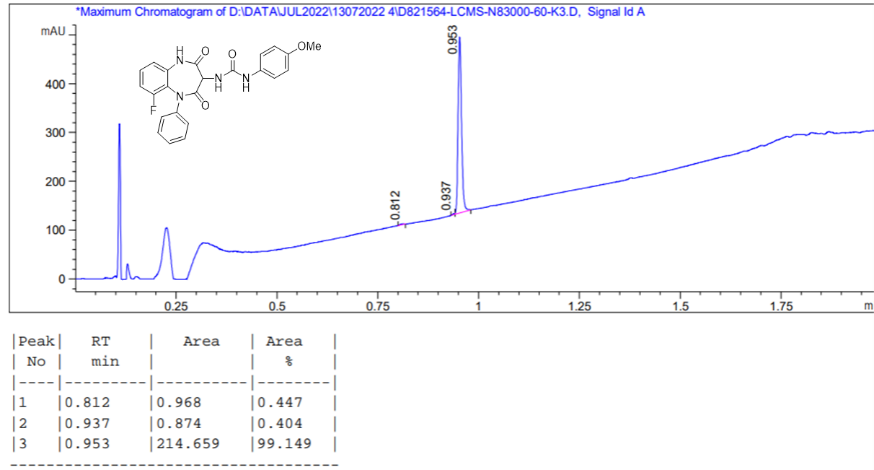
**

**^1^H NMR spectrum (500 MHz, DMSO-*d_6_*), ^13^C NMR spectrum (126 MHz, DMSO-*d_6_*) and LCMS trace of compound 10**

**
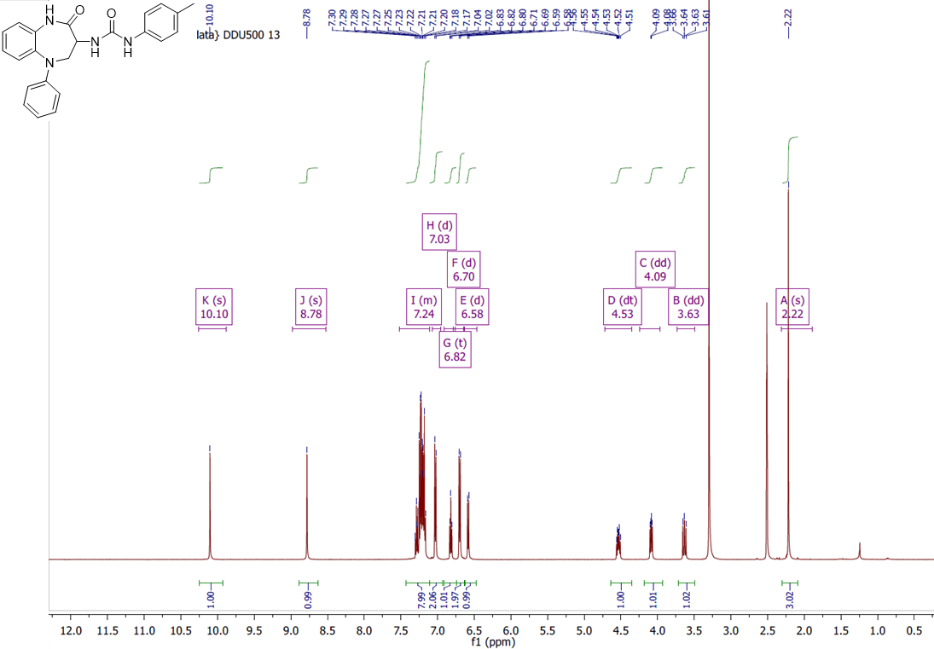
**

**^
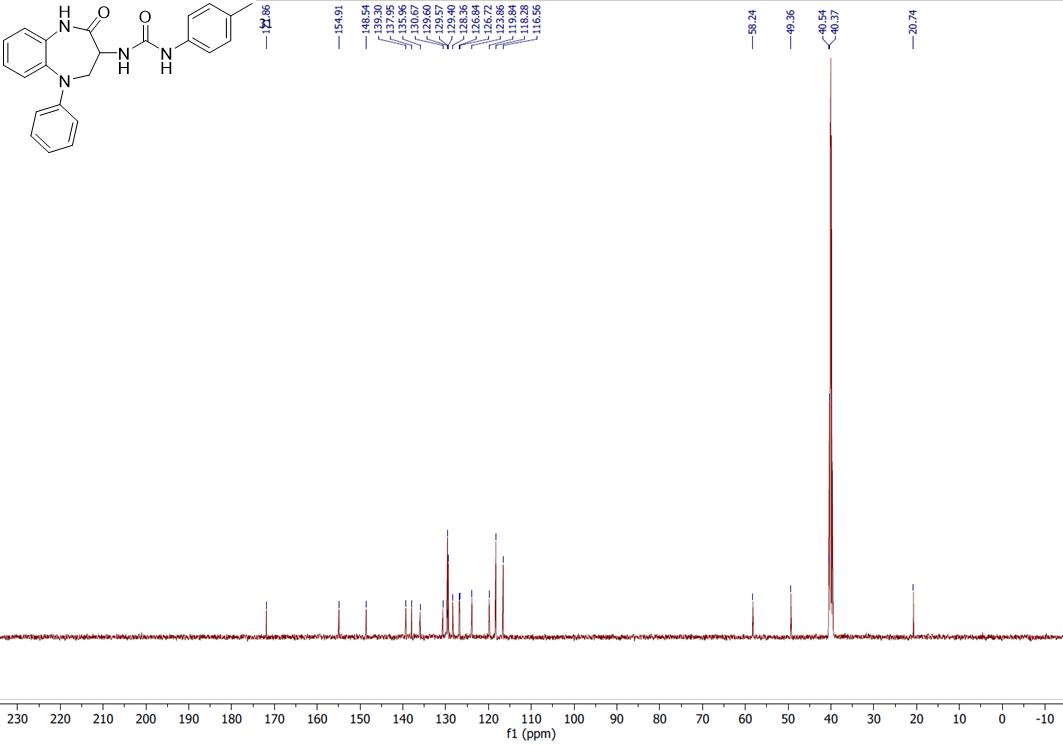
^**

**^
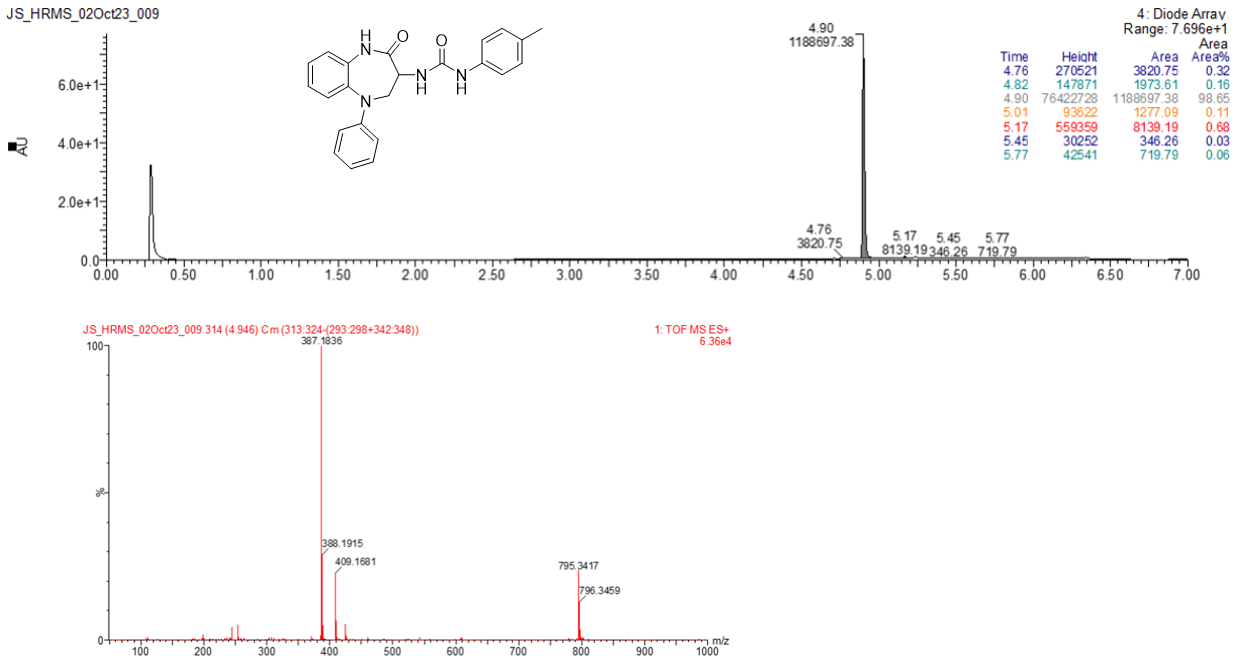
^**

**^1^H NMR spectrum (500 MHz, DMSO-*d_6_*), ^13^C NMR spectrum (126 MHz, DMSO-*d_6_*) and LCMS trace of compound 11**

**
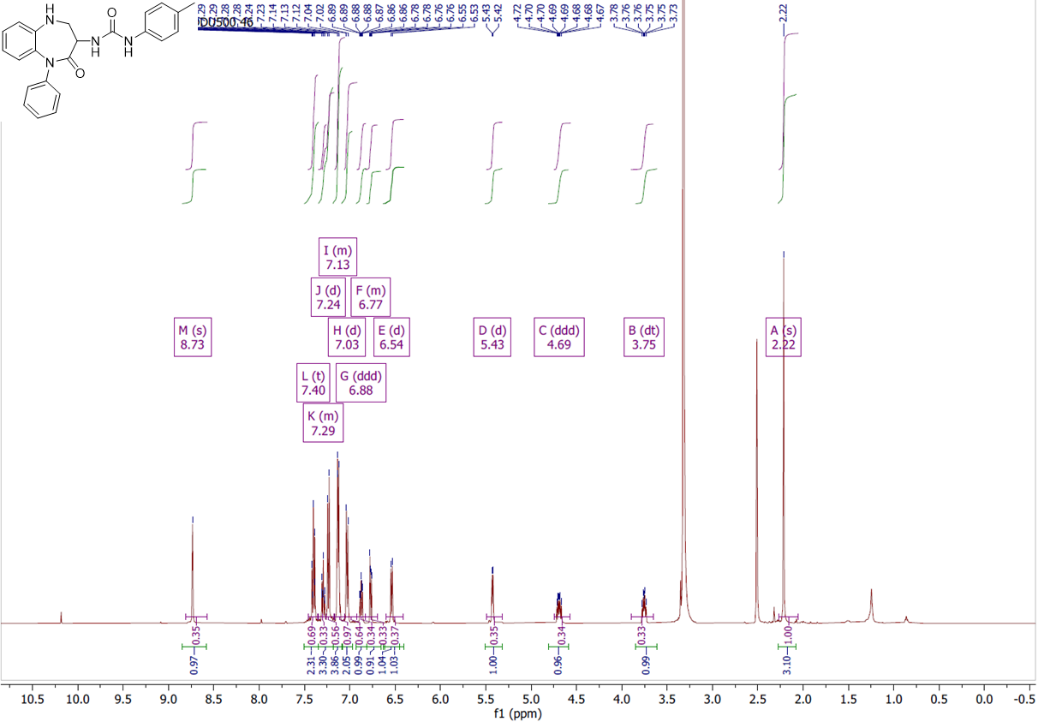
**

**
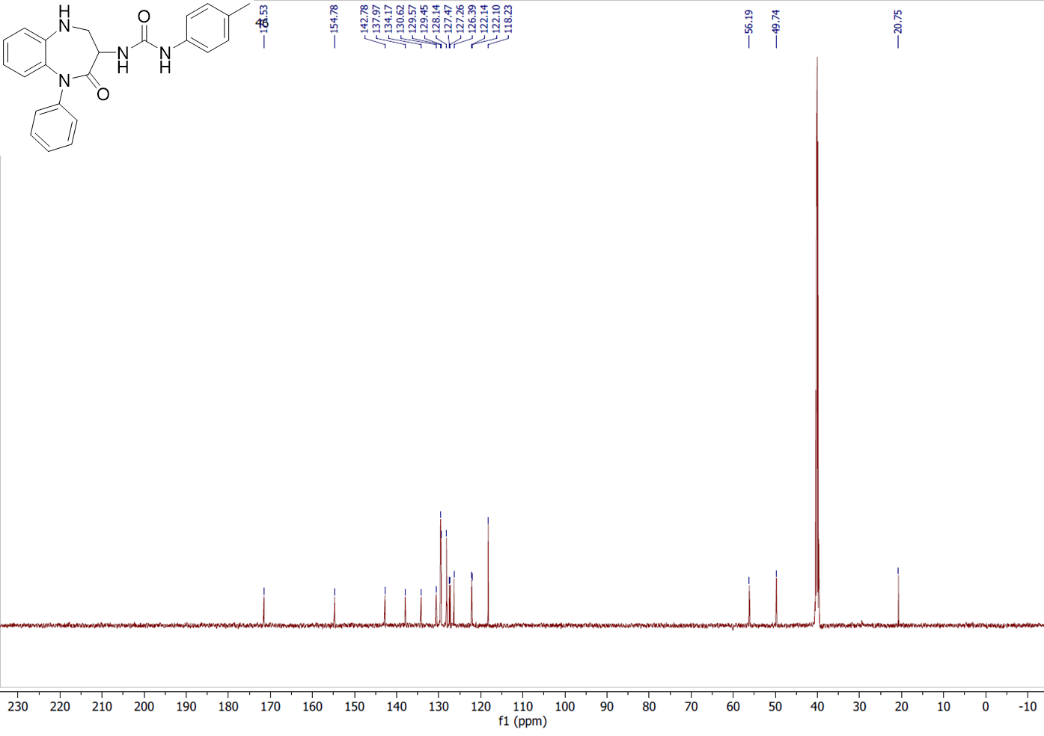
**

**
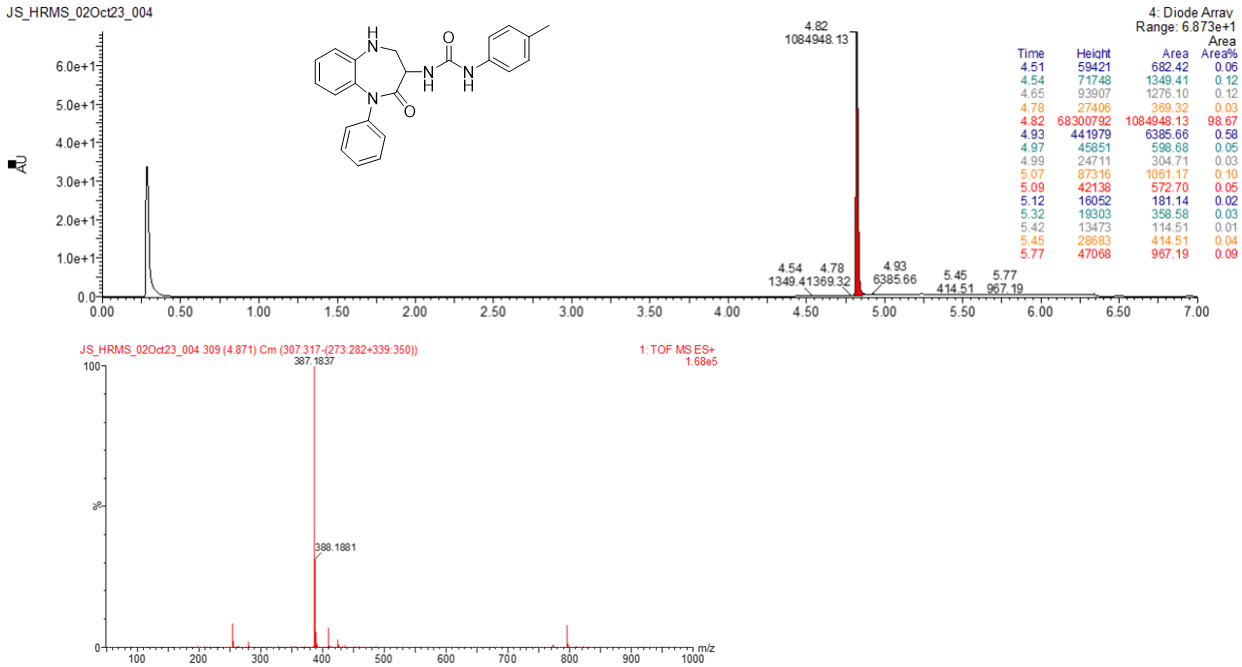
**

**^1^H NMR spectrum (500 MHz, DMSO-*d_6_*), ^13^C NMR spectrum (126 MHz, DMSO-*d_6_*) and LCMS trace of compound 13**

**
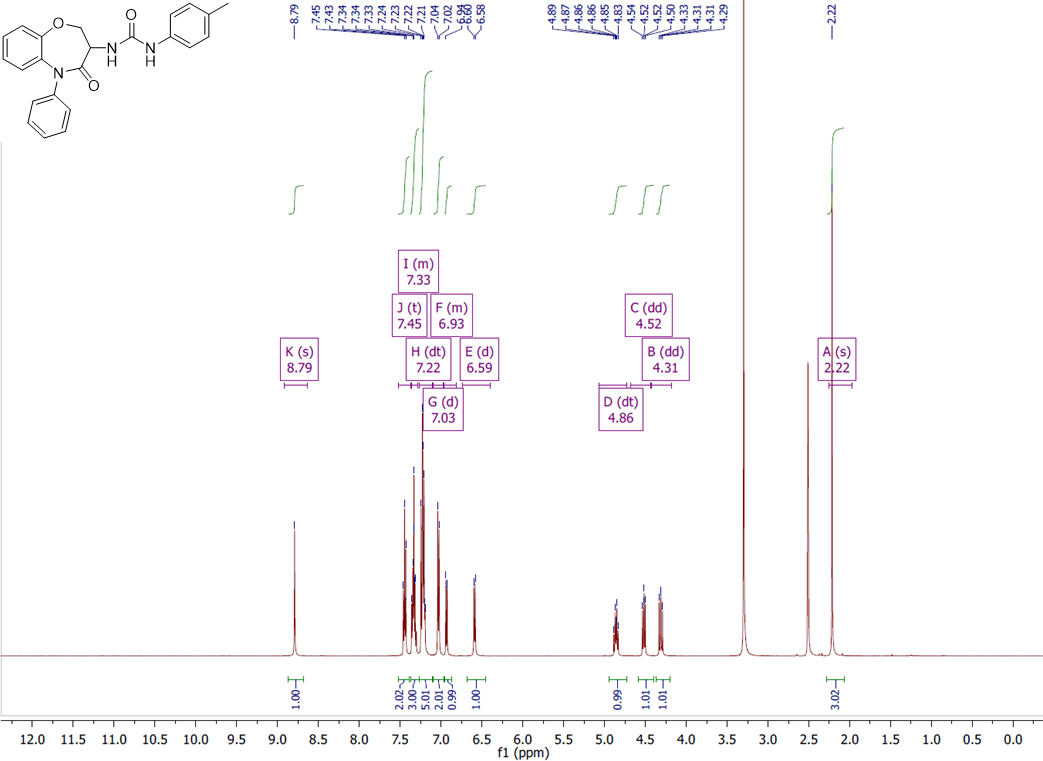
**

**
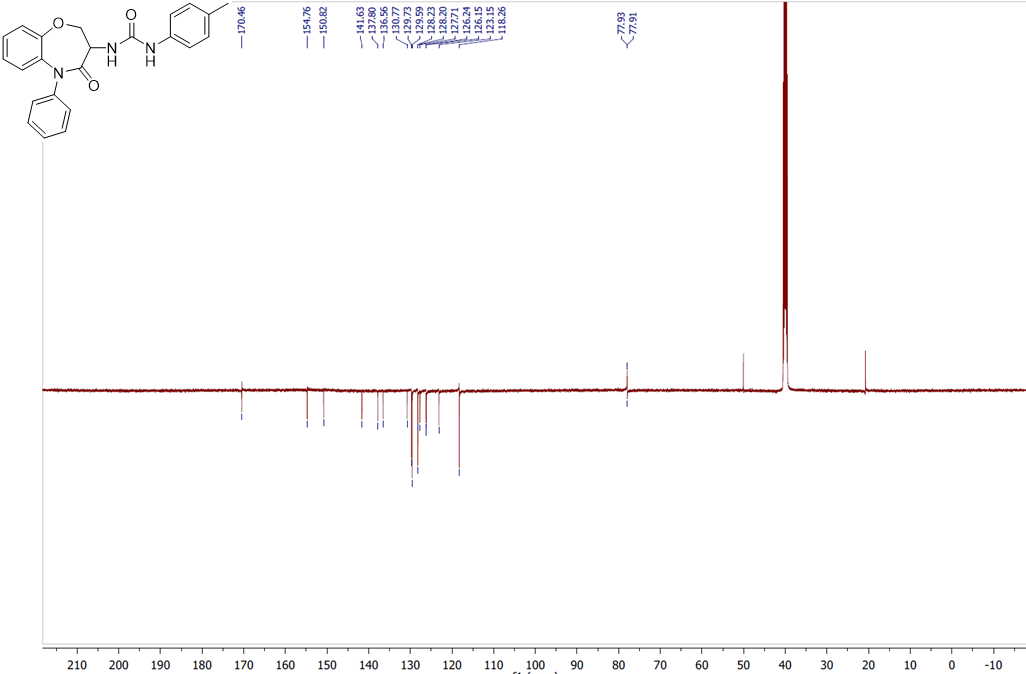
**

**
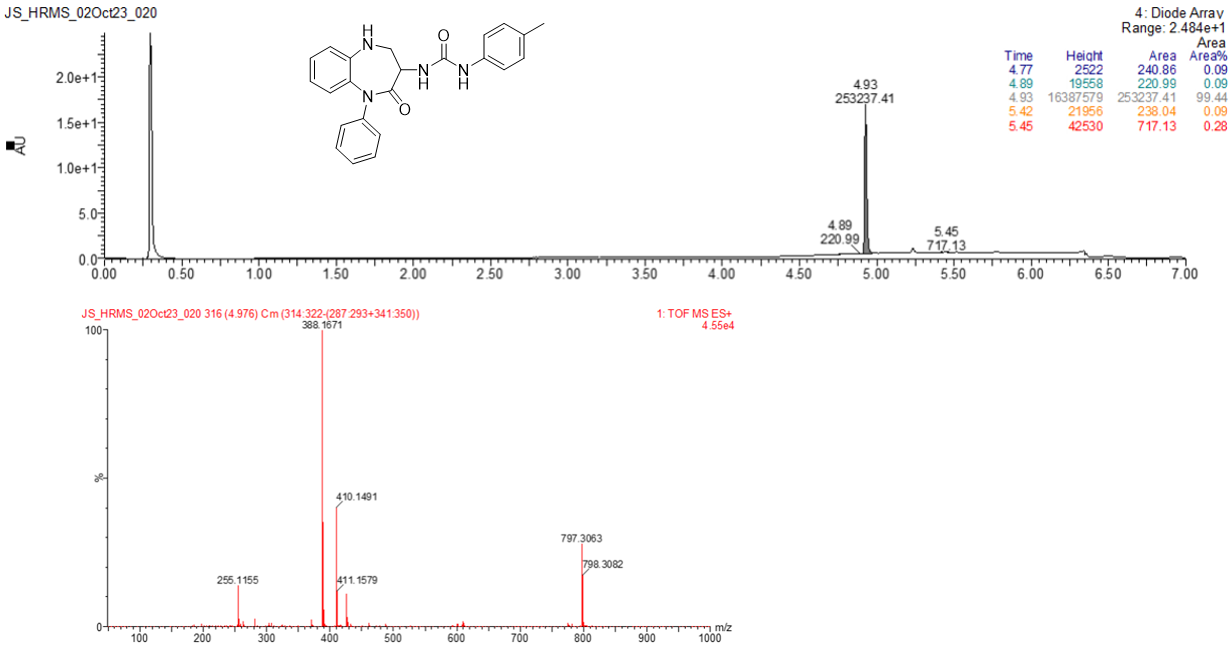
**

**^1^H NMR spectrum (500 MHz, DMSO-*d_6_*) of compound 14 (enantiomer 1)**

**
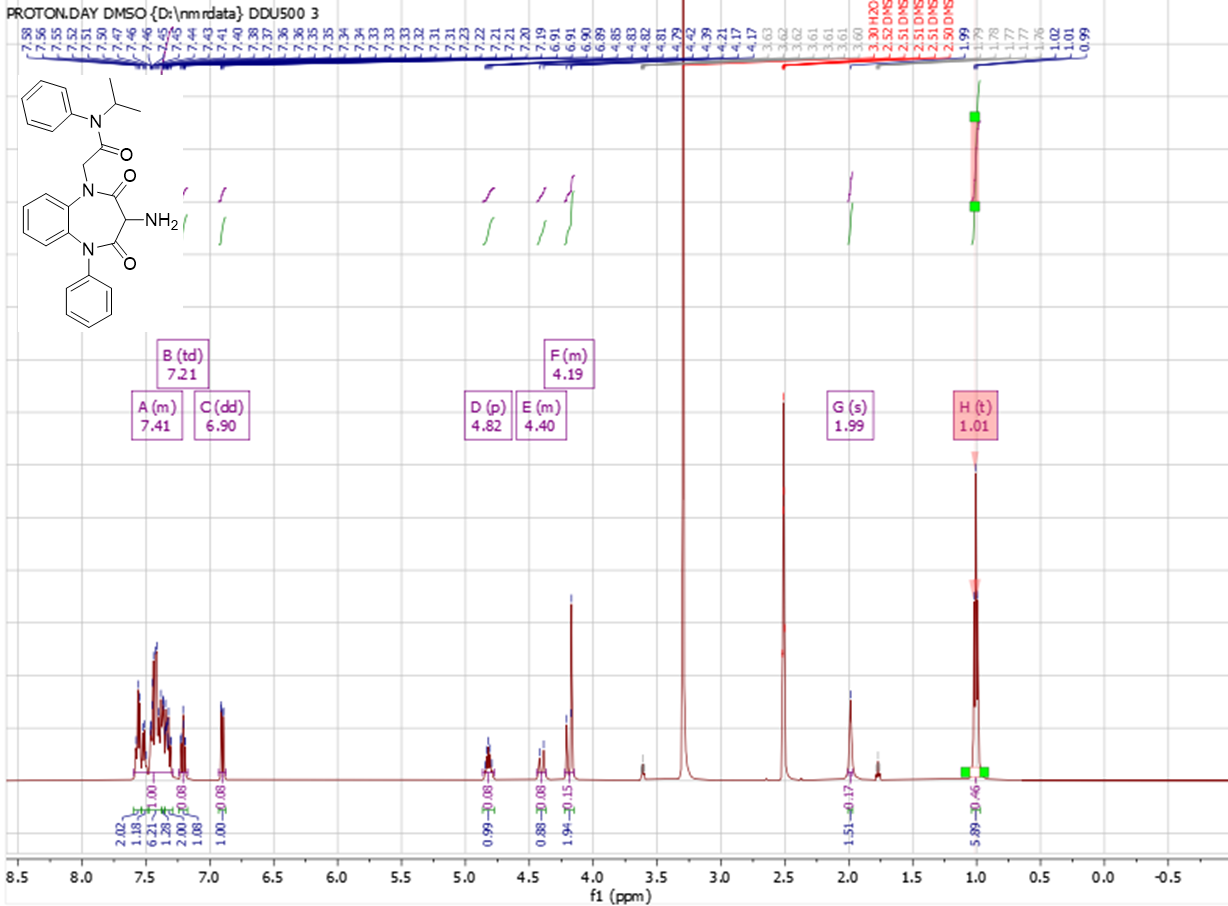
**

**^1^H NMR spectrum (500 MHz, DMSO-*d_6_*), ^13^C NMR spectrum (126 MHz, DMSO-*d_6_*) and LCMS trace of compound 15**

**
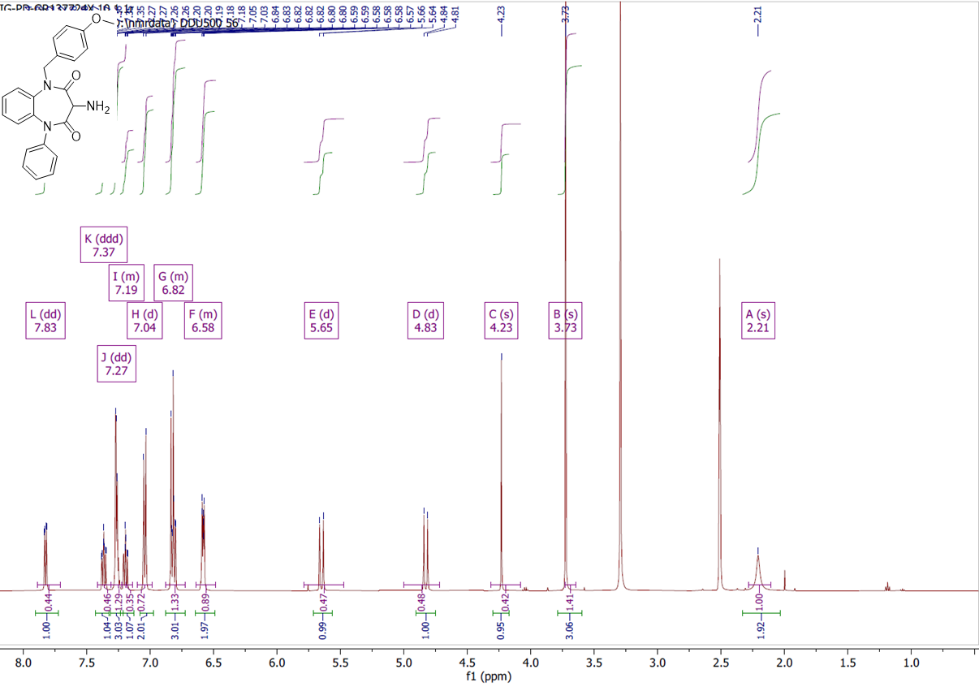
**

**
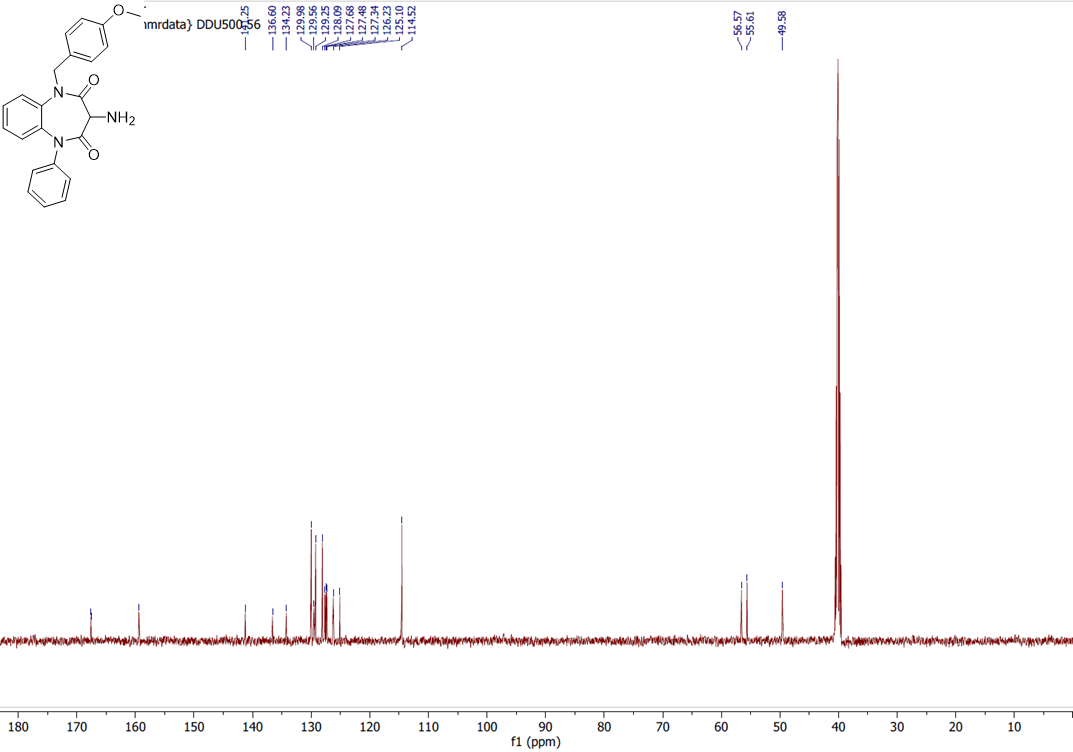
**

**
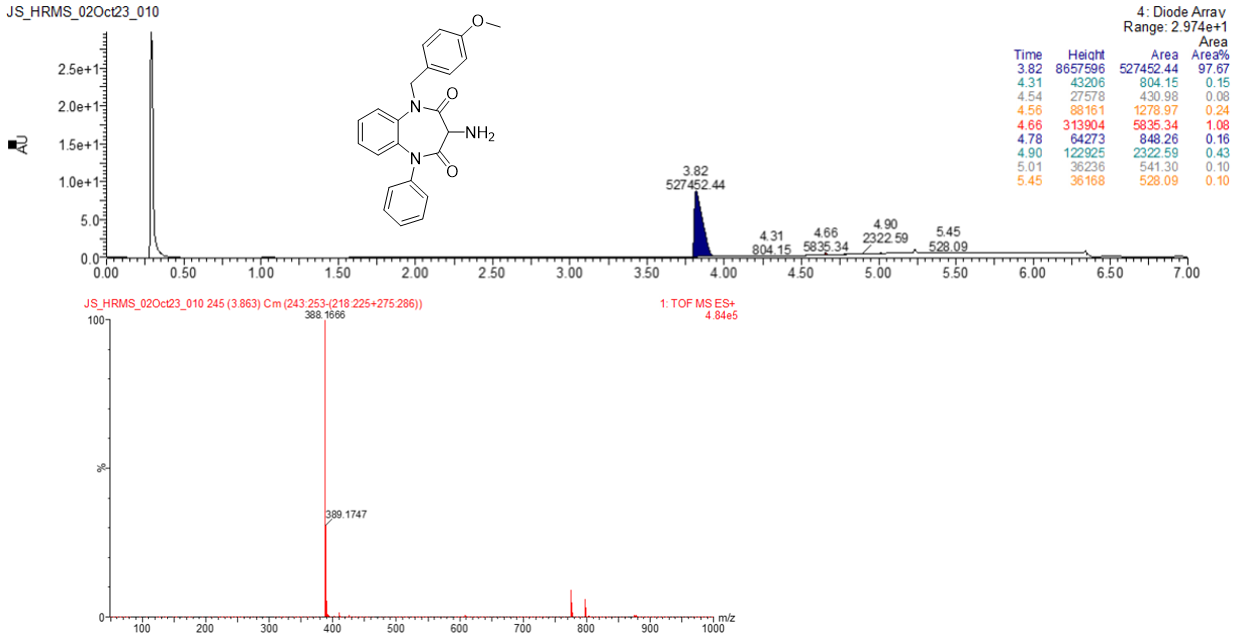
**

**^1^H NMR spectrum (500 MHz, DMSO-*d_6_*) of compound 16**

**^
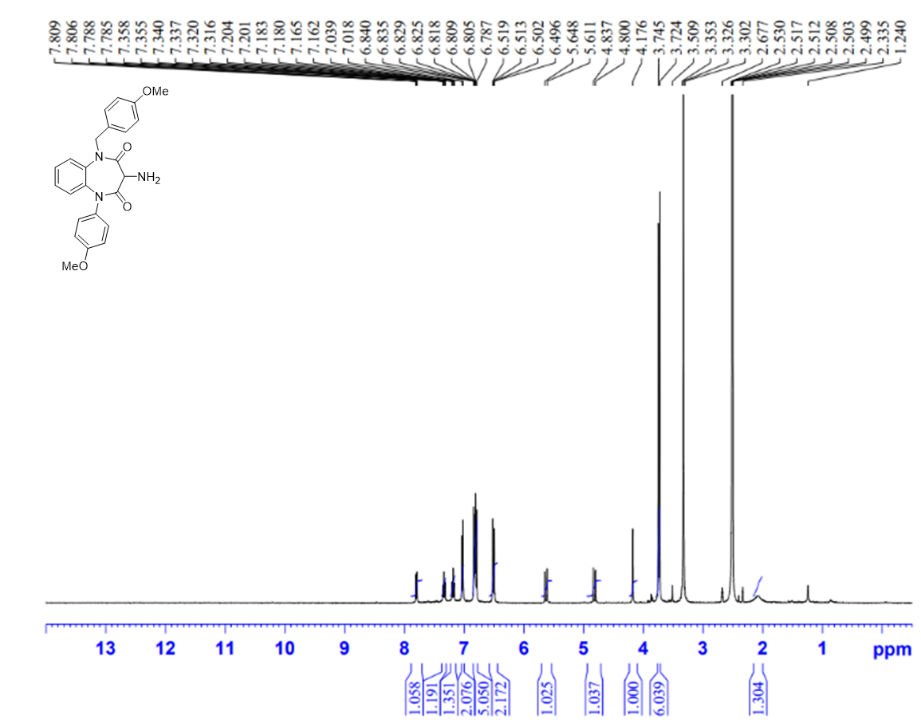
^**

**^1^H NMR spectrum (500 MHz, DMSO-*d_6_*), ^13^C NMR spectrum (126 MHz, DMSO-*d_6_*) and HPLC trace of compound 17**

**
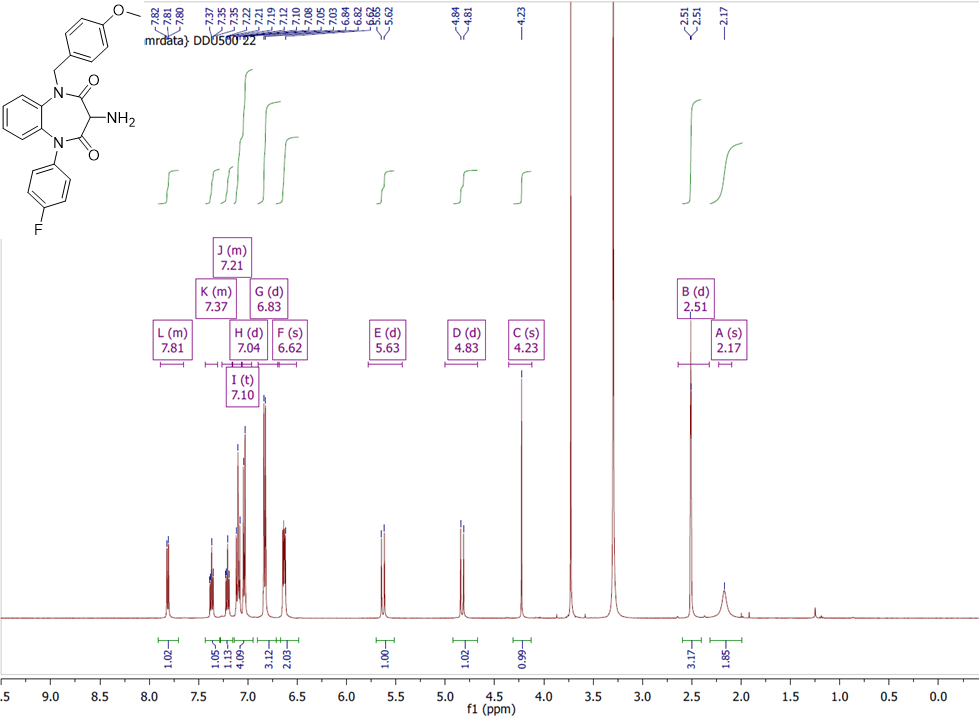
**

**
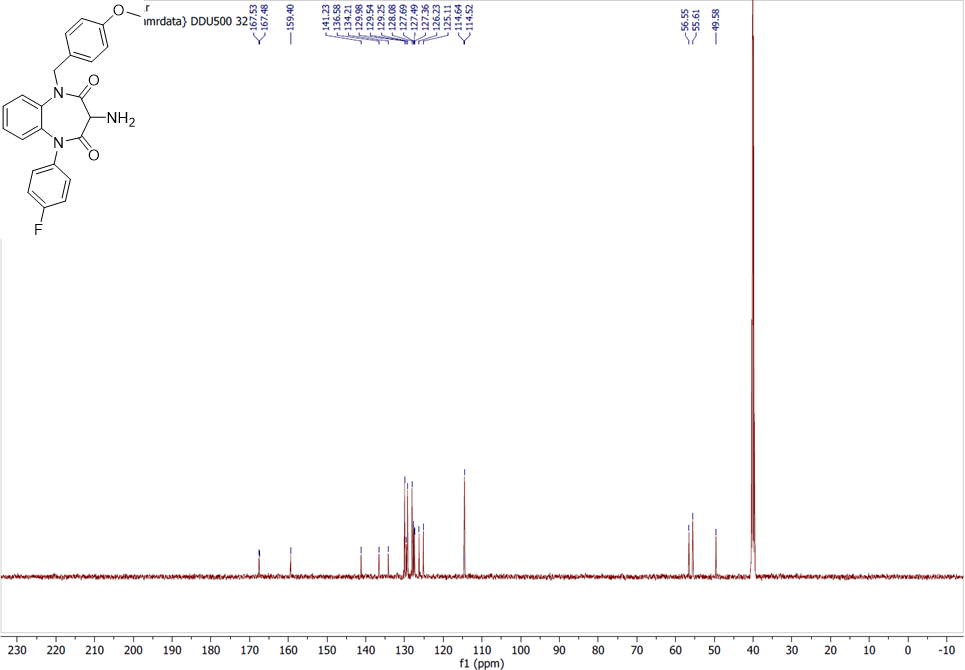
**

**
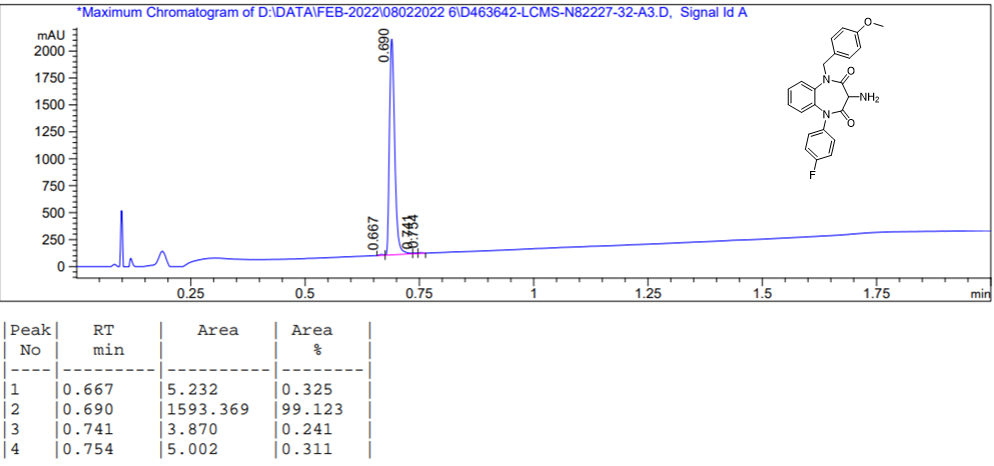
**

**^1^H NMR spectrum (500 MHz, DMSO-*d_6_*), ^13^C NMR spectrum (126 MHz, DMSO-*d_6_*) and HPLC trace of compound 18**

**
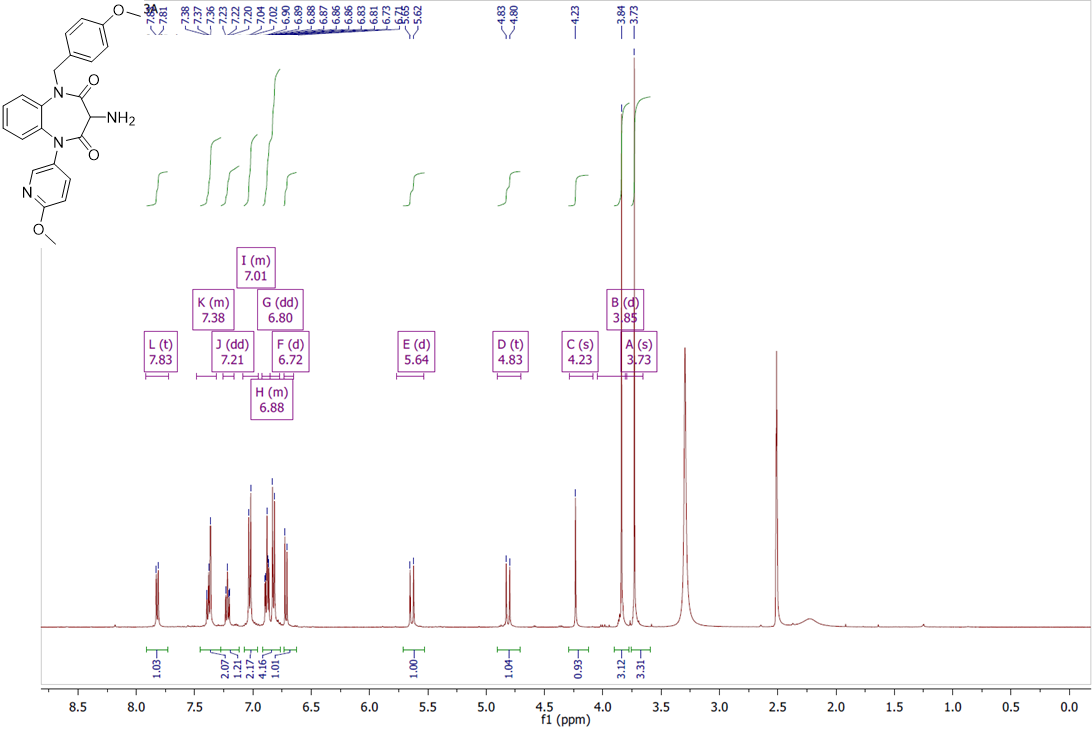
**

**
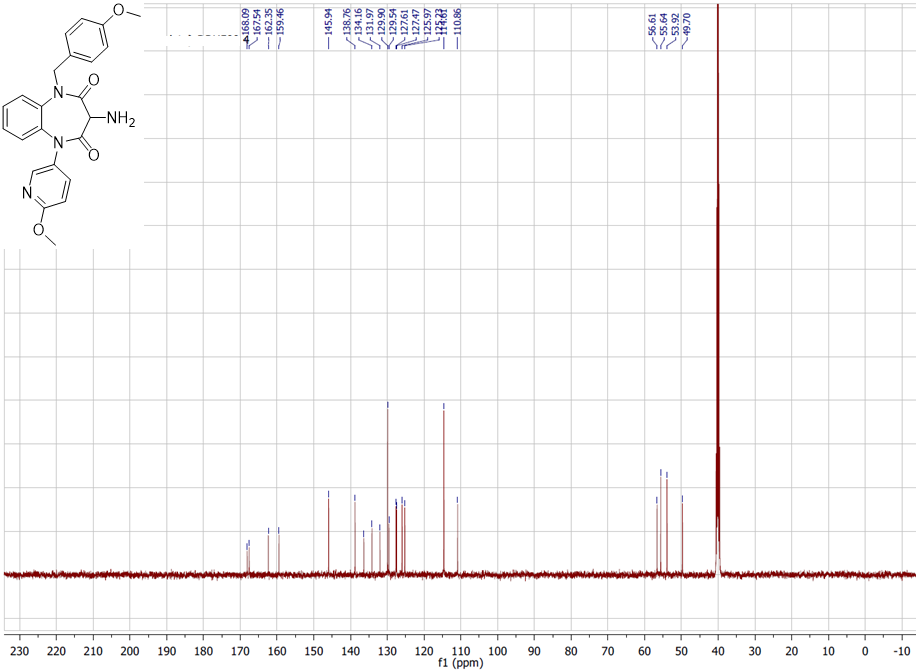
**

**
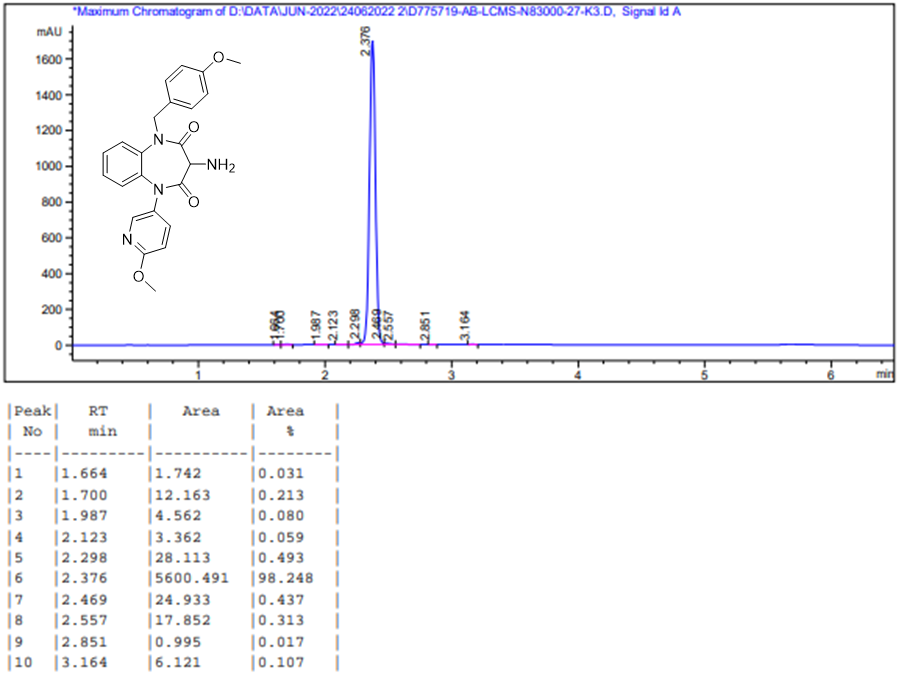
**

**^1^H NMR spectrum (500 MHz, DMSO-*d_6_*), ^13^C NMR spectrum (126 MHz, DMSO-*d_6_*) and LCMS trace of compound 19**

**
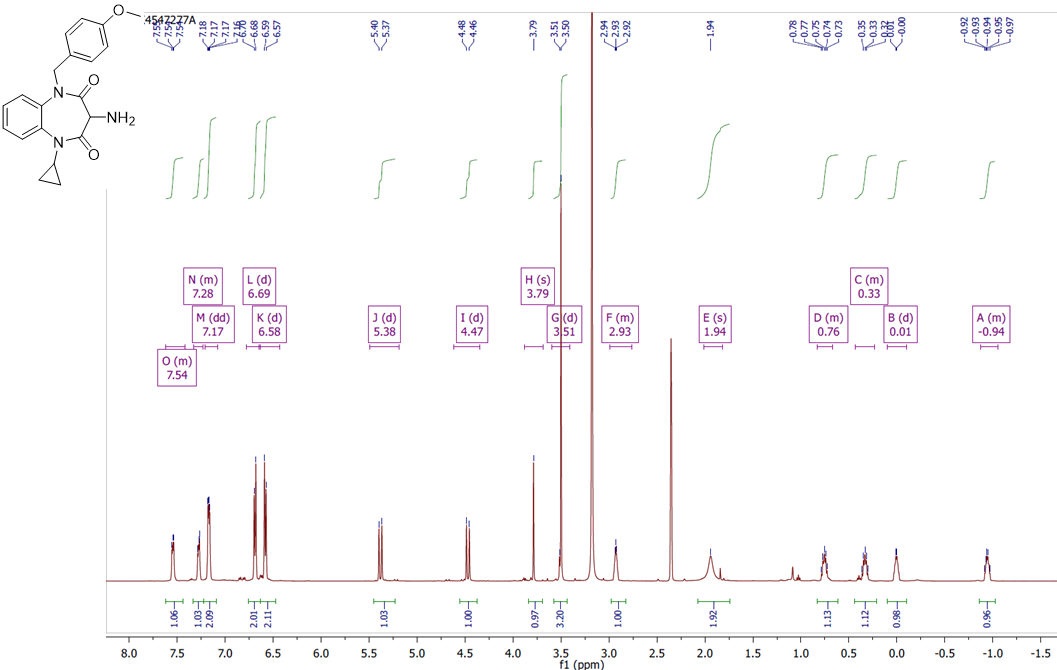
**

**
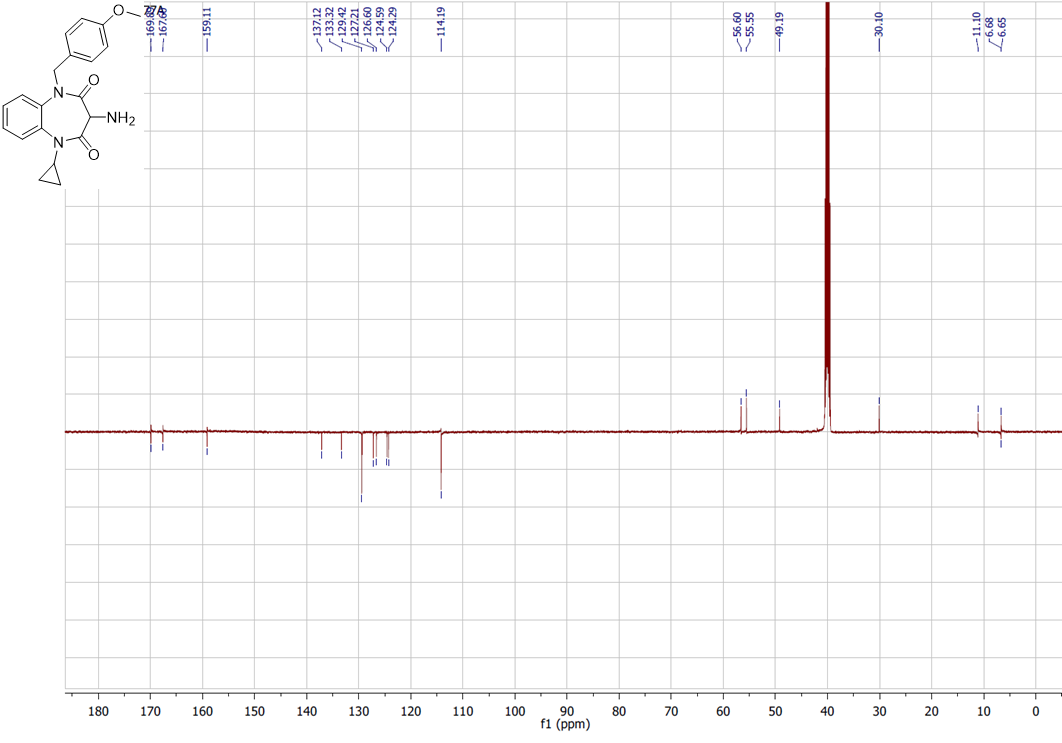
**

**
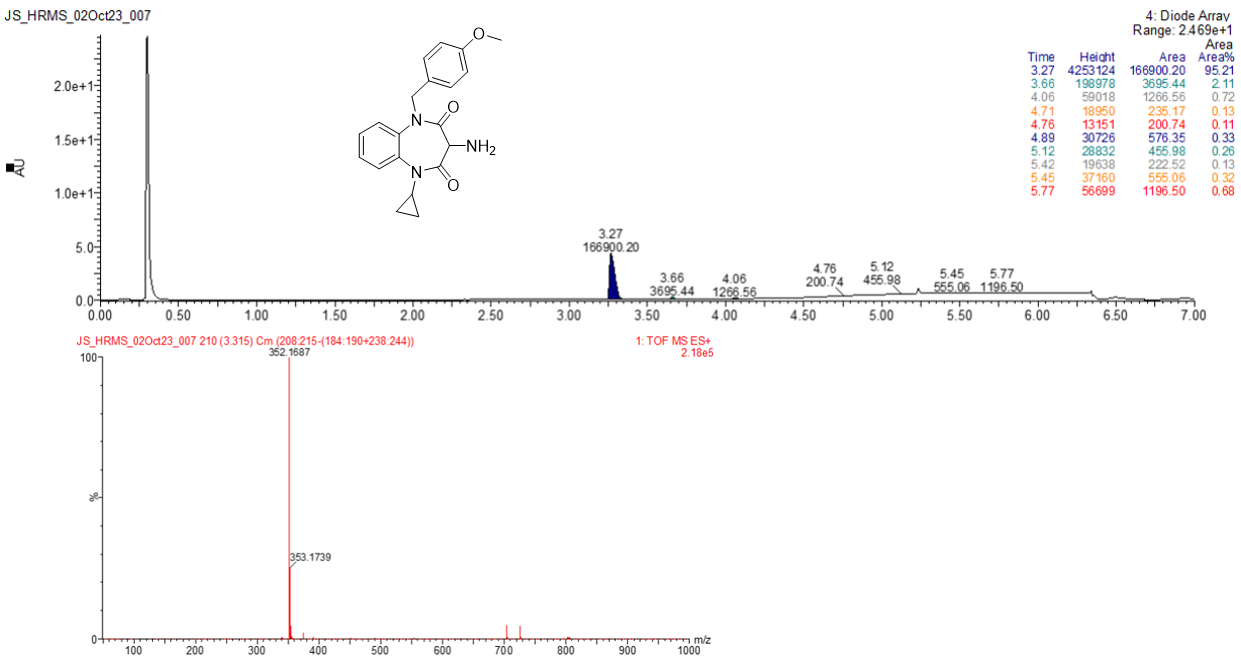
**

**^1^H NMR spectrum (500 MHz, DMSO-*d_6_*), ^13^C NMR spectrum (126 MHz, DMSO-*d_6_*) and LCMS trace of compound 20**

**
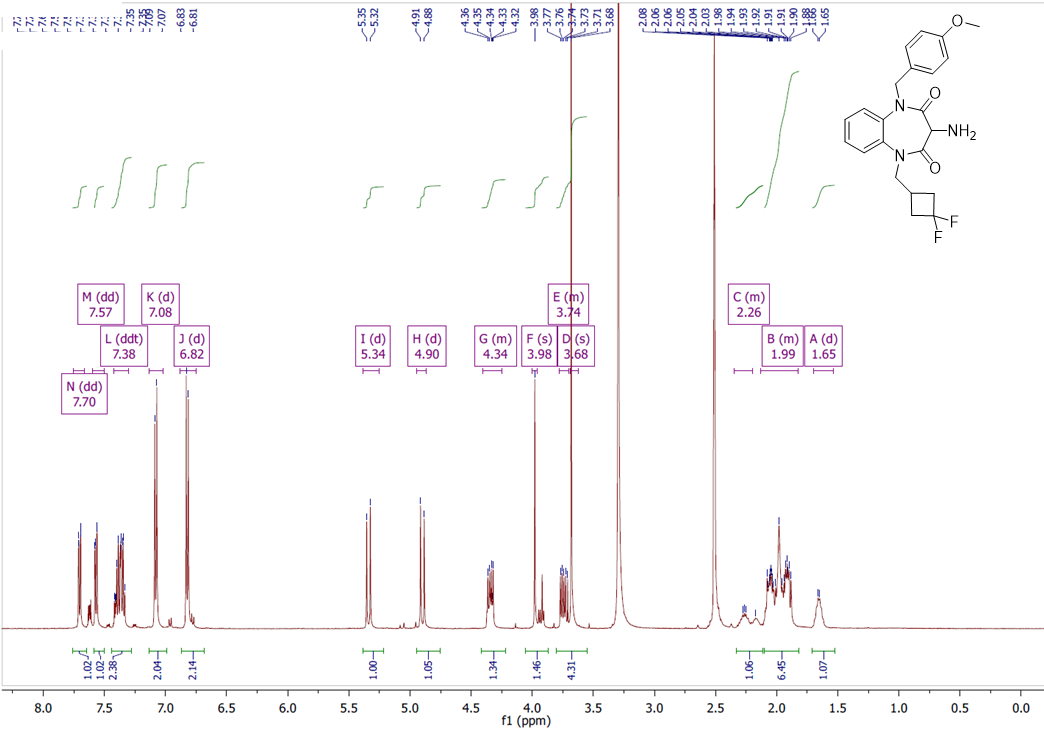
**

**
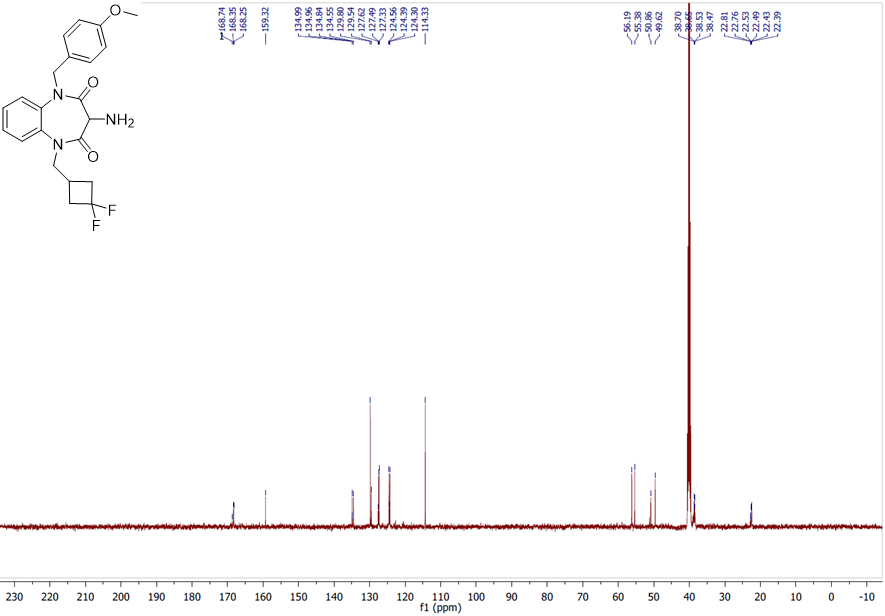
**

**
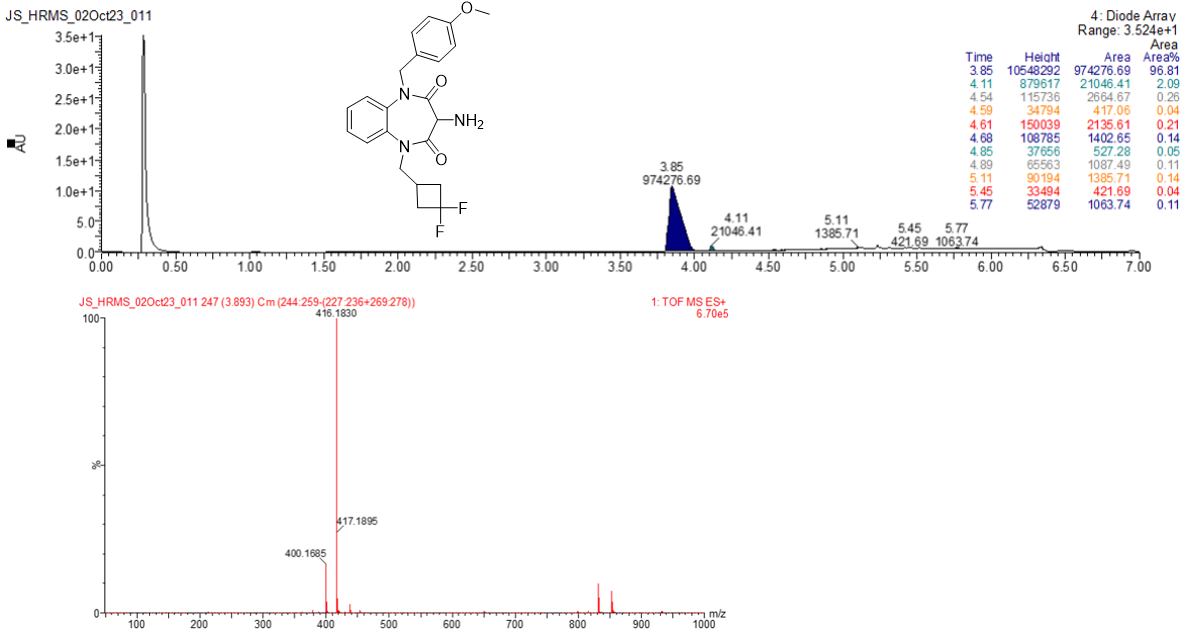
**

**^1^H NMR spectrum (500 MHz, DMSO-*d_6_*) and ^13^C NMR spectrum (126 MHz, DMSO-*d_6_*) of compound 21**

**
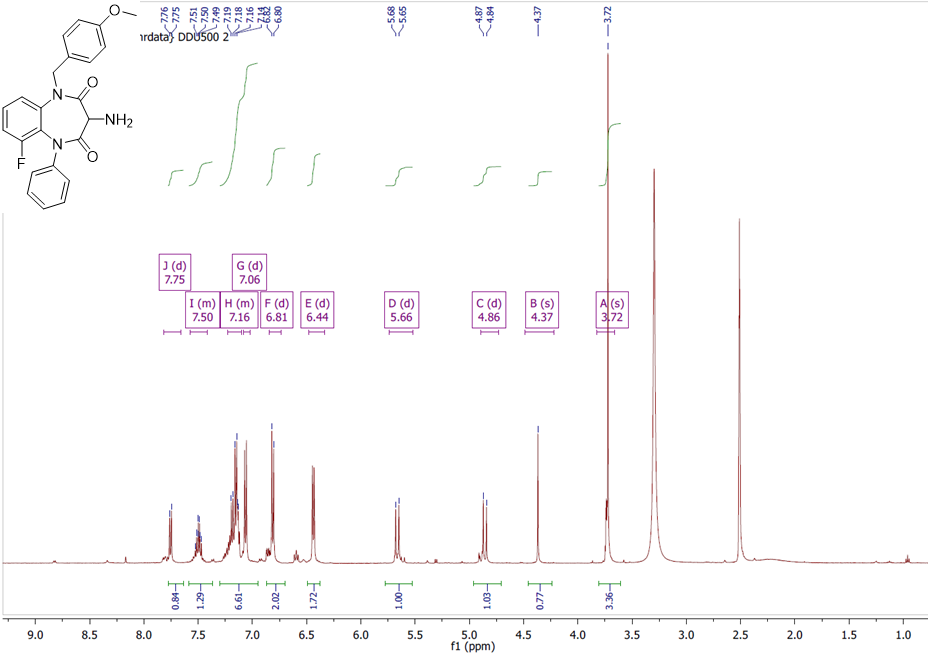
**

**
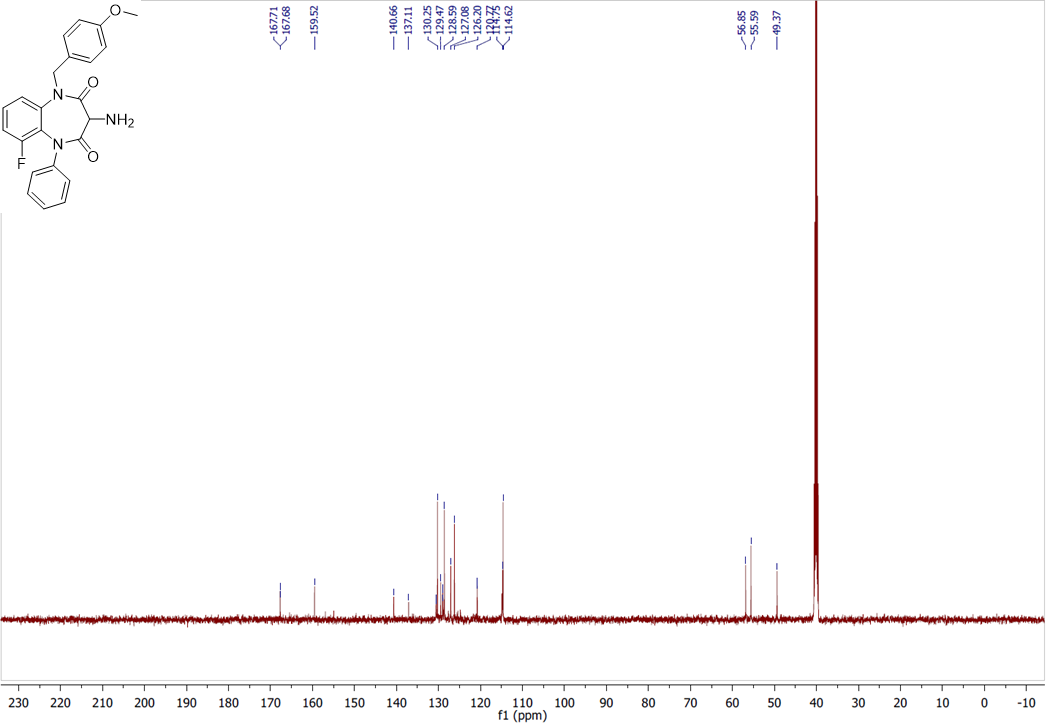
**

**^1^H NMR spectrum (500 MHz, DMSO-*d_6_*) and ^13^C NMR spectrum (126 MHz, DMSO-*d_6_*) and HPLC trace of compound 22**

**
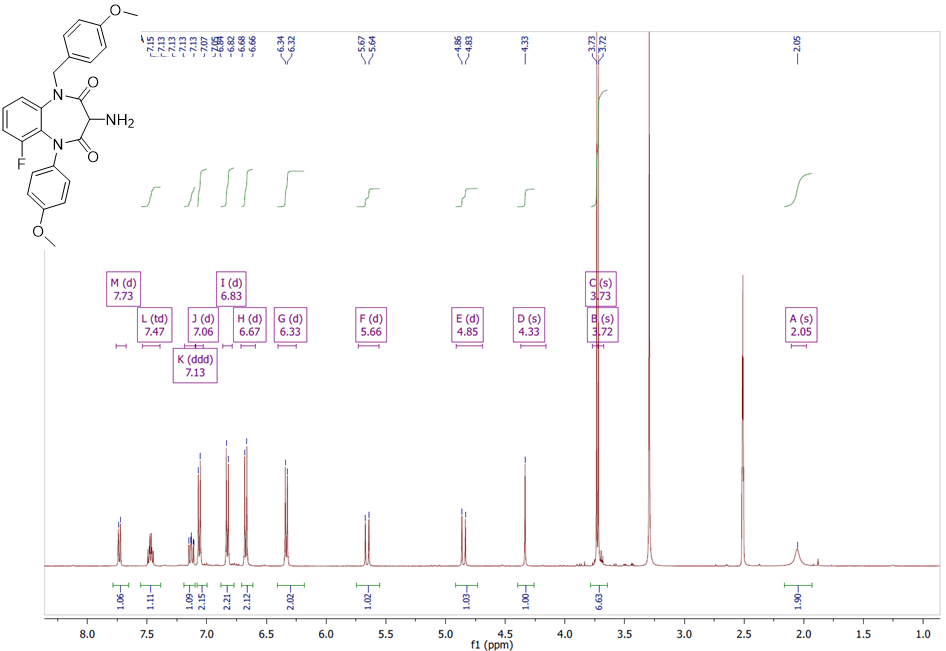
**

**
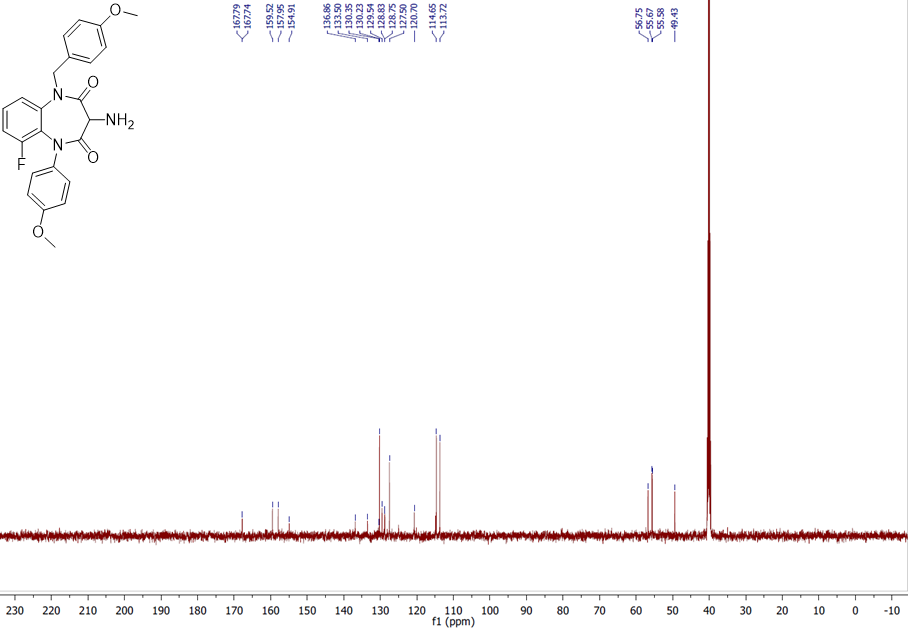
**

**
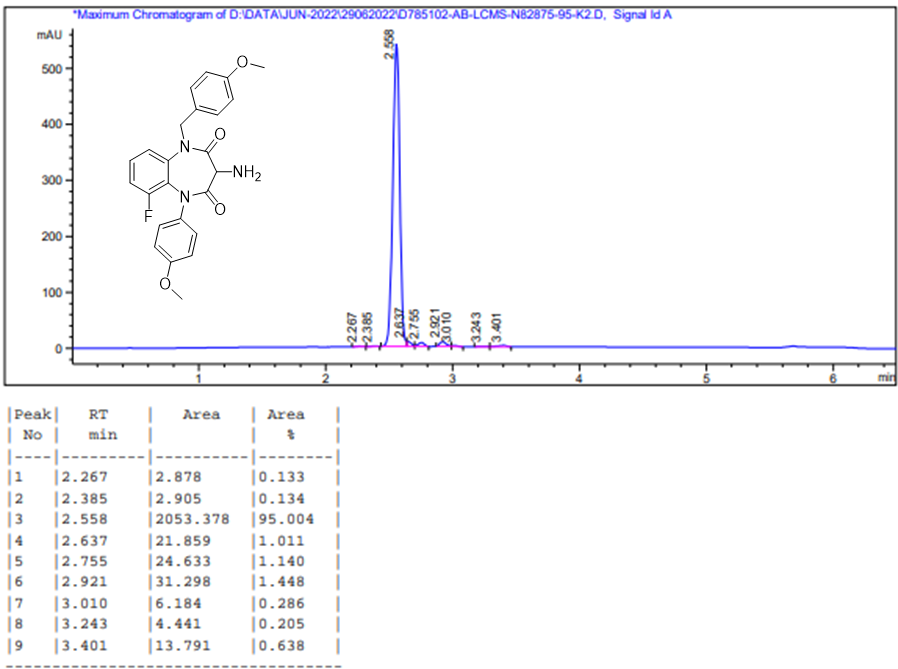
**

**^1^H NMR spectrum (500 MHz, DMSO-*d_6_*), ^13^C NMR spectrum (126 MHz, DMSO-*d_6_*) and LCMS trace of compound 23**

**
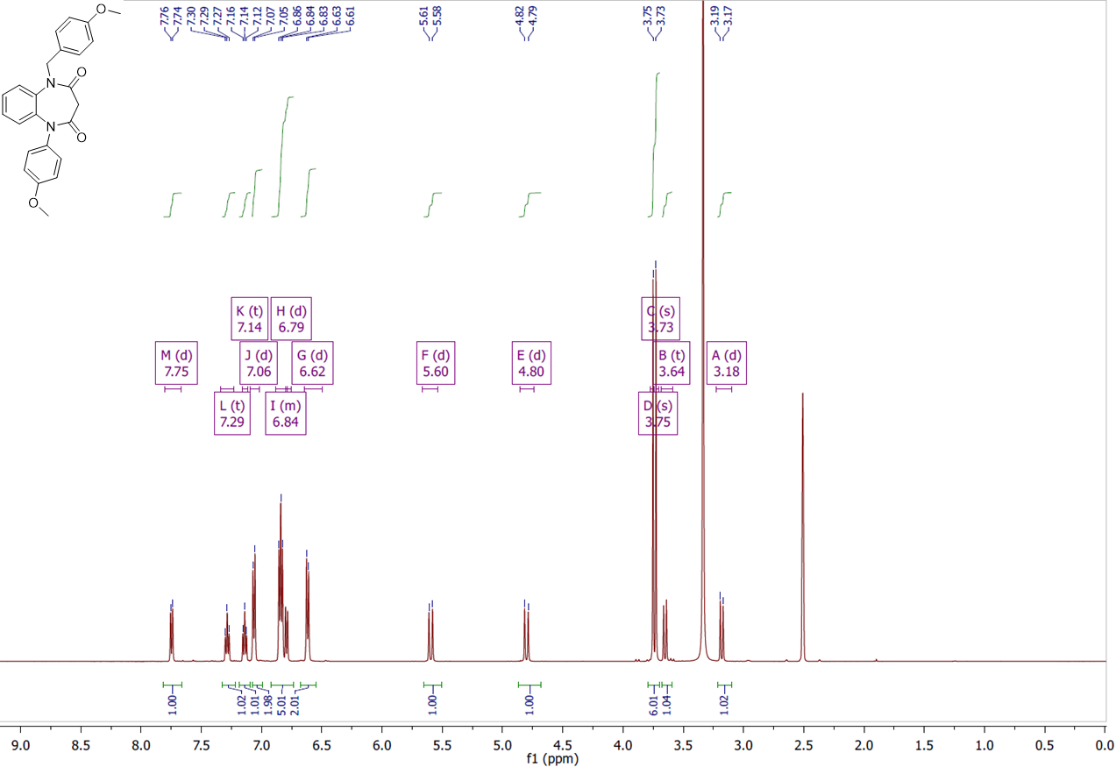
**

**
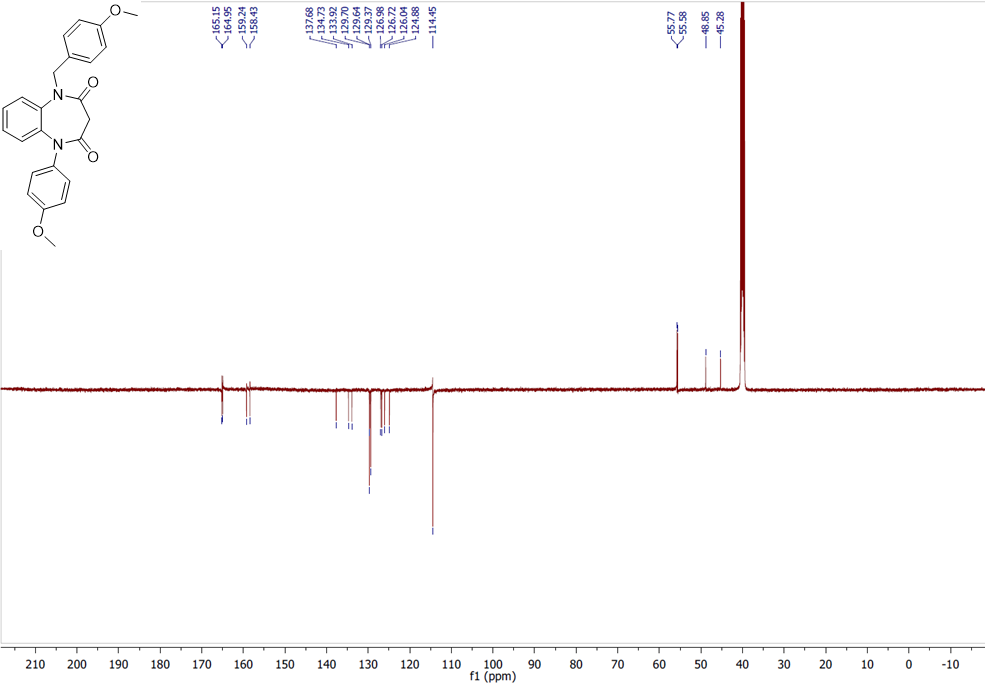
**

***
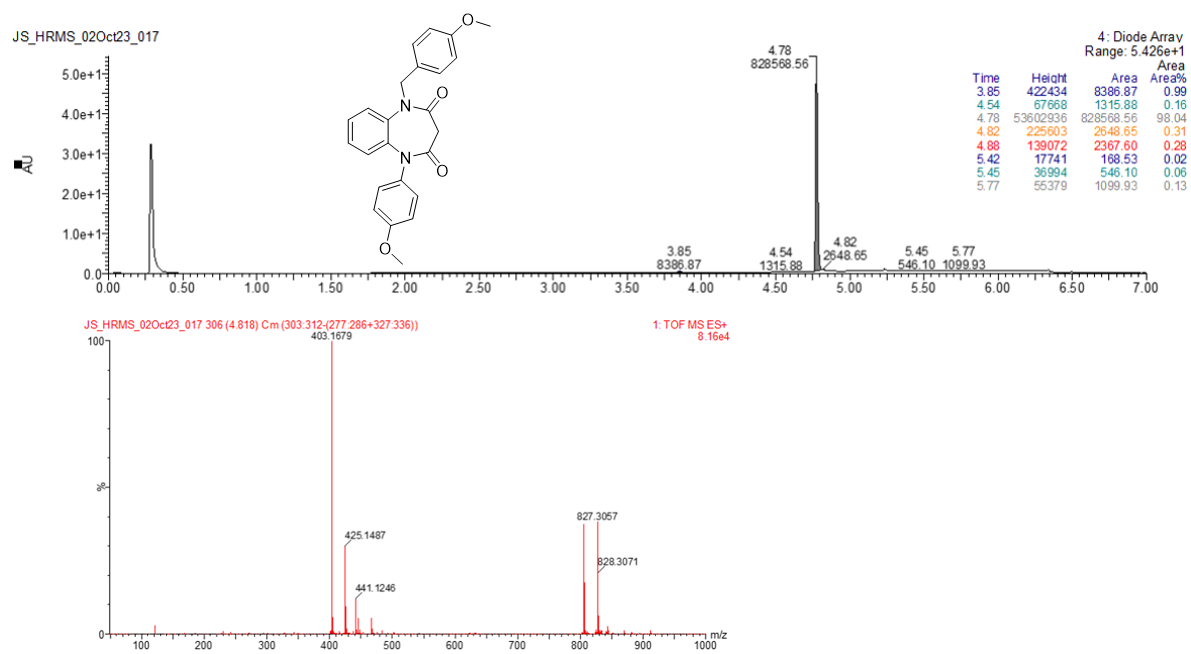
***

***In vitro* assays**

***T. cruzi* intracellular potency and rate-of-kill assay (high content imaging) ^1^**

Silvio X10/7 *T. cruzi* trypomastigotes were incubated at 37°C 5% CO_2_ with 1.6×10^7^ Vero cells overnight at MOI10 in MEM media supplemented with 10% foetal calf serum (FCS). Extracellular parasites were removed by aspirating cell culture supernatant, and washing Vero monolayer with 10 ml serum-free MEM three times. Vero cells were then harvested by trypsinization and dispensed at 2×10^3^ per well (50 μl in MEM/ 1% FCS) into 384 well plates pre-stamped with compounds in DMSO using automated washer/ dispenser EL406 and liquid handling software (Biotek). Plates were then incubated at 37°C in 5% CO_2_ for 96 h. For rate-of-kill determination, plates were incubated for 24h, 48h, 72h and 96h.

Plates were subsequently fixed with 4% formaldehyde for 20 min, permeabilised and stained with 5 μgml^-1^ Hoechst 33342/ 0.1% Triton/ PBS Thimerosal 20 min. Automated imaging was performed by an Operetta high content imaging system using 20x objective (PerkinElmer). Images were analysed with an algorithm generated in Columbus (PerkinElmer) to segment Vero cell nuclei, Vero cell cytoplasm and parasite nuclei/ kinetoplasts, reporting number of amastigotes per Vero cell, percent infected Vero cells and total number of Vero cells. Compound potencies against *T*. *cruzi* parasites were calculated in IDBS Activitybase using percent infected Vero cells and Vero toxicity curves generated using number of host cells. All data was normalised to percent inhibition based on the raw data values for the 100% effect control (16 μM nifurtimox) and the 0% effect control (DMSO) on each plate at each time point. Curve fitting was carried out using a four-parameter equation as previously described. ^2^ For rate-of-kill experiments the percent infected cells at each compound concentration was plotted against time.

***T. cruzi* trypomastigote assay**

Trypomastigote assays were carried out with *T. cruzi* Silvio X10/7 trypomastigotes using the protocol previously described. ^1^

***T. cruzi* washout assay**

Intracellular *T. cruzi* washout assays were carried out with the Silvio X10/7 strain using the protocol previously described. ^1^

***T. cruzi* aminoacyl tRNA synthetase enzyme assays**

The pIC_50_ of compound **1** was measured in *Tc*MetRS, *Tc*HisRS and *Tc*KRS assays *(N* = 2 experimental replicates, each with *n* = 1 technical replicates) using the BIOMOL® Green assay platform previously described for *Tc*MetRS. ^3^ Specific assay conditions for the three enzymes used in this study are described below:

*Tc*MetRS assay wells contained *Tc*MetRS assay buffer (30 mM Tris; pH 8, 140 mM NaCl, 40 mM MgCl_2_, 30 mM KCl, 0.01% (v/v) Brij and 1 mM DTT) plus 80 nM *Tc*MetRS, 100 μM ATP, 50 μM L-methionine and 1 U/mL pyrophosphatase. Assays were run for 2 hours.

*Tc*HisRS assay wells contained *Tc*HisRS assay buffer (30 mM Tris; pH 8, 140 mM NaCl, 40 mM MgCl_2_, 30 mM KCl, 0.01% (v/v) Brij and 1 mM DTT) plus 50 nM *Tc*HisRS, 8 μM ATP, 5 μM L-histidine and 1 U/mL pyrophosphatase. Assays were run for 2 hours.

*Tc*KRS assay wells contained *Tc*KRS assay buffer (100 mM Tris; pH 8, 140 mM NaCl, 40 mM MgCl_2_, 30 mM KCl, 0.01% (v/v) Brij and 1 mM DTT) plus 50 nM *Tc*KRS, 14 μM ATP, 400 μM L-lysine and 0.5 U/mL pyrophosphatase. Assays were run for 6 hours.

***T. cruzi* proteasome enzyme assay**

The pIC_50_ of compound **1** was measured in a *T. cruzi* proteasome assay (*N* = 2 experimental replicates, each with *n* = 3 technical replicates) as previously described, using a recombinantly expressed Δβ1Δβ2 mutant proteasome (measuring chymotrypsin site activity only). ^4^

***T. cruzi sterol* 14α-demethylase (CYP51) enzyme assay**

Potency against *T. cruzi* CYP51 was determined using a previously described fluorescence assay. Average potency for compound 1 was determined with three experimental replicates. Potencies in Fig.3 are from single experimental replicates. ^5^

***T. cruzi* oxygen consumption assay**

Oxygen consumption was measured using *T. cruzi* epimastigotes and the MitoXpress Xtra Oxygen Consumption Assay (Agilent) as previously reported. ^6^

**Supplementary Figure**

Supplementary figure 1. Rate of kill for **1**.

Rate-of-kill for **1** against intracellular *T. cruzi* amastigotes. Intracellular *T. cruzi* amastigotes (in Vero host cells) were treated with the indicated concentrations of 1 (in µM), and number of infected host cells was determined every 24 hours over a total of 96 hours using high-content imaging and analysis. A decrease in % infected cells indicates cidal activity against the intracellular parasites.

**References**

1. MacLean, L.; Thomas, J.; Lewis, M. D.; Cotillo, I.; Gray, D.; & De Rycker, M. Development of *Trypanosoma cruzi* *in vitro* assays to identify compounds suitable for progression in Chagas’ disease drug discovery. *PLoS Neglected Tropical Diseases*, 2018, 12(7), 1 – 22.
2. De Rycker M, Hallyburton I, Thomas J, Campbell L, Wyllie S, Joshi D, et al. Comparison of a high-throughput high-content intracellular *Leishmania donovani* assay with an axenic amastigote assay. *Antimicrobial Agents and Chemotherapy*. 2013; 57(7), 2913–22, pmid:23571538.
3. Torrie L.S.; Robinson D.A.; Thomas M.G.; Hobrath J.V.; Shepherd S.M.; Post, J.M.; Ko E.J.; Ferreira R.A.; Mackenzie C.J.; Wrobel K.; Edwards D.P; Gilbert I.H.; Gray D.W.; Fairlamb A.H.; De Rycker M. Discovery of an Allosteric Binding Site in Kinetoplastid Methionyl-tRNA Synthetase. *ACS Inf. Dis*., 2020, 6, 1044 – 1057.
4. Eadsforth T.C.; Torrie L.S.; Rowland P.; Edgar E.V.; MacLean L.M.; Paterson C.; Robinson D.A.; Shepherd S.M.; Thomas J.; Thomas M.G.; Gray D.W.; Postis V.L.G.; De Rycker M.; Pharmacological and structural understanding of the *Trypanosoma cruzi* proteasome provides key insights for developing site-specific inhibitors, *J. Biol. Chem*., 2025, 301 (1), 108049.
5. Riley J.; Brand S; Voice M.; Caballero I.; Calvo D.; Read K. D. Development of a Fluorescence-based *Trypanosoma cruzi* CYP51 Inhibition Assay for Effective Compound Triaging in Drug Discovery Programmes for Chagas Disease. *PLoS Negl. Trop. Dis*. 2015, 9(9), e0004014.
6. Wall R.J.; Carvalho S.; Milne R.; Bueren-Calabuig J.A.; Moniz S.; Cantizani-Perez J.; MacLean L.; Kessler A.; Cotillo I.; Sastry L.; Manthri S.; Patterson S.; Zuccotto F.; Thompson S.; Martin J.; Marco M.; Miles T.J.; De Rycker M.; Thomas M.G; Fairlamb A.H.; Gilbert I.H.; Wyllie S. The Qi Site of Cytochrome b is a promiscuous drug target in *Trypanosoma cruzi* and *Leishmania donovani*. *ACS Infect. Dis*. 2020, 6, 515–528.
